# Interventions for Young Children’s Mental Health: A Review of Reviews

**DOI:** 10.1007/s10567-023-00443-6

**Published:** 2023-07-24

**Authors:** Jennifer L. Hudson, Savannah Minihan, Wenting Chen, Talia Carl, Michele Fu, Lucy Tully, Maria Kangas, Linda Rosewell, Emma A. McDermott, Yiwen Wang, Thomas Stubbs, Alexandra Martiniuk

**Affiliations:** 1 Black Dog Institute, University of New South Wales, Sydney, Australia; 2 School of Psychological Sciences, Macquarie University, Sydney, Australia; 3 School of Psychology, University of Sydney, Sydney, Australia; 4 Faculty of Medicine and Health, University of Sydney, Sydney, Australia

**Keywords:** Mental health, Childhood, Emotional problems, Behavioral problems, Social problems, Intervention

## Abstract

To determine the efficacy of intervention programs for young children (4–9 years) with emerging mental health needs, we conducted a review of meta-analytic and systematic reviews of the intervention literature. Of 41,061 abstracts identified and 15,076 screened, 152 review articles met the inclusion criteria. We reviewed interventions across multiple disciplines targeting: (1) general mental health concerns; (2) internalizing symptoms; (3) externalizing symptoms; (4) anxiety; (5) depression; (6) trauma; (7) symptoms of attention-deficit/hyperactivity disorder; and (8) mental health concerns associated with autism spectrum disorder. Substantial evidence was found for the efficacy of behavioral and cognitive behavioral interventions for general mental health concerns, externalizing symptoms (generally, as well as ADHD, conduct, and other behavioral symptoms) and internalizing symptoms (generally, as well as anxiety) aged 4–9 years. Emerging evidence was identified for interventions targeting trauma symptoms, depression symptoms, and social, emotional and behavioral symptoms in autism spectrum disorder in children aged 4–9 years. Currently there is only limited emerging evidence regarding non-behavioral or non-cognitive behavioral interventions for programs targeting children ages 4–9 years where the aim is to deliver an evidence-based program to improve child social, emotional and/or behavioral functioning. Given the recent rises in mental health needs reported in children, targeted behavioral-and/or cognitive behavior therapy-based interventions should be made widely available to children (and their families) who experience elevated symptoms.

**Supplementary Information:**

The online version contains supplementary material available at 10.1007/s10567-023-00443-6.

Mental health disorders are common in children and consequently pose a major public health burden. One epidemiological study estimated 12-month prevalence of mental health disorders for 4–11 year olds at 13.6%, the most common class of disorders being attention deficit and hyperactivity disorder (ADHD) and anxiety disorders (Lawrence et al., 2016). Indeed, childhood behavioral and emotional disorders greatly impact children’s overall wellbeing and are responsible for significant years lost due to disability (Guthold et al., 2021). Stemming from a misconception that mental disorders do not onset until adolescence, impairment and distress in children are often ignored and overlooked. Despite advances in research and clinical service provision, there has been little population level reduction in the prevalence of mental health disorders in young people over recent decades (Sawyer et al., 2018).

One approach to reducing the incidence and burden of childhood mental health disorders is through the identification of children who experience elevated symptoms and delivery of targeted interventions designed to reduce symptoms. Interventions, delivered early in the individual’s life and illness course, can be viewed as both prevention and treatment. For children with subclinical symptoms, targeted interventions can be classified as prevention (more specifically, indicated prevention), because they are delivered before the onset of a disorder (Mrazek & Haggerty, 1994), serving to prevent symptoms from developing into a clinical disorder. They can also have a preventive effect on disorders that the child would otherwise have developed later in childhood, adolescence, or adulthood. For children with elevated symptoms severe enough to warrant a diagnosis, interventions serve as treatment for existing clinical symptoms, as well as prevention for future disorders yet to develop.

Existing reviews of the efficacy of targeted interventions for children tend to have applied a narrow focus on specific domains, such as externalizing symptoms, rather than being inclusive of a broad range of mental health problems that may emerge in childhood. Further, reviews to date have not focused on younger children specifically (i.e., 4–9 years)—an important developmental period when children first transition to formal schooling. This period provides a key window for the detection of early social, emotional, and behavioral problems, and the delivery of effective early intervention. Indeed, there are many mental health interventions delivered in schools that are targeted to young children, yet not all programs are evidence-based (Laurens et al., 2022), perhaps representing the lack of literature on evidence-based interventions for younger children specifically.

As such, the aim of this study was to evaluate the efficacy of a range of interventions designed for children 4–9 years covering mental health difficulties broadly, as well as shared mechanisms and disorder-specific interventions. Broad-based interventions alone aggregate effects across various mental health difficulties by targeting shared mechanisms or general distress which may not provide a sufficient dose to address specific mental health concerns. Reviewing targeted programs for mental health difficulties more broadly, along with programs that target shared mechanisms, and disorder-specific difficulties may allow decision-makers to flexibly adapt interventions to various mental health needs.

Specifically, this review aimed to evaluate the efficacy of early interventions including both indicated preventions and treatments designed for children aged 4–9 years covering: broad mental health problems; internalizing symptoms; externalizing symptoms; anxiety; depression; mental health symptoms related to exposure to trauma; symptoms of attention-deficit/hyperactivity, and mental health symptoms related to autism spectrum disorder. Due to the enormity of this literature, we chose to conduct a systematic review of existing systematic reviews and meta-analyses. The objective was to identify evidence-based approaches, which may be used to assist schools and governments in making decisions about mental health programs. We chose to evaluate interventions targeted at young children aged 4–9 years (and their parents), as this represents a key developmental period of early schooling, for delivery by health and education professionals. Furthermore, we conducted an exploratory narrative synthesis of moderators when available within the texts. To increase the relevance of the findings to practitioners, we engaged an implementation partner, the NSW Department of Health, a state government department responsible for delivering mental health programs in primary schools within one state in Australia.

## Methods

### Search Strategy

A review of the English-language, peer-reviewed published literature was conducted and included reviews published over the last 23 years (January 2000–May 2023). The search identified systematic reviews and meta-analyses evaluating interventions for emotional, behavioral, and/or social problems in children aged 4- to 9-years. Reviews targeting a broader age range were included if the mean age fell within the 4–9 age range. We chose an iterative clinician-led process to shape the search terms to ensure the review included interventions used by the clinicians in community practice. This made registration of the review impossible as data extraction needed to take place to inform each consultation prior to finalizing the final search strategy.

### Databases Searched

We searched seven electronic databases (PsycInfo, PubMed, Cochrane Library, CINAHL, ERIC, Family and Society Studies Worldwide, and Violence and Abuse Abstracts). The search terms were developed by identifying terms and synonyms corresponding to a range of common mental health problems in children. Mental health problems were defined broadly to include anxiety, obsessive compulsive disorder (OCD), depression, suicide/self-harm, conduct problems, sleep problems, emotion dysregulation, ADHD, social skills problems, attachment problems, childhood trauma, sensory regulation, or social-emotional wellbeing. We did not include interventions that were specifically designed to target Autism Spectrum Disorder (ASD) unless they targeted any of the above mental health problems specifically in children with ASD. Mental health terms were combined with terms and synonyms for “interventions.” Additional search terms were included to ensure a sensitive inclusion for programs also designed for First Nations communities. The searches were restricted to meta-analyses and systematic reviews and to populations of, or including, children aged 4- to 9-years. The detailed search strategy is provided in Supplementary Materials 1. The detailed inclusion and exclusion criteria are provided in Supplementary Material 2.

To ensure that the inclusion criteria and databases aligned with the needs of health professionals, the methods including search terms, databases and inclusion exclusion criteria were informed, reviewed, and approved by a Clinical Advisory group (Beames et al., 2021). The group was made up of a range of allied health professionals with experience delivering mental health interventions to children with social, emotional and behavioral problems in the first three years of school. The Clinical Advisory group conducted fortnightly consultations to determine the best scope of this review, integrating their experience with current mental health programs delivered in this age group.

### Screening

Screening was completed on Covidence (https://www.covidence.org/). The titles and abstracts of the articles initially identified by the searches were screened to determine their relevance to the review. At this stage, irrelevant articles were excluded. Two members of the research team independently completed title and abstract screening on the remaining articles. The interrater reliability for title and abstract screening was moderate (*κ* = 0.62). Any discrepancies were resolved via discussion. The full text for each retained article was then examined according to the inclusion/exclusion criteria. One member of the research team completed full text review. A second member of the research team checked all excluded full text articles to ensure agreement on exclusion criteria. When disagreement about inclusion or exclusion occurred, consensus was reached through additional review and discussion. Interrater reliability for full-text screening was substantial (*κ* = 0.80).

### Data Extraction

The following data was extracted for each review: citation, target of intervention (e.g., externalizing symptoms), intervention type (e.g., behavioral-based parent training), number of studies included, design of studies included (e.g., randomized controlled trials), total number of participants, age of participants, evidence statement, review design (e.g., systematic review). One member of the research team conducted data extraction. All extracted data was checked by another member of the research team and discrepancies discussed. Data were extracted in Covidence and downloaded to Excel.

### Quality Appraisal

GRADE was used to assess the quality of evidence for each included review (Guyatt et al., 2008, 2011). Four levels of quality make up the GRADE score, with a ‘very low’ score meaning the true effect is different from that found in the research presented; and a ‘high’ score meaning that there is greater confidence in the findings presented in the research. For each article, individual GRADE scores are provided in Table 1 and a written rationale for that score in Table 2. All GRADE scores were checked by another member of the research team.

**Table 1:** Description of included studies

Authors	Target of intervention	Intervention type	Number of studies included	Design	Total number of participants	Age range of participants; mean age in years [Subgroup]	Review design	GRADE
ADHD								
Arnold et al. (2015)	ADHD	Pharmacological and psychosocial interventions	51	Mixed randomized controlled trials, non-randomized trials, single-group cohort studies	NR	6–18 + ; NR [6–12; NR]	Systematic review	Moderate
Bjornstad & Montgomery (2005)	ADHD	Family therapy	2	Randomized controlled trials	NR	NR; NR	Systematic review	Low
Brooks & Bannigan (2021)	ADHD	Play-based occupational therapies	9	Non-randomized trials	100	5–16; NR	Systematic review	Low
Coates et al. (2015)	ADHD	Parenting interventions (behavioral based)	11	Mixed randomized controlled trials & non-randomized controlled trials	603	2.75–12; NR [3–5]	Meta-analysis and systematic review	Moderate
Corcoran & Dattalo (2006)	ADHD	Psychosocial interventions	16	Mixed randomized controlled trials and non-randomized controlled trials	NR	0–18; NR	Meta-analysis	Low
Cornell et al. (2018)	ADHD	Play-based occupational therapies	7	Mixed non-randomized controlled trials, single-group cohort studies, single subject design	142	5–11; 7.6	Systematic review	Moderate
Fabiano et al. (2009)	ADHD	Behavioral interventions	174	Mixed randomized controlled trials, non-randomized trials, single-group cohort studies, case studies	2087	7.1–8.9; NR	Meta-analysis and systematic review	Moderate
Fox et al. (2020)	ADHD	Social skills interventions (Peers)	15	Mixed randomized controlled trials, non-randomized trials, single-group cohort studies, case studies	600	5–16; NR	Systematic review	Moderate
Gaastra et al. (2016)	ADHD	Behavioral interventions	89	Mixed randomized controlled trials, non-randomized trials, single-group cohort studies, case studies	627	6–17; NR [6–11]	Meta-analysis	Moderate
Ghuman et al. (2008)	ADHD	Pharmacological and psychosocial interventions	45	Mixed randomized controlled trials, non-randomized trials, single-group cohort studies, case studies	2465	0–12; NR	Systematic review	Low
Groenman et al. (2022)	ADHD	Behavioral treatments	25	Randomized controlled trials	2885	2–17.5;8.78	Meta-analysis	High
Harrison et al. (2019)	ADHD	Psychosocial interventions (School-based)	27	Single-case studies	49	NR; NR [K-5grade]	Meta-analysis and systematic review	Low
Hodgson et al. (2014)	ADHD	Psychosocial interventions	14	Mixed randomized controlled trials, non-randomized trials, single-group cohort studies	625	5.1–10.5; 8.6	Meta-analysis	Moderate
Hornstra et al. (2023)	ADHD	Behavioral interventions (parent and teacher training)	32	Randomized controlled trials	2594	2–18; NR	Meta-analysis	High
Iznardo et al. (2020)	ADHD	Behavioral interventions	7	Mixed randomized controlled trials, non-randomized trials, single-group cohort studies	272	3–18; 7.9	Meta-analysis	Moderate
Krisanaprakornkit et al. (2010)	ADHD	Meditation therapies	4	Randomized controlled trials	83	6–13; NR	Systematic review	Low
Lee et al. (2012)	ADHD	Parenting interventions (behavioral based)	40	Mixed randomized controlled trials and non-randomized controlled trials	2357	3.34–14.68; NR	Meta-analysis	Low/Moderate
McGoey et al. (2002)	ADHD	Pharmacological and psychosocial interventions	26	Non-randomized trials, single-group cohort studies	820	3–5.9; NR	Systematic review	Low
Mulqueen et al. (2015)	ADHD	Parenting interventions (behavioral based)	8	Randomized controlled trials	399	3–5.36; NR	Meta-analysis	High
Murray et al. (2018)	ADHD	The incredible years	11	Randomized controlled trials	1352	3–8; NR	Systematic review	High
Pauli-Pott et al. (2021)	ADHD and externalizing symptoms	Cognitive interventions	35	Randomized controlled trials	3068	3,0–6,11; NR	Meta-analysis	High
Pyle & Fabiano (2017)	ADHD	School based behavioral intervention (daily report cards)	14	Single-case studies	40	4–14; NR	Meta-analysis	Very low
Reid et al. (2005)	ADHD	Self-regulation interventions	16	Single-group cohort studies, Single-case studies	51	6–15; NR [< 12]	Meta-analysis	Low
Riise et al. (2021)	Externalizing disorders and ADHD	Cognitive behavioral interventions	51	Mixed Randomized controlled trials, Single-group cohort studies	5295	2–17; 8.2	Meta-analysis and systematic review	Moderate/High
Rimestad et al. (2019)	ADHD	Parenting interventions (behavioral based)	16	Randomized controlled trials	1003	2.5–6; NR	Meta-analysis and systematic review	High
Storebo et al. (2019)	ADHD: (Social skills)	Social skills interventions	25	Randomized controlled trials	2690	5–17; NR	Meta-analysis and systematic review	High
Tan-McNeill et al. (2021)	ASD, ADHD (and other neurodevelopmental disorders)	Parenting interventions (Digital)	11 (for ASD and ADHD)	Randomized controlled trials, Single-group cohort studies, Single case studies	209 (ASD group), 313 (ADHD group)	1.7–16; NR	Systematic review	Low
Türk et al. (2023)	ADHD	Psychological Interventions (and pharmacological)	16	Meta-analyses (14 meta-analyses included only RCTs, and two also included non-randomized trials)	18,224	4.3–10.5	Meta-analysis and systematic review	High
Vacher et al. (2020)	ADHD	Psychosocial interventions	12	Mixed randomized controlled trials, non-randomized trials, single-group cohort studies	1287	5–17; NR	Systematic review	Moderate
Van der Oord et al. (2008)	ADHD	Pharmacological and psychosocial interventions	26	Randomized controlled trials	1482	6–12; NR	Meta-analysis	High
Vekety et al. (2021)	ADHD	Mindfulness	21	Mixed Randomized controlled trials and non-randomized trials	1792	3–12; NR	Meta-analysis	Moderate
Vetter (2018)	ASD or ADHD	Parent–Child interaction therapy	18	Mixed non-Randomized controlled trials, single-group cohort studies	93	2–12; NR	Systematic review	Low
Wilkes-Gillan et al. (2021)	ADHD	Behavioral interventions	15 (incl. 4 follow-up)	Randomized controlled trial, Non-randomized trials, single-group cohort studies, single case studies	106	5–16; NR	Systematic review	Low/Moderate
Willis et al. (2019)	ADHD (Social skills)	Social skills interventions	16	Mixed Randomized controlled trials & single-group cohort studies	NR	5–16; NR	Systematic review	Moderate
Zwi et al. (2011)	ADHD	Parenting interventions (behavioral based)	5	Randomized controlled trials	284	4–13; NR	Meta-analysis and systematic review	Moderate
Anxiety								
Ale et al. (2015)	Anxiety (+ OCD)	Cognitive behavioral interventions	43	Randomized controlled trials	2791	5–18; NR	Meta-analysis	Moderate
Bennet et al. (2013)	Anxiety	Cognitive behavioral interventions	16	Randomized controlled trials	1171	6–19, NR	Meta-analysis and systematic review	High
Caldwell et al. (2019)	Anxiety or depression	Psychosocial interventions (School-based)	109	Mixed randomized controlled trials and non-randomized controlled trials	56,620	NR; NR [Primary School)	Meta-analysis	Moderate
Comer et al. (2019)	Anxiety	Psychosocial interventions	30	Mixed Randomized controlled trials, Non-randomized trials, Single -group cohort studies	2228	NR; < 7.9 years	Systematic review	Moderate
Fisak et al. (2011)	Anxiety	Psychosocial interventions	35	Mixed randomized controlled trials, non-randomized trials, single group cohort studies, Case studies	7735	< 18; NR	Meta-analysis	Moderate
Grist et al. (2019)	Anxiety and depression	Psychosocial interventions (Digital)	34	Randomized controlled trials	3113	6–18; NR	Meta-analysis and systematic review	High
Howes Vallis et al. (2020)	Anxiety	Cognitive behavioral interventions	43	Mixed randomized controlled trials, non-randomized trials, single-group cohort studies	2656	3–8; 5.45	Meta-analysis and systematic review	Moderate
Krebs et al. (2018)	Anxiety	Cognitive bias modification	26	Randomized controlled trials	1786	6–18; NR	Meta-analysis and systematic review	High
McGuire et al. (2015)	OCD	Pharmacological and cognitive behavioral interventions	20	Randomized controlled trials	1296	5.8–15.0; NR	Meta-analysis	High
Odgers et al. (2020)	Anxiety	Mindfulness	20	Randomized controlled trials	1582	< 18; NR	Meta-analysis	Moderate
Ostergaard (2018)	Selective mutism	Pharmacological and cognitive behavioral interventions	15	Mixed randomized controlled trials, non-randomized trials, single-group cohort studies	134	5–14; 5.98	Systematic review	Low
Phillips & Mychailyszyn (2021)	Anxiety	Parent–Child interaction therapy	15	Randomized controlled trials, Single-group cohort studies	370	2–9.75; NR	Meta-analysis	Low
Reynolds et al. (2012)	Anxiety	Psychosocial interventions	55	Randomized controlled trials	4258	2–19; NR [< 14; NR]	Meta-analysis and systematic review	High
Steains et al. (2021)	Selective mutism	Psychosocial intervention (Combination behavioral and systems treatments)	5	Randomized controlled trials	233	3–18; 7.0	Meta-analysis	Moderate
Viswanathan et al. (2022)	Anxiety	Cognitive behavioral interventions	29 (3 studies in ages 3–7)	Randomized controlled trials	2805	4.1–17.4; NR [3–7; NR]	Systematic review	Moderate
Werner-Seidler et al. (2017)	Anxiety and depression	Psychosocial interventions (School-based)	81	Randomized controlled trials	31,794	5–19; NR	Meta-analysis and systematic review	High
Werner-Seidler et al. (2021)	Anxiety and depression	Psychosocial interventions (School-based)	130	Randomized controlled trials	45,924	5–19; NR [< 10; NR]	Meta-analysis and systematic review	High
Yin et al. (2021)	Anxiety	Cognitive behavioral interventions (Parent)	6	Randomized controlled trials	407	2.7–14; 8	Meta-analysis	Moderate
ASD								
Aldabas (2019)	ASD: (Inappropriate social behavior)	Social stories	22	Case series	56	3–15; 8	Systematic review and meta analysis	Very low
Camargo et al. (2014)	ASD: (Social skills)	Behavioral-based intervention	30	Mixed single-case designs, single-group cohort studies	55	3–21;5	Systematic review	Very low
Camargo et al. (2016)	ASD: (Social skills)	Behavioral interventions	19	Single case studies	55	2–18 +; NR	Meta-analysis	Very low
Gunning et al. (2019)	ASD (Social skills)	Social skills interventions	57	Single case studies	152	0–6; NR	Systematic review	Very low
Kokina & Kern (2010)	ASD: (Social skills, inappropriate behavior)	Social Stories	18	Single case studies	47	3–15; NR	Meta-analysis	Very low
Reichow et al. (2013)	ASD (Social skills)	Social skills interventions	5	Randomized controlled trials	178	6–21;NR[8–11; NR]	Meta-analysis and systematic review	High
Slaughter et al. (2020)	ASD (Anxiety)	Psychosocial interventions	15	Meta-analyses, Systematic reviews, Evidence-based Guides, Websites	Not reported	< 18 years; NR	Systematic review	Low
Tan-MacNeill et al. (2021)	ASD, ADHD (and other neurodevelopmental disorders)	Parenting interventions (Digital)	11 (for ASD and ADHD)	Mixed Randomised controlled trials, single group cohort studies, single case studies	209 (ASD group), 313 (ADHD group)	1.7–16; NR	Systematic review	Low
Tarver et al. (2019)	ASD (Externalizing and Internalizing)	Parenting interventions (behavioral based)	9	Randomized controlled trials	466	2–14; NR	Meta-analysis and systematic review	High
Vetter (2018)	ASD or ADHD	Parent–Child interaction therapy	18	Mixed non-Randomized controlled trials, single-group cohort studies	93	2–12; NR	Systematic review	Low
Wahmann et al. (2022)	ASD (Social skills)	Social Stories	12	Single-case studies	30	2:6–10:3; 5:3	Systematic review and meta-analysis	Very low
Wang et al. (2011)	ASD: (Social skills)	Social skills interventions (Peer-mediated)	14	Single case studies	43	4–15; 6.49	Meta-analysis	Very low
Wang et al. (2013)	ASD: (Social skills)	Social skills interventions (Peer-mediated)	115	Single case studies	343	0.75–32; 6.51	Meta-analysis	Low
Wang & Spillane (2009)	ASD: (Social skills)	Social skills interventions	38	Single case designs, non-controlled trials	147	2–17; NR	Meta-analysis	Very low
Weitlauf et al. (2017)	ASD (Sensory Challenges)	Sensory-based interventions	24	Mixed randomized controlled trials, non-randomized trials, single-group cohort studies	1010	4.54–9.42; NR	Systematic review	Moderate
Whalon et al. (2015)	ASD: (Social skills)	Social skills interventions (Peer-mediated)	37	Single case studies	105	3–12; 6.38	Systematic review and Meta analysis	Very low
Wright et al. (2016)	ASD (social skills)	Social Stories	99	Single-case studies, between-group designs, other	NR	NR;NR	Systematic review	Very low
Depression								
Benarous et al. (2017)	Disruptive mood dysregulation disorder or severe mood dysregulation	Pharmacological and psychosocial interventions	15	Mixed Randomized controlled trials, Single-group cohort studies, Case studies	203	5–18; NR	Systematic review	Low
Caldwell et al. (2019)	Depression or anxiety	Psychosocial interventions (School-based)	109	Mixed randomized controlled trials and non-randomized controlled trials	56,620	NR; NR [Primary School)	Meta-analysis	Moderate
Cuijpers et al. (2023)	Depression	Psychosocial interventions	40	Randomized controlled trials	3779	4.3–17.5; NR	Meta-analysis and systematic review	High
Forti-Buratti et al. (2016)	Depression	Psychosocial interventions	7	Randomized controlled trials	219	0–12; NR	Meta-analysis and systematic review	Low
Michael & Crowley (2002)	Depression	Pharmacological and psychosocial interventions	38	Mixed randomized controlled trials, non-randomized trials, single group cohort studies, Case studies	1499	5–19; NR	Meta-analysis	High
Werner-Seidler et al. (2017)	Depression and anxiety	Psychosocial interventions (School-based)	81	Randomized controlled trials	31,794	5–19; NR	Meta-analysis and systematic review	High
Werner-Seidler et al. (2021)	Anxiety and depression	Psychosocial interventions (School-based)	130	Randomized controlled trials	45,924	5–19; NR [< 10; NR]	Meta-analysis and systematic review	High
Externalizing								
Bakker et al. (2017)	Conduct disorder	Psychosocial interventions	17	Randomized controlled trials	1999	2.8–16.8; 7.5	Meta-analysis	Moderate
Barlow & Stewart-Brown (2000)	Behavioral problems	Parenting interventions (Group)	16	Mixed randomized controlled trials and non-randomized controlled trials	1792	0–14; NR	Systematic review	Moderate
Battagliese et al. (2015)	Externalizing disorders	Cognitive behavioral interventions	21	Randomized controlled trials	1960	NR, 7	Meta-analysis	High
Baumel et al. (2016)	Behavioral problems	Parenting interventions (Digital)	7	Randomized controlled trials	718	2–18, NR	Meta-analysis and systematic review	High
Baumel et al. (2017)	Behavioral problems	Parenting interventions (Digital)	14	Mixed randomized controlled trials, non-randomized trials, single-group cohort studies	2427	2–15; NR	Systematic review	Moderate
Burkey et al. (2018)	Behavioral problems	Psychosocial interventions	26	Randomized controlled trials	4441	0–18, NR	Meta-analysis and systematic review	High
Cai et al. (2022)	Externalizing disorders	Parenting interventions (behavioral based)	20	Mixed Randomized controlled trials, Non-randomized trials	3983	5.9–11.8 years	Meta-analysis and systematic review	Moderate
Comer et al. (2013)	Externalizing symptoms	Psychosocial interventions	36	Randomized controlled trials	3042	2–7.7, 4.7	Meta-analysis	High
Connor et al. (2006)	Behavioral problems	Pharmacological and psychosocial interventions	180	Mixed randomized controlled trials, non-randomized trials, single-group cohort studies, Meta-analyses	NR	0–18, NR	Systematic review	High
de Graaf et al. (2008)	Externalizing symptoms	Triple P	15	Mixed randomized controlled trials and non-randomized controlled trials	2513	2–12, NR	Meta-analysis	High
Dedousis-Wallace et al. (2021)	Behavioral problems	Parenting interventions (behavioral based)	21	Randomized controlled trials	NR	3–14, NR	Systematic review	High
Dretzke et al. (2005)	Behavioral problems	Parenting interventions	37	Randomized controlled trials	2581	0–18; NR	Meta-analysis and systematic review	High
Dretzke et al. (2009)	Behavioral problems	Parenting interventions	57	Randomized controlled trials	NR	0–12; NR	Systematic review	High
Florean et al. (2020)	Behavioral problems	Parenting interventions (Digital)	15	Randomized controlled trials	1668	2–18, NR	Meta-analysis and systematic review	High
Forster et al. (2012)	Behavioral problems	Parenting interventions (behavioral based)	8	NR	932	NR, NR	Meta-analysis and systematic review	Moderate
Fossum et al. (2008)	Behavioral problems	Psychosocial interventions	65	Mixed randomized controlled trials, non-randomized trials, single-group cohort studies	4971	NR, NR	Meta-analysis and systematic review	Moderate
Fossum et al. (2016)	Behavioral problems	Psychosocial interventions	56	Mixed randomized controlled trials, non-randomized trials, single-group cohort studies	2589	2–17; NR	Meta-analysis	Moderate
Furlong et al. (2012)	Behavioral problems	Parenting interventions (behavioral based)	13	Mixed randomized controlled trials and non-randomized controlled trials	1078	3–12; 5.3	Meta-analysis and systematic review	Moderate
Gardner et al. (2019a)	Behavioral problems	The incredible years	13	Randomized controlled trials	1696	2–10; NR	Meta-analysis and systematic review	High
Gardner et al. (2019b)	Behavioral problems	Parenting interventions	169	Randomized controlled trials	15,074	2–10; 5.3	Meta-analysis	Low/Moderate
Lane et al. (2023)	Conduct problems	Psychosocial interventions	13	Randomized controlled trials	858	2–12; NR	Systematic review	Low
Leijten et al. (2013)	Behavioral problems	Parenting interventions (behavioral based)	75	Mixed randomized controlled trials and non-randomized controlled trials	4277	0–12; NR	Meta-analysis and systematic review	Moderate
Leijten et al. (2016)	Externalizing	Parenting interventions (behavioral based)	129	Randomized controlled trials	13,091	0–16; NR	Meta-analysis and systematic review	High
Leijten et al. (2018)	Externalizing	Parenting interventions	197	Randomized controlled trials	15,768	1–11; 4.93, 5.54	Meta-analysis and systematic review	High
Leijten et al. (2020)	Behavioral problems	The incredible years	13	Randomized controlled trials	1696	2–10; 5.26	Meta-analysis (IPDMA)	High
Losel & Beelmann (2003)	Behavioral problems	Social skills interventions	84	Randomized controlled trials	16,723	4–18; NR	Meta-analysis and systematic review	High
Maughan et al. (2005)	Externalizing	Parenting interventions (behavioral based)	79	Mixed randomized controlled trials, non-randomized trials, single group cohort studies, Case studies	2570	3–16; NR	Meta-analysis	High
Menting et al. (2013)	Behavioral problems	The incredible years	50	Mixed randomized controlled trials and non-randomized controlled trials	4745	3–9.2-NR	Meta-analysis and systematic review	Moderate
Mingebach et al. (2018)	Externalizing	Parenting interventions	26	Meta-analyses	NR	0–18; NR	Meta-analysis	Moderate
Nogueira et al. (2022)	Behavioral problems	Triple P	11	Randomized controlled trials	885	2–12 (5.2)	Meta-analysis and systematic review	Moderate
Nye et al. (2019)	Externalizing	Incredible years	9	Randomized controlled trials	5759	3–8; NR	Systematic review	High
Parker et al. (2021a)	Behavioral problems	Child-centered play therapy	23	NR (between-group studies; could be Randomized controlled trials and/or non-randomized trials)	908	3–11 (median = 6)	Meta-analysis	Moderate
Riise et al. (2021)	Externalizing disorders and ADHD	Cognitive behavioral interventions	51	Mixed Randomized controlled trials, Single-group cohort studies	5295	2–17; 8.2	Meta-analysis and systematic review	Moderate/High
Smith et al. (2021)	Mental health symptoms (inattention, conduct problems, reading problems, peer relations)	Psychosocial interventions	7	Randomized controlled trials	4009	6–7; NR	Meta-analysis	Moderate
Solomon et al. (2017)	Behavioral problems	Parenting interventions	15	Mixed randomized controlled trials and non-randomized controlled trials	1400	NR	Meta-analysis and systematic review	Moderate
Stoltz et al. (2012)	Externalizing	Psychosocial interventions (School-based)	24	Mixed randomized controlled trials and non-randomized controlled trials	1894	NR; 7.85, 8.03	Meta-analysis and systematic review	High
Tarver et al. (2014)	Externalizing	Parenting interventions (Self-guided)	11	Randomized controlled trials	NR	2–12 years; NR	Meta-analysis and systematic review	High
Thongseiratch et al. (2020)	Behavioral problems	Parenting interventions (Digital)	12	Randomized controlled trials	2025	2–12; NR	Meta-analysis and systematic review	High
Tse (2006)	Behavioral problems	Psychosocial interventions	5	Mixed randomized controlled trials, non-randomized trials, single-group cohort studies	149	2.5–6; NR	Systematic review	Low
Tully and Hunt (2016)	Externalizing	Parenting interventions (behavioral based)	8	Randomized controlled trials	836	2–12; NR	Systematic review	High
Uretsky & Hoffman (2017)	Externalizing	Parenting interventions (behavioral based)	7	Mixed randomized controlled trials, non-randomized trials, single-group cohort studies	2830	4–18; NR	Meta-analysis and systematic review	Moderate
Veenman et al. (2018)	Externalizing	Behavioral interventions	19	Randomized controlled trials	18,094	NR; NR	Meta-analysis and systematic review	Moderate
Ward et al. (2016)	Externalizing	Parent–Child interaction therapy	12	Mixed randomized controlled trials, non-randomized trials, single group cohort studies	372	2–5; NR	Meta-analysis and systematic review	Moderate
Ye et al. (2021)	Externalizing	Music interventions	10	Mixed randomized controlled trials and non-randomized controlled trials	3465	6–16; NR	Meta-analysis and systematic review	Moderate
Internalizing								
Sun et al. (2019)	Internalizing	Cognitive behavioral interventions	76	Randomized controlled trials	NR	< 18 years; NR [< = 6; 7–12]	Meta analysis	High
Yap et al. (2016)	Internalizing	Parenting interventions	42	Randomized controlled trials	NR	0–18; NR	Meta-analysis	Moderate
Mental Health								
Bauer et al. (2021)	Mental health symptoms	Psychosocial intervention (socio-emotional, mobilizing social support)	13	Mixed NR specifics (no restrictions on design)	NR	3–9; NR	Systematic review	Low
Bayer et al. (2009)	Mental health symptoms (externalizing, internalizing or both)	Psychosocial interventions	59	Randomized controlled trials	NR	0–8, NR	Systematic review	High
Benoit & Gabola (2021)	Mental health symptoms (social-emotional wellbeing)	Psychosocial intervention (positive psychology)	3	Mixed Non-randomized trials, single-group cohort studies	561	4–12; NR (majority 4–9)	Systematic review	Low
Blewitt et al. (2021)	Mental health symptoms (social-emotional wellbeing)	Psychosocial intervention (school-based, emotional competency)	19	Randomized controlled trials, Non-randomized trials, single-subject designs	1944	0–6; NR	Systematic review	Low/Moderate
Bratton et al. (2005)	Internalizing and/or Externalizing	Play Therapy	93	Mixed randomized controlled trials and non-randomized controlled trials	3248	6.7–7; NR	Meta-analysis	Moderate
Buchanan-Pascall et al. (2018)	Internalizing or Externalizing	Parenting interventions (Group)	23	Randomized controlled trials	2197	4–12; NR	Meta-analysis and systematic review	High
Carr et al. (2017)	Mental health symptoms	Parents Plus	17	Mixed randomized controlled trials, non-randomized controlled trials, single group cohort studies	1562	2–17; NR	Meta-analysis and systematic review	Low/moderate
Dalgaard et al. (2022)	Broad child mental health (attachment)	Parenting interventions	25	Randomized controlled trials, Non-controlled trials	1302	0.62–10.65; 5.15	Systematic review	Moderate
England-Mason et al. (2023)	Mental health symptoms (emotional competence)	Parenting interventions	15	Randomized controlled trials	NR	2–6; NR	Meta-analysis and systematic review	High
Everett et al. (2021)	Broad child mental health	Psychosocial interventions	56	Randomized controlled trials	NR	1–18; NR [3–5 and 6–11; NR)	Systematic review	Moderate
Jugovac et al. (2022)	Broad mental health problems (externalizing and internalizing behaviors)	Parenting interventions	43	Randomized controlled trials, Non-randomized trials	5542	0–18;7.14	Meta-analysis and systematic review	High
Law et al. (2012)	Broad child mental health (children with communication difficulties and related behavioral issues)	Behavioral intervention	19	Single case studies, single cohort studies, one non-controlled study	148	3–13; NR	Systematic review	Very low
Ledford et al. (2023)	Mental health symptoms (social skills problems)	Social skills intervention (play-based therapy)	9	Non-randomized trials, single case studies	25 focal, 31 peer participants	36–90 months (3–7.5 years); 56 months (4.67 years)	Meta-analysis and systematic review	Low
McDonald & Drey (2018)	Mental health symptoms	Art therapy	4	Mixed randomized controlled trials & non-randomized trials	205	7–13; NR	Systematic review	Low/Moderate
Money et al. (2021)	Mental health symptoms	Child-centered play therapy	6	Randomized controlled trials, single group cohort studies	362	4.1–10.34; NR	Systematic review	Low
Moula (2020)	Generic child outcomes	Art therapy	6	Mixed randomized controlled trials and non-randomized controlled trials	247	6–14; NR	Systematic review	Low
Moula et al. (2020)	Generic child outcomes	Art therapy	7	Mixed randomized controlled trials, non-randomized trials, single-group cohort studies	358	6–13:NR	Systematic review	Moderate
Pester et al. (2019)	Mental health symptoms	Child-centered play therapy	11	Single case studies	43	3–10; NR	Meta-analysis and systematic review	Very low
Pilling et al. (2020)	Mental health symptoms	Psychosocial interventions	138	Randomized controlled trials	14,954	4–18; NR [4–12; NR]	Meta-analysis and systematic review	Moderate
Sanchez et al. (2018)	Broad child mental health	School-based Psychosocial interventions	43	Randomized controlled trials	49,941	grades K-6; mean grade 2.86	Meta-analysis	High
Savaglio et al. (2023)	Mental health symptoms (with a focus on internalizing and/or externalizing symptoms)	Psychosocial interventions	42	Randomized controlled trials, non-randomized trials, single group cohort studies	5964	1–9; 5.78	Meta-analysis and systematic review	Moderate
Schleider (2017)	Mental Health Disorders	Psychosocial interventions	50	Randomized controlled trials	10,508	< 19 years; NR [< 11; NR]	Meta-analysis	High
Sheridan et al. (2019)	Mental health symptoms	Parenting interventions (behavioral based)	117	Mixed randomized controlled trials, non-randomized trials, single group cohort studies	37,769	NR; NR	Meta-analysis	Moderate
Shucksmith et al. (2010)	Mental health symptoms	Parenting interventions	59	Randomized controlled trials	Not reported	4–11; NR	Systematic review	Moderate/High
Sprung et al. (2015)	Emotional understanding	Emotional competency training	19	Mixed randomized controlled trials, non-randomized controlled trials, single group cohort studies	1308	2.9–17.25; 7.2	Meta-analysis	Moderate
Sun et al. (2021)	Mental health symptoms (social emotional function and regulatory skills)	Mindfulness	16	Mixed Randomized controlled trials, non-controlled trials, single group cohort study	3584	3–5.4; NR	Systematic review	Low/Moderate
Zarakoviti et al. (2021)	Behavioral disorders with comorbid internalizing symptoms	Parenting interventions	12	Randomized controlled trials	1334	2–10;5	Systematic review	Moderate
Trauma								
Bastien et al. (2020)	PTSD	Psychosocial interventions	27	Randomized controlled trials	1206	3–25; NR	Meta-analysis and systematic review	High
Hambrick et al. (2016)	Mental health symptoms (incl trauma)	Psychosocial interventions (foster care)	39	Mixed randomized controlled trials, non-randomized trials, single group cohort studies	NR	0–12; NR	Systematic review	Moderate
Humble (2019)	Trauma	Child-centred play therapy	7	Mixed randomized controlled trials, non-randomized trials, single group cohort studies	186	0–16; 7.3	Systematic review	Low
Lindstrom Johnson et al. (2018)	Trauma	Parenting interventions	21	Mixed randomized controlled trials, non-randomized trials, single group cohort studies	1361	3–17; NR	Meta-analysis and systematic review	Moderate
McGuire et al. (2021)	Trauma	Trauma-focused CBT	11	Mixed randomized controlled trials, non-randomized trials, single group cohort studies, Meta-analyses	2575	3–6; NR	Systematic review	Moderate
Parker et al. (2021b)	Trauma	Child-centred play therapy	32	Mixed randomized controlled trials and non-randomized controlled trials	1207	3.5–11; NR	Systematic review	Moderate
Purgato (2018)	Trauma	Psychosocial interventions	11	Randomized controlled trials	3143	7–18; NR	Meta-analysis and systematic review	High
Rosner et al. (2010)	Bereavement and grief	Psychosocial interventions	27	Mixed randomized controlled trials, non-randomized trials, single group cohort studies	1073	0–20, NR	Meta-analysis and systematic review	Moderate

**Table 2:** GRADE score reasonings for individual papers

Authors	GRADE score	GRADE reasoning
ADHD		
Arnold et al., (2015)	High	Risk of bias: some lack of randomization; Precision: large effect sizes observed across multiple combination studies (pharma/non-pharma). Consistent across studies. Intervention directly related to outcome/population of interest. Publication bias: not ascertained; Magnitude of effect: mostly strong; Dose response: combination therapy gradient increases GRADE
Brooks and Gannigan, (2021)	Low	Risk of bias: no RCTS, case reports and quasi-experimental studies were very low quality, lack of randomization/blinding; Precision: large effect sizes in some studies, but no high-quality evidence for occupation-based/-focused occupational therapy interventions for children and adolescents with mental health difficulties; Significant heterogeneity in quantitative data; Publication bias: not ascertained; Dose response: N/A
Bjornstad and Montgomery (2005)	Moderate	Risk of bias: all RCTS, but small sample size reduces power of study; Precision: partially meaningful, small to medium effect sizes observed; Some heterogeneity across studies; Intervention directly related to outcome/population of interest; Publication bias: not ascertained; Magnitude of effect: low to moderate; Dose response: N/A
Coates et al., (2015)	Moderate	Risk of bias: some lack of randomization/blinding, potential rating bias; Precision: moderate effect sizes observed; Consistent across studies; Interventions related to outcome of interest; Publication bias: not ascertained; Magnitude of effect: moderate; Dose response: N/A
Corcoran and Dattalo (2006)	Low/Moderate	Risk of bias: some lack of randomization (3/16); Precision: small effect sizes observed across studies; Consistent across studies; Behavioral therapies not found to be directly related to ADHD; Publication bias: large fail-safe N’s indicate low risk; Magnitude of effect: no or weak; Dose response: N/A
Cornell et al., (2018)	Moderate	Risk of bias: lack of randomization/blinding; Precision: moderate to large effect sizes observed; Consistent across studies; Interventions directly related to outcome; Publication bias: not ascertained; Magnitude of effect: fairly strong; Dose response: N/A
Fabiano et al., (2009)	High	Risk of bias: some lack of randomization; Precision: moderate to large effect sizes observed; Consistent across studies: Interventions directly related to outcome of interest; Publication bias: not ascertained; Magnitude of effect: fairly strong; Dose response: N/A
Fox et al., (2020)	Moderate	Risk of bias: reduced—only 1/14 studies used randomization/blinding, small samples reduce power of study; Precision: moderate effect sizes observed; Consistent across studies; Interventions directly related to outcome/population of interest; Publication bias: not ascertained; Magnitude of effect: moderate to strong; Dose response: N/A
Gaastra et al., (2016)	Moderate	Risk of bias: lack of randomization/blinding; Precision: large effect sizes observed; heterogeneity across studies; Interventions directly related to outcome of interests; Publication bias: funnel plot showed significant asymmetry, suggests underreporting of smaller studies showing no or small beneficial effects; Magnitude of effect: Strong; Dose response: N/A
Ghuman et al., (2008)	Low/Moderate	Risk of bias: some lack of randomization; Precision: moderate effect sizes observed; Inconsistencies across studies; Intervention not directly related to ADHD outcomes; Publication bias: not ascertained; Magnitude of effect: mild; Dose response: N/A
Groenman et al., (2022)	High	Risk of bias: Low risk of bias given RCTs used; Precision: Small to moderate effect sizes observed; Consistent findings reported across studies; Parenting interventions directly related to ADHD symptoms; Publication bias: not ascertained; Magnitude of effect: Small to medium; Dose response: N/A
Harrison et al., (2019)	Moderate	Risk of bias: single-case design, lack of randomization/blinding; Precision: moderate to large effect sizes observed; Consistent across multiple combination studies; Intervention related to outcome/population of interest; Publication bias: Egger’s test was non-significant indicating low risk of publication bias; Magnitude of effect: fairly strong; Dose response: N/A
Hodgson et al., (2014)	Moderate	Risk of bias: lack of randomization/blinding; Precision: moderate effect sizes observed; Inconsistencies across studies; Publication bias: not ascertained; Magnitude of effect: fairly strong; Dose response: no dose effect; Findings regarding confounding effect (no dose and age) increases GRADE
Hornstra et al., (2023)	High	Risk of bias: low risk of bias due to RCT designs; Precision: medium effect sizes, consistently across studies, range of CIs but mostly moderate CI on forest plot; Intervention directly related to outcome. Publication bias: Possible publication bias indicated through funnel plots, and egger’s test for behavioral problems and total ADHD symptoms. Effects adjusted for through trim-and-fill analyses; Magnitude of effect: Medium; Dose response: Higher dosage of “Shaping Knowledge” category, psychoeducation for parents led to smaller treatment effects on behavioral problems. Higher dosage of “negative consequences” associated with better treatment effects on behavioral problems
Iznardo et al., (2020)	Low/Moderate	Risk of bias: some lack of randomization; Precision: large effect sizes observed; Consistent across studies; Interventions directly related to outcome/population of interest; Publication bias: not ascertained; Magnitude of effect: fairly strong; Dose response: N/A
Krisanaprakornkit et al., (2010)	Moderate	Risk of bias: all RCTS, but small sample size reduces power of study; Precision: no to small effect sizes observed; Inconsistent across studies; Interventions not directly related to outcome of interest; Publication bias: not ascertained; Magnitude of effect: low; Dose response: N/A
Lee et al., (2012)	Moderate	Risk of bias: lack of randomization; Precision: small to large effect sizes (28/40 signified meaningful precision); BPT was consistently and directly related to outcome—ADHD, however, was not consistent in different groups, moreover, BPT effects declined during follow-up; Publication bias: not ascertained; Magnitude of effect: small to large; Dose response: N/A
McGoey et al., (2002)	Low/Moderate	Risk of bias: some lack of randomization, some methodological limitations, small sample sizes; Precision: mostly meaningful effect sizes observed across multiple combination studies (pharma/non-pharma/combined); Intervention related to outcome/population of interest, however relatively few studies examined treatment outcome for pre-school age children with ADHD; Publication bias: not ascertained; Magnitude of effect: mixed; Dose response: N/A
Mulqueen et al., (2015)	High	Risk of bias: low as all RCTs; Precision: large effect sizes observed; Consistent across studies; Interventions directly related to outcome; Publication bias: Egger’s test and funnel plot—significant amount of heterogeneity between trials but no evidence of publication bias; Magnitude of effect: strong; Dose response: N/A
Murray et al., (2018)	High	Risk of bias: low as all RCTs; Precision: large effect sizes observed across studies; Consistent across studies (9/11 large ES); Intervention directly related to outcome of interested; Publication bias: not ascertained; Magnitude of effect: mostly strong; Dose response: N/A
Pauli-Pott et al., (2021)	High	Risk of bias: low risk as all included studies were RCTs; Precision: moderate to large effect sizes, with significant heterogeneity. However, analyses heterogeneity explained by study quality, with higher quality studies having larger effect sizes (moderator analyses increase score). Intervention directly related to outcome. Magnitude of effect: moderate to large. Dose response: N/A
Pyle and Fabiano (2017)	Very Low	Risk of bias: High risk of bias given single case study design; Precision: Varied effect sizes from small to large, general similar pattern of results across studies; Intervention directly related to outcome; Publication bias: fail-safe N suggested publication bias is unlikely to distort findings; Magnitude of effect: Unclear; Dose response: N/A
Reid et al., (2005)	Moderate	Risk of bias: lack of randomization/blinding, small sample size reduces power of study; Precision: moderate to large effect sizes observed (19/27 calculated ES were moderate to large); Consistent across studies; Intervention directly related to outcome; Publication bias: not ascertained; Magnitude of effect: strong; Dose response: results from combined effect of SRI and medication increases the GRADE
Rimestad et al., (2019)	High	Risk of bias: low as all RCTs; Precision: moderate effect sizes observed; Consistent across studies (9/16 moderate ES, 6/16 small ES); Intervention directly related to outcome of interest; Publication bias: Egger’s test was non-significant indicating low risk of publication bias; Magnitude of effect: moderate; Dose response: N/A
Storebo et al., (2019)	High	Risk of bias: low as all RCTs; Precision: small to moderate effect sizes; Consistent across studies; Intervention directly related to outcome/population of interest; Publication bias: Egger’s test was non-significant and funnel plot was symmetrical, suggesting no publication bias; Magnitude of effect: Moderately strong; Dose response: N/A
Tan-McNeill et al., (2021)	Low	Risk of bias: High risk of bias as minority of included studies were RCTs (7/15). Precision: ES not ascertained. Heterogeneity of studies identified. Interventions directly related to outcome. Magnitude of effect: unclear. Dose response: N/A
Vacher et al., (2020)	Moderate	Risk of bias: some lack of randomization, small sample sizes reduce power; Precision: moderate; heterogeneity of outcome measures across studies; consistently related to ADHD outcomes; Publication bias: not ascertained; Magnitude of effect: fairly strong; Dose response: N/A
Van der Oord et al., (2008)	High	Risk of bias: low as all RCTs; Precision: large effect sizes observed across multiple combination studies (pharma/non-pharma/combined); Intervention directly related to outcome/population of interest; Publication bias: fail-safe N’s were substantial, low risk of bias; Magnitude of effect: mostly strong; Dose response: N/A
Vekety et al., (2021)	Moderate	Risk of bias: some lack of randomization/blinding; Precision: small to medium effect sizes observed; the overall effect was significant and moderate when the informants were teachers, but when parents or the children themselves rated their own behavior, the effects were non-significant; Publication bias: Egger’s regression test and funnel plot supported the absence of publication bias; Magnitude of effect: small to medium; Dose response: N/A
Wilkes-Gillan et al., (2021)	Low/Moderate	Risk of bias: some lack of randomization/blinding and confounding bias, but methodological quality mostly strong; Precision: findings from this review are preliminary in nature, medium to large effect sizes observed in two studies and one study reported large effect size, overall effect sizes not clearly reported; Publication bias: not ascertained; Magnitude of effect: moderate; Dose response: N/A
Willis et al., (2019)	Moderate	Risk of bias: some lack of randomization, small sample sizes reduce power; Precision: some meaningful precision; Consistent and directly related to ADHD outcomes; Publication bias: not ascertained; Magnitude of effect: fairly strong; Dose response: N/A
Zwi et al., (2011)	Moderate	Risk of bias: low as all RCTs; Precision: large effect sizes observed (9/11 studies); Consistent across studies; Intervention directly related to outcome of interest; Publication bias: not ascertained; Magnitude of effect: mostly strong; Dose response: N/A; Interventions effects maintained in 9 studies at 1-year follow-up
Anxiety		
Ale et al., (2015)	Moderate	Risk of bias: low due to RCTs; Precision: small effect sizes observed across studies; Inconsistent findings reported across studies; CBT directly related to anxiety; Publication bias: not ascertained; Magnitude of effect: Weak; Dose response: N/A
Bennet et al., (2013)	High	Risk of bias: low due to RCTs; Meaningful precision with large effect size; Consistent across studies; CBT directly related to anxiety; Publication bias: not ascertained; Magnitude of effect: Fairly strong; Dose response: N/A
Caldwell et al., (2019)	Moderate	Risk of bias: most studies RCTs, some non-randomized trials. However, most studies had unclear risk of bias for randomization and blinding; Precision: Small to moderate effect sizes observed; Consistent findings reported across studies; CBT directly reduced mood disorders (compared with waitlist); Publication bias: not ascertained; Magnitude of effect: Medium; Dose response: N/A
Comer et al., (2019)	Moderate	Risk of bias: majority of studies RCTs (20/38); Precision: no effect sizes reported, but interventions classified into evidence base levels; CBT directly related to outcome—Anxiety; Publication bias: not ascertained; Magnitude of effect: Unclear; Dose response: N/A
Fisak et al., (2011)	High	Risk of bias: Most studies were RCTs, some other mixed methods; Precision: small effect sizes with expected confidence intervals; some variability across studies, Prevention programs directly related to anxiety at post and 6 months follow-up; Publication bias: some publication bias reported via funnel plots, though corrected for with weighted effect sizes; Magnitude of effect: Small; Dose response: n.s. pos association between number of sessions and magnitude of effect
Grist et al., (2019)	High	Risk of bias: low due to RCTs; Precision: large effect sizes observed; Consistent findings reported across studies (compared to non-CBT/placebo/waitlist); CBT directly related to anxiety; Publication bias: possible publication bias reported due to slight asymmetry in funnel plot; Magnitude of effect: Mostly strong; Dose response: N/A
Howes Vallis et al., (2020)	High	Risk of bias: minority of studies were RCTs, with only 19/47 including a control group. Precision: large effect sizes with moderate heterogeneity; CBT directly related to Anxiety: Publication bias: funnel plots and Egger’s test analysis indicated publication bias present, adjusted estimates were similar to original analysis results; Magnitude of effect: large; Dose response: N/A
Krebs et al., (2018)	High	Risk of bias: low due to RCTs: Meaningful precision with small-moderate effect sizes; Consistent across studies; CBM-I directly related to anxiety: Publication bias: Egger’s test and funnel plots suggested some bias, but Duval-Tweedie analyses suggested no significant publication bias; Magnitude of effect: Moderately strong; Dose response: N/A
McGuire et al., (2015)	High	Risk of bias: low risk of bias for the RCTs; Precision: large effect sizes observed. Consistent across studies. CBT directly related to pop of interest. Publication bias: Small but ns publication bias found via Egger’s test and funnel plot; Mostly strong Magnitude of effect
Odgers et al., (2020)	Moderate	Risk of bias: low due to RCTs: small effect sizes; Inconsistent across studies; MBI directly related to anxiety only in one group of population; Publication bias: small asymmetry in funnel plots but non-significant on Egger’s test, results unlikely impacted by pub bias; Magnitude of effect: Weak and temporary; Dose response: N/A
Ostergaard et al., (2018)	Low/Moderate	Risk of bias: 3/15 studies included were RCTs, mixed designs otherwise; Precision: no overall effect size calculations due to heterogeneity across studies and small sample sizes; not directly related to outcome of interest; Publication bias: not ascertained; Magnitude of effect: Weak; Dose response: N/A
Phillips and Mychailyszyn (2021)	Low	Risk of bias: high risk as 5 of the 15 included studies did not have a control group; Precision: large effect size but precision impacted by small sample sizes and some notable differences in interventions across studies, Intervention directly related to outcome; Publication bias: not ascertained; Magnitude of effect: Large; Dose response: N/A
Reynolds et al., (2012)	High	Risk of bias: low due to RCTs: Meaningful precision with overall moderate effect sizes; Consistent across studies; CBT directly related to anxiety; Publication bias: assessed via funnel plots only, reported no evidence of bias; Magnitude of effect: Moderate; Dose response: N/A
Steains et al., (2021)	Moderate	Risk of bias: Low risk of bias as all included studies were RCTs; Precision: large effect size, impacted by small sample size as only included 5 studies with small samples, heterogeneity analyses n.s. Intervention directly related to outcome. Publication bias: no evidence of publication bias via fail-safe N, funnel plots, and trim and fill procedure. Magnitude of effect: large; Dose response: N/A
Viswanathan et al., (2022)	Moderate	Risk of bias: RCTS, some lack of blinding; Precision: moderate strength of evidence observed, only limited evidence available on long-term outcomes and on test accuracy and treatment in children; Consistent across studies; Publication bias: not ascertained; Dose response: N/A
Werner-Seidler et al., (2017)	Moderate	Risk of bias: low due to RCTs; Small effect sizes observed with small confidence intervals; Consistent findings reported across studies; Targeted School based intervention related to anxiety; Publication bias: some evidence of bias for depression studies via funnel plot and Egger’s test (effects were adjusted via Duval and Tweedie’s trim and fill procedure) and no evidence of bias for anxiety studies; Magnitude of effect: Weak; Dose response: N/A
Yin et al., (2021)	Moderate	Risk of bias due to RCTs; Precision: Small effect sizes; Inconsistent across different groups; Parent only CBT related to anxiety: Publication bias: assessed via Egger’s test, non-significant throughout; Magnitude of effect: Weak; Dose response: N/A
ASD		
Aldabas (2019)	Moderate	Risk of bias: high risk of bias given case series design. Precision: Large effect sizes observed, consistent findings reported across studies; Social stories directly related to ASD; Publication bias: not ascertained; Magnitude of effect: Large; Dose response: N/A
Camargo et al., (2014)	Very Low	Risk of bias: high risk of bias given single case designs, no control group, randomization or blinding; Precision: most studies reported similar direction of results, no ES reported; Intervention directly related to outcome; Publication bias: not ascertained; Magnitude of effect: Unclear; Dose response: N/A
Camargo et al., (2016)	Moderate	Risk of bias: high risk of bias given single case study design; Precision: moderate to large effect sizes observed; consistent across studies with expected confidence intervals; Behavioral interventions directly related to outcome of interest (ASD); Publication bias: not ascertained; Magnitude of effect: mostly strong; Dose response: N/A
Gunning et al., (2019)	Moderate	Risk of bias: high given single case study designs; Precision: no effect sizes reported, trends analyzed; SSI directly related to outcome of interest (ASD); Publication bias: not ascertained; Magnitude of effect: unclear; Dose response: N/A
Kokina and Kern (2010)	Moderate	Risk of bias: High risk of bias given single case study design; Precision: moderate to large effect sizes observed with large error margins; Social stories directly related to ASD; Publication bias: not ascertained; Magnitude of effect: low (given error margins); Dose–response: N/A
Reichow et al., (2013)	High	Risk of bias: low risk of bias for the RCTs; Precision: moderate effect sizes observed. Consistent across studies. SSG directly related to pop of interest. Publication bias: not ascertained (small number of studies precluded examination of funnel plot); Magnitude of effect: mostly moderate
Slaughter et al., (2020)	Moderate	Risk of bias: mixed designs, with methodology of evidence not clear given review of guidelines and websites; Precision: range of effect sizes observed (n.s. to large); CBI directly related to the pop of interest. Publication bias: not ascertained; Magnitude of effect: Mostly strong; Dose response: N/A
Tarver et al., (2019)	High	Risk of bias: low due to RCTs; Precision: small to moderate effect sizes observed; Consistent findings reported across studies; Behavioral parent intervention related to ASD; Publication bias: not ascertained due to insufficient number of studies; Magnitude of effect: Medium; Dose response: N/A
Vetter (2018)	Moderate	Risk of bias: moderate as most studies were single subject designs; Precision: unclear effect sizes; Direction of results mostly consistent across studies; PCIT directly related to outcome of interest (ASD); Publication bias: not ascertained; Magnitude of effect: unclear; Dose response: N/A
Wang et al., (2011)	Moderate	Risk of bias: high risk of bias due to single case study designs; Precision: large effect sizes observed in 12/14 studies with expected confidence intervals; Interventions directly related to ASD; Publication bias: not ascertained; Magnitude of effect: mostly strong; Dose–response: N/A
Wang et al., (2013)	High	Risk of bias: high risk of bias given single case study designs, Precision: large effect sizes observed; Consistent findings reported across studies with expected error margins; SSIs directly related to ASD; Publication bias: not ascertained; Magnitude of effect: strong; Research design found to be mediated the ES; Dose response: N/A. Findings on confounding increases GRADE
Wang and Spillane (2009)	Very Low	Risk of bias: High risk of bias as most studies were single case studies; Precision: wide range of ES from small to large even for the same intervention; Intervention directly related to outcome; Publication bias: not ascertained; Magnitude of effect: Unclear; Dose response: N/A
Weitlauf et al., (2017)	Moderate	Risk of bias: some lack of randomization/blinding; Precision: small effect sizes observed; Consistent findings reported across studies; limited evidence available to draw causality (intervention > ASD); Publication bias: not ascertained; Magnitude of effect: small; Dose response: N/A
Whalon et al., (2015)	High	Risk of bias: high risk of bias given single case study designs; Precision: moderate to strong effect sizes observed, with variable error margins; Consistent findings reported across studies; Interventions directly related to ASD; Publication bias: not ascertained; Magnitude of effect: Strong; Research design found to be mediated the ES; Dose response: N/A
Externalizing		
Bakker et al., (2017)	Moderate	Risk of bias: low due to RCTs; Precision: Small effect sizes observed; Consistent findings reported across studies; Psychosocial interventions directly related to outcome of interests; Publication bias: not determined; Magnitude of effect: Weak; Dose response: N/A; Comments on the quality of the included studies decrease GARDE
Barlow and Stewart-Brown (2000)	Moderate	Risk of bias: minority of studies (6/16) were RCTs, others non-randomized allocation; Precision: Moderate to large effect sizes observed in 5 studies (11/16 studies did not provide ES), small sample sizes reduces the power of the study; Consistent findings reported only across 5/16 studies; interventions directly related to pop of interest; Publication bias: not ascertained; Magnitude of effect: Mostly strong; Dose–response: N/A
Battagliese et al., (2015)	High	Risk of bias: low due to RCTs; Precision: Moderate to large effect sizes observed; Consistent findings reported across studies; CBT directly related to outcome of interests; Publication bias: not ascertained; Magnitude of effect: Medium; Dose response: N/A
Baumel et al., (2016)	High	Risk of bias: low due to RCTs; Precision: Moderate effect sizes observed; Consistent findings reported across studies; DPT directly related to outcome of interests; Publication bias: funnel plots indicated that there was no significant publication bias; Magnitude of effect: Medium but maintained after follow-up; Dose response: N/A
Baumel et al., (2017)	Moderate	Risk of bias: Most studies were RCTs, one non-randomized and one pre-post. Some studies reported minor influences on quality; Precision: moderate effect sizes observed; Consistent findings reported across studies; DPTs directly related to pop of interest; Publication bias: quality assessed via Cochrane tool, selection bias assessed to be low risk for all studies; Magnitude of effect: Mostly moderate; Dose–response: N/A
Burkey et al., (2018)	High	Risk of bias: low due to RCTs; Precision: Moderate effect sizes observed; Consistent findings reported across studies; Interventions directly related to outcome of interests; Publication bias: no pub bias suggested via funnel plots; Magnitude of effect: moderate; Dose response: N/A
Cai et al., (2022)	Moderate	Risk of bias; some lack of randomizations/blinding; Precision: small effect sizes observed with a small sample size; Within studies with at least one follow-up assessment(s), the trajectories of the intervention effects were inconsistent.; Publication bias: Egger’s test revealed no publication bias was evident; Magnitude of effect: small to moderate; Dose response: N/A
Comer et al., (2013)	High	Risk of bias: low due to RCTs; Precision: Large effect sizes observed; Consistent findings reported across studies; Interventions directly related to outcome of interests; Publication bias: trim and fill analysis via funnel plots did not suggest significant publication bias; Magnitude of effect: moderate; Dose response: N/A;
Connor et al., (2006)	Moderate	Risk of bias: psychotherapy studies were all RCTs; Precision: Moderate to large effect sizes observed; Consistent findings reported across studies; Interventions directly related to pop of interest; Publication bias: not ascertained; Magnitude of effect: Mostly strong; Dose–response: N/A
de Graaf et al., (2008)	High	Risk of bias: Most studies (14/15) RCTs; Precision: Large effect sizes observed; Consistent findings reported across studies; interventions directly related to pop of interest; Publication bias: not ascertained; Magnitude of effect: Mostly strong; Dose–response: N/A
Dedousis-Wallace et al., (2021)	High	Risk of bias: low due to RCTs; Precision: large effect sizes observed; Consistent findings reported across studies; Interventions directly related to outcome of interests; Publication bias: overall low risk of selection bias reported via Cochrane RoB tool; Magnitude of effect: Strong; Dose response: N/A
Dretzke et al., (2005)	High	Risk of bias: low due to RCTs; Precision: large effect sizes observed (27/37); Consistent findings reported across studies; Interventions directly related to outcome of interests; Publication bias: not ascertained; Magnitude of effect: Strong; Dose response: N/A
Dretzke et al., (2009)	High	Risk of bias: low due to RCTs; Precision: Moderate effect sizes observed (27/37); Consistent findings reported across studies; Interventions directly related to outcome of interests; Publication bias: Egger and Begg analyses revealed no evidence of publication bias; Magnitude of effect: Mostly medium; Dose response: N/A
Florean et al., (2020)	High	Risk of bias: low due to RCTs; Precision: Small to moderate effect sizes observed; Consistent findings reported across studies; Intervention directly related to outcome of interest; Publication bias: not ascertained; Magnitude of effect: Medium; Dose response: N/A
Forster et al., (2012)	Moderate	Risk of bias: mostly controlled trials, though randomization methods unclear. Precision: Moderate to large effect sizes observed within groups and between groups (treatment/control); Consistent findings reported across studies; interventions directly related to pop of interest; Publication bias: not ascertained; Magnitude of effect: Mostly strong; Dose–response: N/A
Fossum et al., (2008)	Moderate	Risk of bias: Most studies used randomization; Precision: Small effect sizes observed; Consistent findings reported across studies; Psychological interventions directly related to pop of interest; Publication bias: not ascertained; Magnitude of effect: Mostly moderate; Dose–response: N/A
Fossum et al., (2016)	Moderate	Risk of bias: Mixed design but unclear how many randomized; Precision: Moderate to large effect sizes observed; Consistent findings reported across studies; Psychological interventions directly related to pop of interest; Publication bias: not ascertained; Magnitude of effect: Mostly moderate; Dose–response: N/A
Furlong et al., (2012)	Moderate	Risk of bias: most studies (10/13) were RCTs; Precision: small to moderate observed; Consistent findings reported across studies, various sources of bias (though reported within the review); Parenting behavior and CBT interventions directly related to pop of interest; Publication bias: assessed through funnel plots, concluded publication bias unclear given heterogeneity across studies; Magnitude of effect: Mostly strong; Dose–response: N/A
Gardner et al., (2019a)	High	Risk of bias: low due to RCTs; Precision: Large effect sizes observed; Consistent findings reported across studies; IY Interventions directly related to outcome of interests; Publication bias: not ascertained; Magnitude of effect: Mostly strong; Dose response: N/A
Gardner et al., (2019b)	Low/Moderate	Risk of bias: low due to RCTs; Precision: Small effect sizes observed; Inconsistent findings reported across 2 meta-analyses; Interventions not directly related to outcome of interests; Publication bias: not ascertained; Magnitude of effect: minimum or no; Dose response: N/A
Lane et al., (2023)	Low	Risk of bias: high as all RCTS, but rated at unclear or high risk across most domains (mainly lack of blinding); Precision: evidence of very low certainty; Insufficient evidence to reach any firm conclusions regarding the effectiveness; Publication bias: not ascertained; Dose response: N/A
Leijten (2020)	High	Risk of bias: low due to RCTs; Precision: small to moderate effect sizes observed; Consistent findings reported across studies; Intervention reduced conduct problems; Publication bias: not ascertained, but reported that risk of bias was low on most indicators; Magnitude of effect: Medium; Dose response: N/A
Leijten et al., (2013)	Moderate	Risk of bias: low as most studies RCTs, few non-randomized trials; Precision: small effect sizes observed; Consistent findings reported across studies, but not maintained at follow-up (most studies only collected follow-up data in intervention studies); Interventions directly related to pop of interest; Publication bias: not ascertained; Magnitude of effect: Weak; Dose–response: N/A
Leijten et al., (2016)	High	Risk of bias: low due to RCTs; Precision: Significant effect sizes observed; Consistent findings reported across studies; Interventions directly related to outcome of interests; Publication bias: not ascertained; Magnitude of effect: Large; Dose response: N/A
Leijten et al., (2018)	High	Risk of bias: low risk of bias as these were two meta-analyses including only RCTs; Precision: 156 and 41 RCTs in the meta-analyses resulting in 386 effect sizes, with average effect size of the programs on disruptive child behavior d = − .47 (95% CI [− .55, − .40]). Consistency across studies. Publication bias: not ascertained; Magnitude of effect similar across studies. Follow-up times in studies typically about 1 year – longer term follow up was rare
Losel and Beelmann (2003)	High	Risk of bias: low due to RCTs; Precision: overall, small to moderate effect sizes observed; Consistent findings reported across studies; CBT Interventions directly related to outcome of interests; Publication bias: not ascertained; Magnitude of effect: Mostly medium; Dose response: N/A; Findings on confounder (age) increases GRADE
Maughan et al., (2005)	Moderate	Risk of bias: Some risk of bias, including RCTs and non-RCT with variability in study quality; Precision: Overall moderate to large effect sizes observed, effect size varied by study quality; Interventions directly related to pop of interest; Publication bias: not ascertained; Mostly moderate magnitude of effect; Dose–response: N/A;
Menting et al., (2013)	Moderate	Risk of bias: Low risk of bias due to mostly RCTs; Precision: Small effect sizes observed; Consistent findings reported across studies; IY Interventions directly related to outcome of interests; Publication bias: not ascertained; Magnitude of effect: Mostly weak; Dose response: N/A
Mingebach et al., (2018)	Moderate	Risk of bias: overall risk of bias rated as satisfactory in the paper, consists of meta-analyses; Precision: Moderate effect sizes observed, with risk of bias analyses within paper suggesting robust results; Consistent findings reported across studies; Parenting-based interventions directly related to pop of interest; Publication bias: risk of bias analyses from funnel plots and fail-safe Ns suggest some but small publication bias; Magnitude of effect: Moderate; Dose–response: N/A
Nogueira et al., (2022)	Moderate	Risk of bias: low risk of bias due to all RCTS, but some studies did not report randomization/blinding; Precision: small effect sizes (secondary outcomes) and moderate effect sizes (all GTP targeted outcomes); Interventions related to outcome; Publication bias: not ascertained; Magnitude of effect: mostly moderate; Dose response: N/A
Nye (2019)	High	Risk of bias: low due to RCTs; Precision: Moderate effect sizes observed; Consistent findings reported across studies; Intervention directly related to outcome of interest; Publication bias: not ascertained due to small number of studies; Magnitude of effect: Medium; Dose response: N/A
Parker et al., (2021a, 2021b)	Moderate	Risk of bias: most studies used randomized treatment (20/32) and 24 studies (24/32) used treatment protocols; Precision: small to moderate effect sizes observed with a large sample size; Publication bias: not ascertained; Magnitude of effect: moderate; Dose response: N/A
Riise et al., (2021)	High	Risk of bias: most studies RCT, some open trials without randomization and blinding, overall low risk of bias in paper’s risk of bias calculations; Precision: large effect sizes observed with precise CIs; Consistent findings reported across studies; Interventions directly related to pop of interest; Publication bias: trim-and-fill method &Egger’s test indicated that publication bias is likely an issue for the primary continuous measure studies and have inflated the effect size; Magnitude of effect: mostly strong; Dose–response: N/A;
Smith et al., (2021)	Moderate	Risk of bias: all RCTS but high risk of detection bias (lack of blinding of outcome assessment); Precision: small to moderate effect sizes; Publication bias: not ascertained but strong possibility of publication bias; Magnitude of effect: small; Dose response: N/A
Solomon et al., (2017)	Moderate	Risk of bias: Some risk of bias due to half of studies (7/15) lacking randomization; Precision: small to moderate effect sizes observed with sometimes large CIs; Consistent findings reported across studies; Interventions directly related to pop of interest; Publication bias: not ascertained; Mostly moderate magnitude of effect; Dose–response: N/A
Stoltz et al., (2012)	High	Risk of bias: Most studies included were RCTs (73%), otherwise non-randomized trials; Precision: Moderate effect sizes observed, with some variability in confidence intervals; Mostly consistent findings reported across studies; Interventions directly related to pop of interest; Publication bias: not ascertained; Magnitude of effect: Moderate; Dose–response: N/A;
Tarver et al., (2014)	High	Risk of bias: low due to RCTs; Precision: Moderate to large effect sizes observed; Consistent findings reported across studies; Interventions directly related to outcome of interests; Publication bias: not ascertained; Magnitude of effect: Mostly strong; Dose response: N/A
Thongseiratch et al., (2020)	High	Risk of bias: low due to RCTs; Precision: Small to moderate effect sizes observed; Consistent findings reported across studies; Intervention directly related to outcome of interest; Publication bias: not ascertained; Magnitude of effect: Medium; Dose response: N/A
Tse, 2006	Moderate	Risk of bias: Some risk of bias due to only 1/5 studies being an RCT; Precision: Overall small to moderate effect sizes observed; Mixed findings (some n.s.) reported across studies; impacted by small sample sizes; Interventions directly related to pop of interest; Publication bias: not ascertained; Mostly moderate magnitude of effect; Dose–response: N/A
Tully and Hunt (2016)	High	Risk of bias: low due to RCTs; Precision: overall, moderate effect sizes observed; Consistent findings reported across studies; Interventions directly related to outcome of interests; Publication bias: not ascertained; Magnitude of effect: Mostly medium; Dose response: N/A;
Uretsky and Hoffman (2017)	Moderate	Risk of bias: most studies were RCTs, some non-randomized and some single-group; Precision: Small to moderate effect sizes observed with varying effect sizes; Overall consistent findings reported across studies; Interventions directly related to pop of interest; Publication bias: not quantitatively ascertained, suggested potential publication bias due to heterogeneity among studies; Magnitude of effect: Moderate; Dose–response: N/A
Veenman et al., (2018)	Moderate	Risk of bias: low due to RCTs; Precision: overall, small to moderate effect sizes observed; Consistent findings reported across studies; Interventions directly related to outcome of interests; Publication bias: fail-safe N analyses found no evidence of pub bias; Magnitude of effect: Medium; Dose response: N/A;
Ward et al., (2016)	Moderate	Risk of bias: Half of studies were RCTs, half without randomization or blinding; Precision: Large effect sizes observed with expected CIs; Consistent findings reported across studies; PCIT directly related to pop of interest; Publication bias: not ascertained; Magnitude of effect: Mostly strong; Dose–response: N/A
Ye et al., (2021)	High	Risk of bias: Includes RCTs and non-randomized controlled trials, risk of bias analyses reported half studies had randomizing and most studies had high risk of bias for blinding; Precision: large effect sizes observed, with varying error margins, Consistent findings reported across studies; interventions directly related to pop of interest; Publication bias: slight asymmetry in funnel plot for aggressive behavior but overall reported low risk of bias; Magnitude of effect: Mostly strong;
Internalizing		
Benarous et al., (2017)	Low	Risk of bias: most studies not RCTs, lack of randomization and blinding in studies; small sample size reduces the power for the study, no reported effect sizes; Consistent and directly related to ADHD outcomes; Publication bias: not ascertained; Magnitude of effect: moderate; Dose response: N/A
Caldwell (2019)	Moderate	Risk of bias: most studies RCTs, some non-randomized trials. However, most studies had unclear risk of bias for randomization and blinding; Precision: Small to moderate effect sizes observed; Consistent findings reported across studies; CBT directly reduced mood disorders (compared with waitlist); Publication bias: not ascertained; Magnitude of effect: Medium; Dose response: N/A
Cuijpers et al., (2023)	High	Risk of bias: Low risk of bias as all studies were RCTs; Precision: No ES reported, examined response rates, relative risks, and numbers-needed-to-be-treated. Response rates had expected confidence intervals. Consistent findings reported across studies; Interventions investigated directly related to outcome. Publication bias: evidence of publication bias, subgroup analyses conducted adjusting for publication bias; Magnitude of effect: Not assessed; Dose response: N/A
Forti-Buratti et al., (2016)	Low	Risk of bias: all RCTs but some methodological issues in each study, consistently low power, some studies without blinding, some studies with no appropriate control intervention (CBT + meds vs CBT); Precision: poor or no effect sizes observed. Small sample sizes. Consistently not related to outcome across studies; Publication bias: not ascertained; Mostly non-significant Magnitude of effect. Dose response: N/A
Michael and Crowley (2002)	High	Risk of bias: most were randomized controlled studies, but some studies without randomization and blinding; Precision: moderate effect sizes observed between groups (children/adolescents), with results reported as a function of study quality; Consistent across studies, esp higher quality studies; CBT Interventions directly related to outcome of interest; Publication bias: not ascertained; Magnitude of effect: Moderate; Dose response: N/A
Sun et al., (2019)	High	Risk of bias: low as all RCTs; Precision: large effect sizes observed. Consistently across studies; CBT directly related to outcome; Publication bias: potential publication bias assessed via egger’s weighted regression test, though trim and fill method suggested that this bias had minimal impact on results; Mostly strong Magnitude of effect. Dose response: N/A
Werner-Seidler et al., (2017)	High	Risk of bias: low risk of bias for the RCTs; Precision: small effect sizes observed. Consistent across studies. Interventions directly related to pop of interest; Publication bias: funnel plots estimated some publication bias, effects subsequently adjusted using trim and fill procedure. No evidence of bias for anxiety studies; Mostly small magnitude of effect, however, results on posttreatment effect increases the GRADE; Dose response: N/A
Yap et al., (2016)	Moderate	Risk of bias: low as all RCTs; Precision: Small effect sizes observed. Consistently across studies; PI directly related to outcome; Publication bias: no apparent publication bias via Egger’s tests; Mostly weak Magnitude of effect. Dose response: N/A
Mental health		
Bauer et al., (2021)	Low	Risk of bias: lacking randomization/blinding. Precision: effect sizes not ascertained; Social support consistently found to be unrelated to children, only one study referred to social support as mobilized by children directly; Publication bias: not ascertained; Magnitude of effect: weak; Dose response: N/A
Bayer et al., 2009	High	Risk of bias: low as all RCTs; Precision: large effect sizes observed; Consistent across studies; Interventions directly related to outcome; Publication bias: not ascertained; Magnitude of effect: mostly strong; Dose response: N/A
Benoit & Gabola, 2021	Low	Risk of bias: high risk of bias as study online included quasi-experimental or pre-post designs; Precision: large range of effect sizes, no overall effect size calculated given small sample size, Interventions directly related to outcome (child wellbeing). Publication bias: not ascertained. Magnitude of effect: unclear. Dose response: N/A
Blewitt et al., (2021)	Low/Moderate	Risk of bias: some lack of randomization/blinding; Precision: due to heterogeneity in study designs and outcome measures, global effect sizes were not calculated; Within and across studies were inconsistent; Publication bias: not ascertained; Magnitude of effect: high only in a few, mostly were limited due to insufficient data; Dose response: N/A
Bratton and et al., (2005)	Moderate	Risk of bias: lack of randomization/blinding; Precision: large effect sizes observed; Consistent across studies; Interventions directly related to outcome; Publication bias: not ascertained; Magnitude of effect: Strong; Dose response: N/A
Buchanan-Pascall et al., (2018)	High	Risk of bias: low as all RCTs; Precision: small to moderate effect sizes observed; Consistent across studies; Interventions directly related to outcome; Publication bias: not ascertained; Magnitude of effect: mostly small; Dose response: N/A; ES moderated by the study quality increases GRADE
Carr et al., (2017)	Low/Moderate	Risk of bias: some lack of randomization, some studies with methodological limitations—most underpowered; 6/17 RCTs; Precision: moderate to large effect sizes observed; Consistent across studies; Interventions directly related to outcome; Publication bias: not ascertained; Magnitude of effect: mostly strong; Dose response: N/A
Dalgaard et al., (2022)	Moderate	Risk of bias: majority of studies were RCTs, though there were some concerns reported for majority of RCTs and there were some non-randomized studies. Precision: most studies reported similar direction of results, small effect sizes reported with moderate to large confidence intervals; Intervention directly related to outcome; Publication bias: study reported that the limited number of studies does not permit definitive conclusions regarding publication bias; Magnitude of effect: small; Dose response: N/A
England-Mason et al., (2023)	High	Risk of bias: all RCTS of moderate quality; Precision: small to medium effect sizes observed in children outcome, but small sample size; Consistent across studies; Intervention directly related to outcome; Publication bias: trim and fill procedure did not indicate publication bias; Magnitude of effect: moderate; Dose response: N/A
Everett et al., (2021)	Moderate	Risk of bias: all RCTs, low risk of bias; Precision: No ES reported, precision unclear; broad scope but intervention directly related to outcome; Publication bias: not ascertained; Magnitude of effect: unclear; Dose response: N/A
Jugovac et al., (2022)	High	Risk of bias: Most studies RCTs (40/43), some non-randomized trials. Most studies had an unclear risk of bias for blinding. However, analyses were conducted to moderate for risk of bias. When only examining low risk studies, effect size increased for externalizing disorders. Precision: Small to moderate effect sizes observed; Intervention directly related to outcome; Publication bias: Funnel plots did not indicate publication bias.; Magnitude of effect: Small to medium.; Dose response: N/A
Law et al., (2012)	Very Low	Risk of bias: High risk of bias as most studies were single case designs. Precision: No ES calculated, though similar pattern of results across studies; Intervention directly targeted outcome; Publication bias: not ascertained; Magnitude of effect: Unclear; Dose response: N/A
Ledford et al., (2023)	Low	Risk of bias: high risk of bias given no control groups, Precision: large effect sizes reported with moderate confidence intervals, large heterogeneity across studies. Publication bias: not ascertained. Magnitude of effect: large overall but inconsistent. Dose response: N/A
McDonald and Drey (2018)	Low/Moderate	Risk of bias: some lack of randomization/blinding, limitations in methodology (sparse methodology in one study); Precision: moderate effect sizes observed; Heterogeneity of study populations and outcome measures was substantial; Publication bias: funnel plot suggested no evidence of publication bias; Magnitude of effect: medium; Dose response: N/A
Money et al., (2021)	Low	Risk of bias: high risk as only 2/6 of the included studies were RCTs; Precision: half of the interventions did not report ES, small samples and heterogeneity between included studies with no overall ES calculated, Intervention directly related to outcome, Publication bias: not ascertained. Magnitude of effect: unclear; Dose response: N/A
Moula, (2020)	Low	Risk of bias: high or unclear due to mainly selection bias, lack of randomization/blinding; Precision: moderate effect sizes observed; Consistent across studies; Interventions directly related to outcome; Publication bias: not ascertained; Magnitude of effect: moderate; Dose response: N/A; No information regarding loss to follow-up
Moula et al., (2020)	Moderate	Risk of bias: some lack of randomization/blinding, small sample sizes reduce power; Precision: moderate effect sizes observed; Consistent across studies; Interventions directly related to outcome; Publication bias: not ascertained; Magnitude of effect: mostly moderate; Dose response: N/A
Pester et al., (2019)	Very Low	Risk of bias: case series methodology, lacking randomization/blinding, low sample sizes reduces power; Precision: moderate effect sizes observed; Consistent across studies; Interventions directly related to outcome of interest; Publication bias: examination of funnel plot indicated potential underreporting of studies with larger effects for externalizing symptoms and smaller effects for internalizing symptoms; Magnitude of effect: moderate; Dose response: N/A
Pilling et al., (2020)	Moderate	Risk of bias: all RCTs, but moderate to high risk of bias most studies; Precision: small to moderate effect sizes observed; High levels of heterogeneity; Interventions directly related to outcome; Publication bias: asymmetric funnel plot and significant Egger’s test indicated presence of publication bias; Magnitude of effect: mostly moderate; Dose response: N/A
Sanchez et al., (2018)	High	Risk of bias: Low risk of bias given all studies were RCTs; Precision: Small to moderate effect sizes observed; Consistent findings reported across studies; Intervention type directly related to outcome; Publication bias: sensitivity analyses indicated that publication bias would not have influenced the interpretation of results; Magnitude of effect: Small to medium; Dose response: Service intensity moderated results (conducted more often led to greater effects)
Savaglio et al., (2023)	Moderate	Risk of bias: some lack of randomization (but 88% of studies assessed as having medium or high methodological quality); Precision: small to moderate effect sizes observed; Consistent findings reported across studies; Publication bias: assessed by Egger’s test and visual inspection of funnel plot, limited indication of publication bias; Magnitude of effect: moderate; Dose response: N/A
Schleider (2017)	High	Risk of bias: low as all RCTs; Precision: small to moderate effect sizes observed; Consistent across studies; Interventions directly related to outcome; Publication bias: slope of Egger regression line was non-significant, funnel plot indicated some asymmetry but potential for systematic bias was low; Magnitude of effect: mostly moderate; Dose response: N/A
Sheridan et al., (2019)	Moderate	Risk of bias: lack of randomization/blinding, rigor/quality of included group designs not considered; Precision: moderate effect sizes observed; Consistent across studies; Interventions directly related to outcomes of interest; Publication bias: not ascertained; Magnitude of effect: moderate; Dose response: N/A
Shucksmith et al., (2010)	Moderate/High	Risk of bias: low as all RCTs; Precision: moderate effect sizes observed; Consistent across studies; Publication bias: not ascertained; Magnitude of effect: mostly small; Dose response: N/A
Sprung et al., (2015)	Moderate	Risk of bias: lack of randomization/blinding; Precision: moderate effect sizes observed; Consistent across studies; Interventions directly related to outcome; Publication bias: fail-safe N & funnel plot indicate low potential; Magnitude of effect: Mostly moderate; Findings on confounders increases GRADE; Dose response: N/A
Sun et al., (2021)	Low/Moderate	Risk of bias: some lack of randomization, quality appraisal indicated significant variability in risk of bias across studies; Precision: quantitative analysis was not conducted, limited by the diversity of measurement tools in each outcome; 13/16 studies reported improvements in one outcome domain, heterogeneity of outcome measurements hindered comparison; Publication bias: not ascertained; Dose response: N/A
Zarakoviti et al., (2021)	Moderate	Risk of bias: low as all RCTs, most of which the study quality were deemed moderate to strong; Precision: effect size not reported for all 12 qualitative papers; 7/12 studies found significant reductions in internalizing symptoms, comorbid internalizing symptoms were less consistent; Publication Bias: not ascertained; Magnitude of effect: moderate; Dose response: N/A;

### Data Synthesis

Findings were tabulated as a function of mental health problem (Table 1). Given the heterogeneity of interventions and outcomes, an overall quantitative synthesis was determined to be not possible or valid. A narrative synthesis was then undertaken, with findings synthesized according to the mental health problem and intervention type. The size of treatment effects for specific problems or interventions were described when available (small, moderate, large).

## Results

**Fig. 1:**
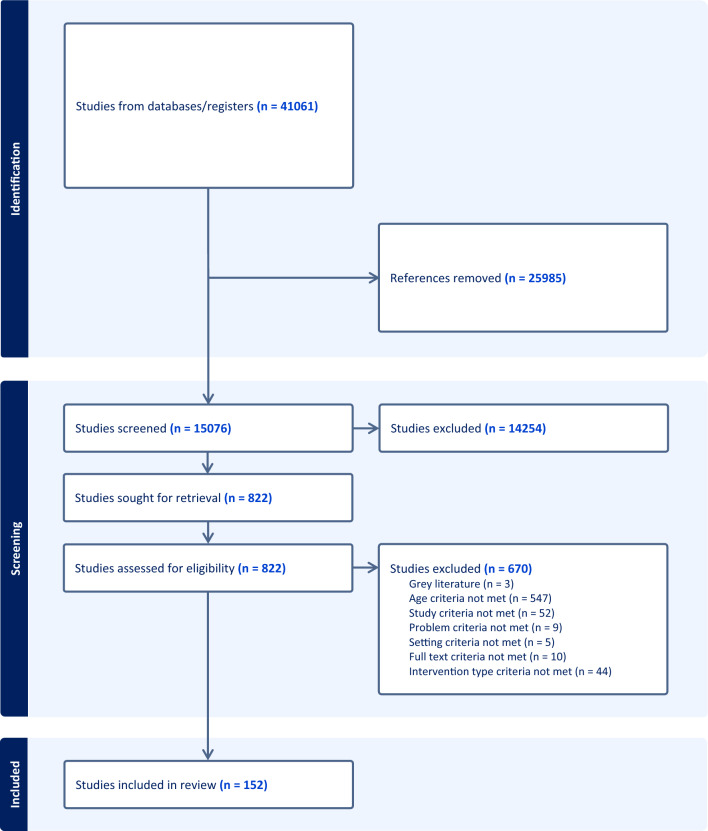
PRISMA flow chart

A total of 41,061 abstracts were retrieved, and of these 152 articles met the inclusion criteria (Fig. 1). The findings were evaluated according to interventions targeting the following: broad mental health needs, internalizing symptoms, externalizing symptoms, anxiety, depression, trauma, ADHD and ASD. Reviews within each symptom domain were categorized based on the types of interventions evaluated. Of the 152 included reviews, 48 received a high GRADE, 2 received a moderate/high GRADE, 57 received a moderate GRADE, 7 received a low to moderate GRADE, 25 received a low GRADE, and 13 received a very low GRADE (see Table 2) for GRADE score reasonings.

### Characteristics of Included Studies

Most reviews (*κ* = 101) were meta-analyses, whilst the remaining 51 were systematic reviews. The total number of studies included in the 101 meta-analyses varied (range = 5 to 197 studies). The total participant sample size was variable (range = 12 to 56,620 participants per review; although 6 meta-analyses did not report sample size), as was the age range (range = 0 to 32 years).^1^ The number of studies included in the 51 systematic reviews was also variable (range = 2 to 180 studies). The total number of participants ranged from 55 to 5,759 (12 did not report the total number). Within the systematic reviews, the age range varied from 0 to 21 years (Table 1).

### Summary of Evidence by Intervention Type

#### Interventions for Mental Health Symptoms

A total of 28 reviews of interventions for a broad range of mental health symptoms were identified. These 28 reviews largely represented interventions aimed at improving various broad mental health symptoms, including emotional, social, and behavioral symptoms. Despite that, sometimes measures of specific symptoms were also included (e.g., depression) and we have reported these findings alongside those for broad mental health symptoms.

##### Mixed Psychosocial Interventions for Mental Health Symptoms

Three meta-analyses examined the efficacy of mixed psychosocial interventions in reducing a constellation of mental health problems (emotional, behavioral, social) in children (Pilling et al., 2020; Sanchez et al., 2018; Schleider & Weisz, 2017), with significant small to moderate pooled effect sizes reported. These papers were rated as being of moderate (Pilling et al., 2020) and high quality (Sanchez et al., 2018; Schleider & Weisz, 2017). A large scale meta-analysis conducted by Pilling et al., (2020) found that psychological interventions overall (including a range of treatments like CBT, psychoeducation, and behavioral-based parenting training) conducted in a range of clinical, community, and school settings lead to moderate effects on improving mental health symptoms in children, with effects retained at 12-month follow-up. Similarly, Schleider and Weisz (2017) highlighted in their meta-analysis that single session psychosocial interventions were efficacious for treating some mental health problems, specifically anxiety and conduct problems in young children in mostly clinical settings; though effects were not retained at 13-week follow-up. Behavioral interventions demonstrated a large effect, whereas non-behavioral interventions (e.g., attention bias modification, “growth mindset”) showed small effects. Both meta-analyses showed less (smaller effect sizes) or no (non-significant) efficacy for the use of these mixed psychosocial programs in the treatment of depressive symptoms (Pilling et al., 2020; Schleider & Weisz, 2017). Lastly, Sanchez et al. (2018) reported in their meta-analysis that school-based generic mental health programs similarly were associated with small to medium effect sizes with larger effect sizes for externalizing symptoms (medium effect sizes) compared to internalizing symptoms and attention problems. Taken together, these three moderate to high quality reviews suggest that generic psychosocial interventions overall are efficacious for child mental health symptoms, with smaller effects for depression and internalizing difficulties.

Regarding moderators, the meta-analyses found that younger children benefited more than older children from single-session interventions (Schleider & Weisz, 2017), and tended to do better following psychological interventions at follow up (Pilling et al., 2020). Pilling et al. (2020) emphasized that interventions were generally as effective in school as other settings when outcomes were compared 1-year after the intervention. Furthermore, the efficacy of anxiety and depression interventions (at 1-year follow-up) was similar when conducted by paraprofessionals or professionals. However, interventions led by paraprofessionals were less effective for treatment of conduct problems when compared to interventions led by professionals, and group programs were associated with negative 1-year outcomes. Beyond this, Sanchez et al. (2018) found that targeted intervention and selective prevention programs led to high-medium to large effects (and these were larger than the small effects observed following universal prevention).

##### Behavioral-Based Parenting Interventions for Mental Health Symptoms

A total of four meta-analyses (Buchanan-Pascall et al., 2018; Carr et al., 2017; Savaglio et al., 2023; Sheridan et al., 2019), and four systematic reviews (Bayer et al., 2009; Everett et al., 2021; Shucksmith et al., 2010; Zarakoviti et al., 2021). evaluated parenting interventions for a range of mental health symptoms in children. Most interventions were rated of moderate to high quality, except for Carr et al. (2017), which was rated as low to moderate quality. A meta-analysis by Savaglio et al., (2023) found the largest evidence base for parenting-focused programs for internalizing and/or externalizing disorders. Furthermore, a systematic review by Everett et al. (2021) denoted those interventions that targeted parenting behavior led to improvements in both child outcomes, as well as parental psychopathology and parental behavior.

Regarding intervention types, one systematic review concluded that the four parenting programs that were considered effective for managing behavioral problems for school-aged children include the Good Behavior Game, Incredible Years, John Hopkins Prevention Program and Parenting Through Change Program (Bayer et al., 2009). For pre-school aged children’s behavioral problems, Incredible Years, Triple P, and the US Family Check-up were found to be the most efficacious. Subsequently, regarding emotional problems, Bayer et al. (2009) found that The Parent Education Program and The Brief Psycho-educational Group-Based Program were the most efficacious for pre-school aged children, and Fast Track for school-aged children.

Four reviews, including three meta-analyses and one systematic review, found that parenting and family-based programs significantly reduced internalizing and externalizing problems in both clinical and community settings (Buchanan-Pascall et al., 2018; Carr et al., 2017; Savaglio et al., 2023; Zarakoviti et al., 2021). The benefits of family-based group behavioral interventions extended to school settings, with two studies showing efficacy for improving social-behavioral competence (e.g., prosocial skills, peer-relationships, self-regulation, externalizing problems) and mental health symptoms (Sheridan et al., 2019; Shucksmith et al., 2010). However, irrespective of setting, there were overall fewer studies but also smaller effect-sizes, or non-significant findings in managing internalizing symptoms compared to externalizing symptoms (Bayer et al., 2009; Buchanan-Pascall et al., 2018; Sheridan et al., 2019).

Consistent moderators of efficacy were identified across systematic reviews conducted in clinical or community settings, including stronger effects for families with children with less severe problems and for externalizing problems (Buchanan-Pascall et al., 2018; Carr et al., 2017; England-Mason et al., 2023). Regarding other moderators, while Carr et al. (2017) highlighted stronger effects for younger children in clinical and community settings, Sheridan et al. (2019) showed no differential age effects in school-based settings. Mixed findings were also found for duration of treatment, with some showing that longer treatments were more effective (Carr et al., 2017) and others highlighting that number of session hours did not impact outcome (Buchanan-Pascall et al., 2018).

##### Child-Centered Play Therapy for Mental Health Symptoms

Four reviews examined mental health and related outcomes following child-centered play-based therapy, with one meta-analysis reviewing case-studies (Pester et al., 2019), one meta-analysis evaluating a range of controlled trials (Bratton et al., 2005), and one systematic review and one meta-analysis evaluating mixed methods designs (Ledford et al., 2023; Money et al., 2021). Studies on play therapy were rated as very low to low/moderate in quality. Two meta-analyses reported small to large effect sizes of child-centered play therapy for various mental health outcomes (Bratton et al., 2005; Pester et al., 2019) and social skills (Ledford et al., 2023). Consistently, play therapy led to improvements in internalizing and externalizing symptoms (Bratton, 2005; Money et al., 2021; Pester et al., 2019). Pester et al. (2019) also found small to moderate effect-sizes for social skills, but play therapy was not effective for improving self-regulation skills.

##### Socio-Emotional Interventions for Mental Health Symptoms

A total of five systematic reviews and three meta-analyses (Bauer et al., 2021; Blewitt et al., 2021; Dalgaard et al., 2022; England-Mason et al., 2023; Jugovac et al., 2022; Law et al., 2012; Sprung et al., 2015; Sun et al., 2021) investigated the effect of interventions targeting socio-emotional aspects of child mental health, with mixed evidence. One moderate quality meta-analysis examined the effects of programs delivered in a range of settings (including outpatient clinics and school settings) focusing on improving children’s understanding of emotions (recognizing, understanding and reflecting upon emotions) (Sprung et al., 2015). These researchers reported small to moderate effect-sizes in improving emotional competence across these three domains, with longer treatments associated with stronger effects. Furthermore, a recent high quality meta-analysis by England-Mason et al. (2023) found that parenting interventions that focused on emotion socialization were also effective for improving aspects of internalizing and externalizing symptoms, including child emotional competence and behavioral adjustment.

Two studies investigated attachment-based intervention programs. One high quality meta-analysis by Jugovac et al. (2022) found that attachment and emotion-focused parenting interventions led to improvements in internalizing and externalizing disorders, with larger effects for internalizing disorders. However, this result is not consistent across studies, as a systematic review by Dalgaard et al. (2022) found that attachment-based interventions led to a slightly greater effect for externalizing disorders rather than internalizing disorders for children with foster and adoptive parents.

Beyond this, two systematic reviews evaluating social and emotional learning programs found that they led to improvements in various social-emotional outcomes (Blewitt et al., 2021; Sun et al., 2021), though the quality of these studies were low to moderate. Blewitt et al. (2021) found that social and emotional learning programs overall improved social competence but had mixed evidence on behavioral regulation and led to non-significant differences in emotional competence. Sun et al. (2021) evaluated yoga and mindfulness-based interventions on social-emotional learning and found positive results in behavioral regulation, emotion regulation, and social skills. There was evidence for three other programs targeting social support, social skills, and communication skills specifically, but quality for these papers were low (Bauer et al., 2021) and very low (Law et al., 2012). Bauer et al. (2021) found that interventions aimed at mobilizing social support led to improvements in child behavior, cognitive and social development outcomes, coping, and psychological functioning, with small effect sizes. Law et al. (2012) also found overall positive results for behavioral interventions targeting communication difficulties, but results are limited by study quality.

Regarding potential moderators, Sprung et al. (2015) found that whereas improvements in external emotional competency were more often found when the program was delivered in group settings, improvements in reflective emotional understanding were more likely found for individually delivered programs. Environmental setting (e.g., classroom, area in school, lab) also moderated results. Children with lower baseline social-emotional functioning also demonstrated greater improvements (Sun et al., 2021).

##### Art Therapy for Mental Health Symptoms

Three systematic reviews examined art-based therapies conducted with primary age children (5 to 12 years) in school settings, reporting some small but significant effects on some mental health outcomes (McDonald & Drey, 2018; Moula, 2020; Moula et al., 2020). One review reported significant positive improvements in reducing defiant behavior and separation anxiety symptoms but not for locus of control (McDonald & Drey, 2018). Two other reviews showed significant improvements in self-esteem and aggression but small changes in depression, anxiety, attention and withdrawal (Moula, 2020; Moula et al., 2020). However, these results are provisional due to a small number of trials included in these reviews and the low to moderate quality of these studies.

##### Positive Psychology Interventions for Mental Health Symptoms

One systematic review investigated the effect of positive psychology interventions on broad child mental health symptoms (Benoit & Gabola, 2021). Positive psychology interventions were shown to have mixed benefits on child wellbeing, including non-significant or positive results for change in positive emotions and engagement and improvements in prosocial behavior but non-significant changes in teacher–child relationships. However, positive psychology interventions did show benefits on quality of life and life satisfaction in two studies. Importantly, conclusions are limited due to the small number of studies meeting inclusion criteria (*n* = 3) and the subsequent low-quality appraisal of this review.

#### Interventions for Children with Internalizing Symptoms

We identified two meta-analyses which evaluated the efficacy of interventions in managing internalizing symptoms in children. These two meta-analyses are discussed below.

##### Mixed Psychosocial Interventions for Internalizing Symptoms in Children

One meta-analysis of moderate quality evaluated the efficacy of a range of psychosocial interventions in managing internalizing symptoms in children, with small effect sizes at post-intervention and follow-up reported (Yap et al., 2016). A significantly better, albeit small effect size was found for selective relative to universal interventions.

##### Behavioral, Cognitive, and Cognitive Behavioral Interventions for Internalizing Symptoms in Children

One high quality meta-analysis evaluated the efficacy of CBT interventions in reducing internalizing symptoms in children and reported a large, within-group effect size post-therapy and at follow-up (Sun et al., 2019). Interventions which included parental involvement contributed to a significantly larger effect size, whereas age, treatment mode (individual vs. group), goal setting or length did not moderate treatment efficacy.

#### Interventions for Children with Externalizing Symptoms

We identified 44 reviews which evaluated the efficacy of interventions in managing externalizing symptoms only in children. These reviews are discussed below.

##### Mixed Psychosocial Interventions for Externalizing Symptoms in Children

A total of 11 reviews (seven meta-analyses and four systematic reviews) of mostly moderate to high quality examined mixed psychosocial interventions for externalising symptoms (Bakker et al., 2017; Barlow & Stewart-Brown, 2000; Battagliese et al., 2015; Burkey et al., 2018; Comer et al., 2013; Connor et al., 2006; Fossum et al., 2008, 2016; Lane et al., 2023; Stoltz et al., 2012; Tse, 2006). Of these studies, quality was lower for Lane (2023) and Tse (2006), which were rated low. Meta-analyses showed that behavioral-based interventions had greater efficacy than non-behavioral-based interventions (Comer et al., 2013; Fossum et al., 2008, 2016). However, meta-analyses which examined interventions that included behavioral and non-behavioral elements still revealed small to moderate effect-sizes for externalizing symptoms (Bakker et al., 2017). Beyond this, one meta-analysis also found that personalized interventions led to a slightly greater improvement in child conduct problems compared to non-personalized interventions, as measured through the ECBI Problem Subscale in the short term, but not for other outcome measures (Lane et al., 2023). However, conclusions are limited as the study was considered low quality. Three systematic reviews evaluated the efficacy of a mixed array of psychosocial interventions for managing externalizing symptoms in children (Barlow & Stewart-Brown, 2000; Connor et al., 2006; Tse, 2006). Collectively, the findings from these reviews supported the small to moderate effect sizes documented in the meta-analyses.

There were inconsistent findings in the reviews about the moderating impact of age (Burkey et al., 2018; Comer et al., 2013; Fossum et al., 2016) and the involvement of children on outcome (Battagliese et al., 2015; Comer et al., 2013). Individual, compared to group interventions demonstrated greater reductions in conduct problems in one review (Fossum et al., 2016) but not in another  (Comer et al., 2013). For young children, individual psychosocial interventions delivered at school were more beneficial for reducing disruptive behavior when combined with additional classroom and/or school-wide interventions (Stoltz et al., 2012).

##### Behavioral-Based Parenting Interventions for Externalizing Symptoms in Children

For externalising disorder interventions, parenting treatments had the strongest evidence. We identified 22 meta-analyses, three systematic reviews, and one meta-meta-analysis, that evaluated behavioral-based parenting interventions. Commonly evaluated interventions included: Incredible Years (Forster et al., 2012; Furlong et al., 2012; Gardner et al., 2019a, 2019b; Leijten et al., 2013, 2016, 2018, 2020; Menting et al., 2013), Parent Child Interaction Therapy (Forster et al., 2012; Leijten et al., 2013), and Triple P (de Graaf et al., 2008; Forster et al., 2012; Leijten et al., 2013, ; Nogueira et al., 2022; Tully & Hunt, 2016). More broad-based psychoeducational or behavioral skills-based programs were also evaluated (Cai et al., 2022; Dretzke et al., 2005, 2009; Maughan et al., 2005). Most reviews on parenting treatment were of moderate to high quality, with the exception of one review rated low to moderate quality by Gardner et al. (2019a, 2019b). On average, small to moderate effect-sizes were reported (Cai et al., 2022; de Graaf et al., 2008; Dretzke et al., 2005, 2009; Forster et al., 2012; Furlong et al., 2012; Gardner et al., 2019a, 2019b; Leijten et al., 2013, 2016, 2018, 2020; Maughan et al., 2005; Menting et al., 2013; Mingebach et al., 2018), which were maintained at follow-up (Cai et al., 2022; de Graaf et al., 2008; Leijten et al., 2018). Notably, one meta-analysis, which focused solely on evaluating Parent Child Interaction Therapy, reported a large effect size in improving child behavior (Ward et al., 2016). Another meta-analysis, which evaluated a range of parenting interventions, also reported large effect sizes for Parent Child Interaction Therapy and reported small to moderate effect sizes for both Incredible Years and Triple P (Leijten et al., 2016). Furthermore, the effectiveness of behavioral-based parenting interventions extended to foster families, as demonstrated in two meta-analyses, in which effect sizes were found to be small to moderate when interventions were delivered to foster carers (Solomon et al., 2017; Uretsky & Hoffman, 2017).

Some moderators of efficacy were identified across these meta-analyses. Two reviews found that children with greater symptom severity showed greater improvement following intervention (de Graaf et al., 2008; Leijten et al., 2020) and two found stronger effect sizes in treatment, rather than prevention, trials (Gardner et al., 2019a, 2019b; Leijten et al., 2018; Menting et al., 2013). One review reported stronger effect sizes the greater the number of therapy sessions attended (Menting et al., 2013); another found that male children did better at follow-up (de Graaf et al., 2008), while another found that disadvantaged families showed less benefit by one-year follow-up (Leijten et al., 2013). However, age, delivery format (individual vs. group) and provider were not found to moderate efficacy of the programs delivered on child outcomes (Cai et al., 2022; de Graaf et al., 2008; Gardner et al., 2019a, 2019b).

The findings of the systematic reviews generally supported those of the meta-analyses. One systematic review focused on evaluating brief (< 8 sessions), behavioral parent training programs (such as Triple P and Parent Management Training Oregon) (Tully & Hunt, 2016). The researchers noted that all eight studies reported significant improvements in parent ratings for externalizing symptoms in children, with small to large effect sizes found for these programs. Another systematic review evaluated predictors of efficacy of behavioral-based parenting programs (including Incredible Years, Parent Child Interaction Therapy and Triple P) and reported some evidence for better outcomes in families with more positive child-parent relations (Dedousis-Wallace et al., 2021).

In addition to face-to-face behavioral parent training, three meta-analyses and one systematic review demonstrated that digitally assisted parent training (including self-directed parent training) (Tarver et al., 2014) was effective. Effect sizes ranged from small to moderate (Baumel et al., 2016 and 2017; Florean et al., 2020; Thongseiratch et al., 2020; Tarver et al., 2014) and gains were maintained at follow-up (Baumel et al., 2016). Stronger effect sizes were observed with a greater number of sessions (Florean et al., 2020), children’s difficulties being in the clinical range at baseline (compared to non-clinical children in middle school; Baumel et al., 2016), the inclusion of interactive elements in the digital treatment (compared to non-interactive digital treatment; Baumel et al., 2016), and sending reminders to parents/carers (Thongseiratch et al., 2020).

##### Behavioral, Cognitive, and Cognitive Behavioral Interventions for Externalizing Symptoms in Children

One moderate to high quality meta-analysis evaluated the efficacy of CBT, behavioral therapy and/or cognitive therapy interventions in managing externalizing symptoms and found a large effect size which was retained at follow-up (Riise et al., 2021). The interventions examined in this meta-analysis included behavioral-based parenting programs such as Incredible Years and PCIT, in addition to other forms of behavioral, cognitive, and/or cognitive behavioral intervention delivered directly with the child. The effect size did not differ as a function of therapy format (individual vs. group) or degree of parent, teacher and/or professional involvement. However, younger children (mean age = 8.2 years) and those with greater baseline symptoms showed greater improvement.

Behavior-based interventions were also found to be effective in reducing externalizing symptoms when delivered in a school setting in two meta-analyses and one systematic review (Nye et al., 2019; Smith et al., 2021; Veenman et al., 2018). These three reviews were of moderate to high quality. Two meta-analyses reported small to moderate effect sizes for behavioral-based classroom programs (Smith et al., 2021; Veenman et al., 2018), whilst a systematic review of the Incredible Year Teacher Classroom Management intervention indicated a moderate effect size (Nye et al., 2019). Length of treatment was related to outcome, such that briefer classroom interventions were found to be more effective (Veenman et al., 2018). Mixed results were found on the moderating effect of gender, including having no significant impact (Veenman et al., 2018), or behavioral-based programs being more effective in girls than boys (Smith et al., 2021). Conversely, age and severity of problems were not related to outcome (Veenman et al., 2018).

##### Child-Centered Play Therapy for Externalizing Symptoms in Children

One moderate quality meta-analysis (Parker et al., 2021a, b) found that child-centered play therapy led to reductions in externalizing and overall problem behaviors with medium effects. There were also reductions in aggressive behaviors, with small effects.

##### Child Social Skills Training for Externalizing Symptoms in Children

One high quality meta-analysis (Lösel & Beelmann, 2003) revealed that social skills interventions (predominately, but not exclusively, based on behavioral and/or cognitive model of social learning), yielded small to moderate effect sizes on antisocial behavior, with small effects maintained at follow-up. Social skills programs targeting at-risk children were found to be more effective than universal interventions.

##### Music Interventions for Externalizing Symptoms in Children

One moderate quality meta-analysis evaluated group-based music intervention and reported a large effect-size in reducing aggressive behaviors and a moderate effect-size in increasing self-control (Ye et al., 2021). However, children less than 10 years benefited less than older children, while more than one music session per week resulting in greater benefit than less frequent sessions.

#### Interventions for Children with Anxiety and Related Disorders

There were 18 reviews reporting on interventions targeting anxiety and/or related disorders/symptoms. These are evaluated below.

##### Mixed Psychosocial Interventions for Children with Anxiety and Related Disorders

Five meta-analyses and one systematic review reported on a wide range of psychosocial interventions for anxiety symptoms in children (Caldwell et al., 2019; Comer et al., 2019; Grist et al., 2019; Reynolds et al., 2012; Werner-Seidler et al., 2017, 2021). Four of the five meta-analyses were considered high quality, and one meta-analysis (Caldwell et al., 2019) was of moderate quality. Four out of five meta-analyses reported small to moderate effect sizes, demonstrating a positive impact of psychosocial interventions for children. Smaller effects were observed when interventions were compared to active control conditions and at follow-up. One meta-analysis did not find psychosocial interventions had any significant effect on anxiety following universal or targeted interventions delivered in primary schools (Caldwell et al., 2019). This review did report some, albeit weak, evidence in support of the efficacy of universal CBT interventions for reducing student anxiety. In further support of this effect, two additional reviews reported that CBT delivered stronger effects (moderate effect sizes) compared to non-CBT interventions (Grist et al., 2019; Reynolds et al., 2012). Results from one systematic review similarly concluded that CBT treatments were the only interventions that were probably efficacious to well-established (Comer et al., 2019). Individual interventions (vs group) and greater treatment length were both associated with stronger effects (Reynolds et al., 2012). Therapist assisted (vs self-help) and parental involvement increased effects of interventions (Comer et al., 2019; Grist et al., 2019).

##### Behavioral-Based Parenting Interventions for Children with Anxiety

One low quality meta-analysis investigated Parent–Child Interaction Therapy (PCIT) on youth anxiety and found that PCIT was effective at reducing anxious symptoms, with large effect sizes (Phillips & Mychailyszyn, 2021). PCIT was effective regardless of single diagnosis or comorbid diagnoses, and regardless of clinical status. The inclusion of family is also shown to be effective for cases of selective mutism, with one meta-analysis showing that combined behavioral and family systems approaches have the most supporting evidence for selective mutism (Steains et al., 2021).

##### Behavioral, Cognitive, and Cognitive Behavioral Interventions for Children with Anxiety and Related Disorders

Seven meta-analyses of moderate to high quality evaluated CBT-based interventions. One review solely focused on evaluating age effects and no significant differences emerged, concluding that CBT was effective in reducing anxiety symptoms across development (Bennet et al., 2013). The other six meta-analyses found that CBT significantly reduced anxiety symptoms in children (Ale et al., 2015; Fisak et al., 2011; Howes Vallis et al., 2020; McGuire et al., 2015; Viswanathan et al., 2022; Yin et al., 2021). Only one meta-analysis reported small effect-sizes (Fisak et al., 2011), while the other five reported moderate to large effect sizes on average. Beyond these meta-analyses, one low quality systematic review demonstrated that these findings provisionally extend to children with selective mutism, concluding that CBT is ‘promising’ in reducing anxiety symptoms in these children (Østergaard, 2018).

Regarding moderators of efficacy, two meta-analyses showed no difference in effects based on parental attendance at sessions (Ale et al., 2015; Howes Vallis et al., 2020); two showed no effect of intervention duration (Ale et al., 2015; Fisak et al., 2011); two showed no difference between individual and group formats (Ale et al., 2015; Howes Vallis et al., 2020); and, one showed no difference between universal compared to targeted CBT interventions (Fisak et al., 2011). Two moderators were identified: (i) CBT interventions administered by professionally qualified mental health providers had significantly better effects relative to minimal effects for interventions administered by laypersons (Fisak et al., 2011), and, (ii) in-person CBT interventions had significantly stronger effects than internet-based CBT interventions in young children (mean age = 5.45 years; Howes Vallis et al., 2020).

In terms of CBT components/types, Ale et al. (2015) found that CBT interventions that explicitly included exposure and response prevention for OCD had significantly stronger effects relative to other types of CBT interventions for other types of anxiety disorders (Ale et al., 2015). Exposure-based interventions also exhibited larger effects compared to cognitive therapies for OCD, although this effect was not statistically significant (McGuire et al., 2015). Finally, there was some conflicting evidence for the efficacy of cognitive bias modification interventions in reducing anxiety in children. One review reported a significant, yet small effect-size for reducing anxiety symptoms (Krebs et al., 2018), while another reported a minimal, non-significant effect-size for a very small number of trials (Grist et al., 2019).

##### Mindfulness Interventions for Children with Anxiety and Related Disorders

One moderate quality meta-analysis investigated the efficacy of mindfulness-based interventions on anxiety for children (Odgers et al., 2020). A small effect-size was reported overall, with the meta-analysis pooling the results from a small number of studies conducted in Iran that produced a significantly larger effect-size relative to studies conducted in Western countries, where the effects were found to be non-significant. This review does not support the use of mindfulness interventions for the reduction of anxiety in children.

#### Interventions for Children with Depressive Symptoms

Seven reviews reported on a range of interventions targeting depressive symptoms in children. These are evaluated below.

##### Mixed Psychosocial Interventions for Children with Depressive Symptoms

Five meta-analyses of moderate to high quality reviewed a range of psychosocial interventions for depression: two reported small but significant effect sizes (Werner-Seidler et al., 2017, 2021) and one failed to find any effect at post treatment for school-based interventions (Caldwell et al., 2019). However, Caldwell and colleagues (2019) did find that between 13 and 24 months follow-up, CBT-based targeted programs led to significant reductions in depressive symptoms, with a moderate effect-size. One meta-analysis examined response rates instead of effect sizes and Cuijpers et al. (2023) found that 39% of youth responded to treatment compared to 24% response rates in controls. However, of those that did respond, effects of response retained at 6–12 months. In further support of the medium-term effects, Werner-Seidler et al., (2017, 2021) also found that effects of school-based programs, predominantly comprising CBT components, were also evident at 12 months follow-up, although the effect-size was smaller.

##### Behavioral, Cognitive, and Cognitive Behavioral Interventions for Children with Depressive Symptoms

One low quality meta-analysis comprised a range of CBT programs, including computerized interventions, self-control therapy, and CBT combined with pharmacotherapy (Forti-Buratti et al., 2016). The authors reported non-statistically significant effects when CBT programs were compared to waitlist/no treatment conditions, showing a lack of evidence for CBT in successfully treating depression in children younger than 13 years of age. Conversely, another high quality review evaluated the efficacy of a range of psychotherapy programs, predominantly CBT, in managing depression in children and reported a moderate to large effect-size, which was retained at follow-up (Michael & Crowley, 2002). Larger effects were observed for adolescents older than 12 years of age compared to younger children. A low quality systematic review of psychotherapy programs for children with dysregulated mood showed some, albeit limited and preliminary, evidence of symptom improvement following psychological intervention (Benarous et al., 2017).

#### Interventions for children exposed to trauma

Eight reviews investigated the efficacy of interventions for children exposed to trauma. These are evaluated below.

##### Psychosocial Interventions for Children Exposed to Trauma

Three meta-analyses investigated the efficacy of psychosocial interventions on a range of trauma symptoms. Collectively, two high quality meta-analyses showed efficacy of interventions (including trauma-focused CBT and eye movement desensitization and processing) relative to control on symptoms of post-traumatic stress disorder (PTSD) (Bastien et al., 2020; Purgato et al., 2018), and one moderate quality meta-analysis demonstrated efficacy relative to control for bereavement and total mental health (Rosner et al., 2010) and in low-resource humanitarian settings (Purgato et al., 2018). The meta-analysis by Purgato et al. (2018) further showed that these small to moderate effects were retained at follow up. However, while efficacious for PTSD symptoms, these interventions did not influence depression and anxiety symptoms relative to control groups (Purgato et al., 2018), with one study (the only one eligible for inclusion) in the meta-analysis conducted by Bastien et al. (2020) showing that trauma-focused CBT was no more efficacious than waitlist control for children. Nevertheless, most studies indicated small to moderate pooled effect sizes (Bastien et al., 2020). Moreover, the effectiveness of psychosocial interventions for trauma symptoms, specifically children with a history of neglect (no matter the severity), provisionally extended to children in foster care, but requires more rigorous evaluation in community-based settings (Hambrick et al., 2016).

Regarding moderators of efficacy, Purgato et al. (2018) indicated that psychosocial interventions were more effective for non-displaced (versus displaced) children and those from smaller households (< six people versus > six people). However, there were mixed findings regarding the impact of age on outcome, with Hambrick et al. (2016) showing that younger children benefited more from the interventions, while Purgato et al. (2018) and Rosner et al. (2010) indicating stronger effects for children over the age of 12 years.

##### Behavioral-Based Parenting Interventions for Children Exposed to Trauma

One moderate quality meta-analysis investigated trauma-informed behavioral-based parenting interventions (Lindstrom Johnson et al., 2018). Such interventions had a moderate to large effect on child trauma symptoms, as well as on positive parenting practices, child Internalizing problems and child Externalizing problems. The type of trauma impacted efficacy, with greater effect sizes observed for child maltreatment-focused interventions compared to interventions that focused on intimate partner violence or family conflict. Moreover, longer interventions showed a stronger effect on Internalizing problems. Interestingly, efficacy did not differ as a function of child involvement.

##### Behavioral, Cognitive, and Cognitive Behavioral Interventions for Children Exposed to Trauma

One moderate quality systematic review examined trauma-focused cognitive-behavioral interventions (TF-CBT) for preschool children, aged 3- to 6-years (McGuire et al., 2021). The authors concluded that since few of the studies assessed efficacy in preschool children as well as the vast differences in treatment protocols for TF-CBT used with preschool aged children, TF-CBT is currently classified as “probably efficacious” intervention for preschool children. The authors also highlighted that when considering the use of TF-CBT for preschool-aged children with PTSD, clinicians must consider their cognitive abilities, family context and culture.

##### Child-Centered Play Therapy for Children Exposed to Trauma

Two systematic reviews focused on child-centered play therapy for children who have experienced trauma. One moderate quality systematic review found that child-centered play therapy was a promising intervention for children who experienced adverse childhood experiences, leading to reductions in externalizing and internalizing behavior and increases in parental empathy (Parker et al., 2021a). However, another low quality systematic review found that although some changes have been demonstrated pre-to post-intervention, this was not consistent across measurements and very few differences were demonstrated between treatment and control groups (Humble et al., 2019). Thus, the authors concluded that, presently, there is limited evidence to recommend child-centered play therapy for children who have experienced trauma.

#### Interventions for Children with Attention Deficit Hyperactivity Disorder (ADHD)

Overall, 35 reviews investigated the efficacy of interventions for ADHD symptoms. The bulk of the literature reviewed the efficacy of behavioral-based parenting interventions. The findings are summarized below.

##### Behavioral-Based Parenting Interventions for Children with ADHD

We identified six meta-analyses and seven systematic reviews that evaluated behavioral-based parenting interventions for children with ADHD. Most of these reviews evaluated a range of broad-based behavioral psychoeducational parent training interventions, and quality of reviews ranged from low to high. On average, the meta-analyses reported small to large effects in reducing ADHD symptoms, as well as comorbid externalizing and internalizing symptoms (Coates et al., 2015; Corcoran & Dattalo, 2006; Lee et al., 2012; Mulqueen et al., 2015; Rimestad et al., 2019; Zwi et al., 2011).

The findings of the systematic reviews were similar to the findings of the meta-analyses (Bjornstad & Montgomery, 2005; Ghuman et al., 2008; McGoey et al., 2002; Murray et al., 2018; Tan-MacNeill et al., 2021; Vacher et al., 2020; Vetter, 2018). Of note, systematic reviews were of low to moderate quality. Three systematic reviews evaluated specific interventions, namely Parent Child Interaction Therapy (PCIT) and the Incredible Years, showing they were efficacious for reducing parent and/or teacher reported ADHD symptoms (Ghuman et al., 2008; Murray et al., 2018; Vetter, 2018). One review evaluating various online parenting interventions also reported improvements in parent-rated ADHD symptoms overall (Tan-MacNeill et al., 2021). Two reviews also found beneficial effects on other child outcomes, such as social skills, emotion regulation and peer interaction (Murray et al., 2018; Vacher et al., 2020), and two reported improved parent–child interactions and parental confidence in managing child behavior (McGoey et al., 2002; Tan-MacNeill et al., 2021).

Notably however, one systematic review of low quality including two studies showed that a behavioral-based parenting intervention was not as effective as medication but did not differ from treatment as usual in the community (Bjornstad & Montgomery, 2005) and two indicated they were not efficacious when based on teacher reported ADHD symptoms (McGoey et al., 2002; Murray et al., 2018). Nevertheless, these authors concluded overall that behavioral-based parenting interventions had strong efficacy for some children and their families, and this depended on a number of moderating factors.

Some moderators of efficacy were considered across the meta-analyses and systematic reviews. Two meta-analyses showed no difference in efficacy depending on the delivery format of group vs individual (Lee et al., 2012; Rimestad et al., 2019), and two systematic reviews showed no differences depending on intervention duration and child involvement (Lee et al., 2012; Mulqueen et al., 2015). A systematic review focusing on the Incredible Years program showed there were no differences in effect sizes between studies that included the child-component and those that included the parent-component only (Murray et al., 2018). Notably however, one meta-analysis found that studies incorporating medication with behavioral based parenting interventions had significantly better effect sizes for ADHD symptoms than those without medication (Corcoran & Dattalo, 2006).

##### Behavioral, Cognitive, and Cognitive Behavioral Interventions for Children with ADHD

There were a total of 10 meta-analyses and one systematic review evaluating behavioral, cognitive and cognitive-behavioral interventions. Two recent high quality meta-analyses of RCTs investigated the efficacy of various behavioural treatments in ADHD (Groenman et al., 2022; Hornstra et al., 2023), demonstrating overall small to moderate improvements in ADHD symptoms, ODD and CD symptoms, and impairment (Groenman et al., 2022). Another moderate quality meta-analysis examined the efficacy of various behavioral interventions in managing ADHD that included parent, teacher and/or child sessions delivered across home, school and other contexts (Fabiano et al., 2009). A large effect size was reported. Behavior-based interventions were also found to be effective in reducing ADHD symptoms when delivered in a school setting in four very low to moderate quality studies (Gaastra et al., 2016; Harrison et al., 2019; Iznardo et al., 2020; Pyle & Fabiano, 2017). Two meta-analyses reported general improvements in outcomes for daily behavior report cards, with moderate to large effect sizes (Iznardo et al., 2020; Pyle & Fabiano, 2017). Two other meta-analyses showed behavioral interventions, instructional interventions and self-management interventions also had moderate efficacy. Mode of delivery was related to outcome, such that interventions implemented by a researcher were more effective than those implemented by a teacher (Harrison et al., 2019), and individual training led to larger effects than group training (Hornstra, 2023). Higher conduct or ADHD symptoms at baseline also led to greater intervention effects (Groenman, 2022). Notably, the addition of medication to behavioral-based interventions led to the largest effect sizes (Gaastra et al., 2016).

Other reviews have investigated specific behavioral interventions, including one moderate quality meta-analysis, which showed that behavior modification and neurofeedback interventions resulted in improvements in ADHD symptoms, such as hyperactivity, inattention, sociability and self-control (Hodgson et al., 2014). This effect was strongest for girls compared with boys, and for the combined subtype of ADHD compared with other subtypes. When examining the efficacy of cognitive behavioral interventions on ADHD and externalizing behaviors, Riise et al. (2021) found large but comparably smaller effects for ADHD than externalizing behaviors in a moderate to high quality study. Furthermore, Wilkes-Gillan et al. (2021) investigated video-modelling as an intervention technique for behavior change in ADHD. In this low/moderate quality review, Wilkes-Gillan et al. (2021) found overall improvements in social targets, such as social skills and friendship quality. Pauli-Pott et al. (2021) found in a high-quality meta-analysis that cognitive interventions targeting executive functioning led to positive outcomes on ADHD and ODD symptoms with small to moderate effect sizes in children with this dual diagnosis.

##### Mixed Psychosocial Interventions for Children with ADHD

One moderate quality meta-analysis, one high quality meta-meta-analysis, and one high quality systematic review evaluated the efficacy of psychosocial interventions on ADHD, but all three considered these in combination with medication (Arnold et al., 2015; Türk et al., 2023; Van der Oord et al., 2008). All three studies showed that psychosocial interventions were moderately efficacious in reducing ADHD symptoms, but when combined with medication larger effect sizes were achieved. Interestingly, two studies (Arnold et al., 2015; Van der Oord et al., 2008) showed that treatment duration did not influence the efficacy of combined psychosocial interventions with medication.

##### Child Social Skills Training for Children with ADHD

A meta-analysis conducted by Storebo et al. (2019) revealed that social skills interventions were associated with small to moderate effect sizes on teacher and parent reported ADHD symptoms, as well as on social skills, emotional competence, and general behavior. The meta-analysis by Storebo et al. (2019) was considered high quality. Two moderate quality systematic reviews similarly found child social skills training to be efficacious for ADHD (Fox et al., 2020; Willis et al., 2019). 

##### Self-Regulation Interventions for Children with ADHD

ADHD was the only condition for which self-regulation interventions were specifically reviewed. One meta-analysis by Reid et al. (2005) on self-regulation interventions found that these interventions are efficacious for elementary age children (under 12 years of age) with ADHD. These interventions were conducted across a range of settings, including school, community, and clinic, and demonstrated efficacy in improving ADHD behaviors, such as an increase in on-task behavior and a decrease in inappropriate or disruptive behaviors. Notably, the findings reported by Reid et al. (2005) suggest that the effects of medication combined with self-regulation interventions may be more efficacious than the intervention or medication alone. However, conclusions should also be taken with caution as the quality of the review was considered low.

##### Child-Centered Play Therapy for Children with ADHD

Two small systematic reviews examined play-based interventions conducted by occupational therapists in school-based settings (Brooks & Bannigan, 2021; Cornell et al., 2018). Although both reviews reported positive intervention outcomes, including improved social play skills, empathy, and occupational performance, these interventions still cannot be considered an evidence-based practice for ADHD at the present time given an insufficient amount of high-quality evidence (Brooks & Bannigan, 2021; Cornell et al., 2018). Indeed, these reviews were rated as low (Brooks & Bannigan, 2021) and moderate (Cornell et al., 2018) quality.

##### Meditation and Mindfulness Interventions for Children with ADHD

One small systematic review on meditation for ADHD in the classroom found that there is insufficient evidence to support its efficacy for ADHD due to the limited number of RCTs conducted on this intervention type for children with ADHD and all reported inconsistent results (Krisanaprakornkit et al., 2010). Conversely, one meta-analysis by Vekety et al. (2021) denoted that mindfulness-based interventions reduced teacher-rated inattentive and hyperactive-impulsive behaviors with small effect sizes. However, these two reviews were also considered low to moderate quality. Overall, further evidence is required to understand the efficacy of meditation and mindfulness-based interventions (Krisanaprakornkit et al., 2010; Vekety et al., 2021).

#### Interventions for Children with Autism Spectrum Disorders (ASD)

Seventeen reviews investigated the efficacy of interventions for a range of emotional, behavioral or social problems in children with a diagnosis of ASD. These are evaluated below.

##### Behavioral-Based Interventions for Children with ASD

Two meta-analyses and one systematic review examined behavior-based interventions for children with ASD (Camargo et al., 2016; Tarver et al., 2019; Vetter et al., 2018). One high quality meta-analysis showed moderate effect-sizes following behavioral parent interventions on child disruptive behavior and hyperactivity (Tarver et al., 2019). Another meta-analysis showed that a broad range of behavioral-based interventions (incorporating prompting, modelling, reinforcement and imitation skills) were efficacious for improving social interaction skills in children with ASD, with similar gains across different age groups (Camargo et al., 2016). However, Camargo et al. (2016) had a low-quality rating, so interpretations should be cautious. Another low quality systematic review described nine non-controlled studies investigating the efficacy of Parent Child Interaction Therapy that had been extensively adapted for use with children with ASD (Vetter, 2018). Positive outcomes were reported for child problem behavior, ADHD-related disruptive behaviors, parental stress, parent–child interactions, and socialization.

##### Social Skills Interventions for Children with ASD

Ten reviews investigated social skills interventions for children with ASD. Six meta-analyses reported that social skills interventions led to positive outcomes with small to large effect-sizes (Reichow et al., 2013; Wahman et al., 2022; Whalon et al., 2015; Wang et al., 2011, 2013; Wang & Spillane, 2009). The only high quality meta-analysis evaluating RCTs provided evidence that social-skills groups improve social competence and friendship quality in this population (Reichow et al., 2013). The other five meta-analyses were of very low or low quality. Despite that, Whalon et al. (2015) demonstrated that children with ASD can benefit from social skills interventions implemented with peers in school settings, as well as from adult-mediated, child-specific and multi-component interventions, with large effect-sizes reported. One meta-analysis evaluated peer-mediated and video-modelling interventions, and concluded from 14 single case-studies that these interventions improved the social performance of children with ASD (Wang et al., 2011). Four systematic reviews of very low or low quality reported similar results that social skills interventions improved communication skills and parent–child interactions, with peer-related interventions for pre-school aged children with ASD showing generalization and maintenance of outcomes (Camargo et al., 2014; Gunning et al., 2019; Tan-MacNeill et al., 2021; Wright et al., 2016). Imitation interventions had inconsistent or non-significant effects (Tan-MacNeill et al., 2021).

Regarding notable moderators, there were mixed conclusions regarding the effect of age on the efficacy of social skills interventions. One meta-analysis of children aged 4- to 15-years reported that interventions were more efficacious for younger than older children (Wang et al., 2011). Conversely, two meta-analyses showed that age did not moderate outcomes with similar gains seen across the different age groups following intervention (Wang et al., 2013; Whalon et al., 2015).

##### Social Stories for Children with ASD

The two meta-analyses examining social stories yielded conflicting findings. A meta-analysis of single cases-studies demonstrated that social stories had low to questionable overall efficacy (Kokina & Kern, 2010). There was some evidence that stories were more efficacious when addressing inappropriate behavior than when teaching social skills to children with ASD, and when delivered to primary school age children (6- to 11-years) rather than younger children. In contrast, a second meta-analysis of case series designs reported a moderate effect-size and concluded social stories were efficacious interventions for reducing inappropriate and increasing appropriate social behavior in children with ASD (Aldabas, 2019). However, social stories at this stage cannot be considered evidence-based interventions for ASD, as both meta-analyses were considered of very low quality.

##### Sensory-Based Interventions for Children with ASD

There was one moderate quality systematic review examining the efficacy of sensory-based interventions in children with ASD (Weitlauf et al., 2017). The authors reported modest short-term positive effects of these approaches on sensory and motor skills/challenges, ASD symptoms, receptive language, verbal and nonverbal communication, nonverbal cognitive skills, joint-attention and social engagement. However, these conclusions relied on small, short-term studies incorporating different protocols and addressing different outcomes. 

##### Interventions to Improve Anxiety in Children with an ASD Diagnosis

One low quality systematic review into effective treatments for anxiety in children with an ASD diagnosis indicated that CBT interventions were the most well-researched and had the most support. In contrast, the authors of this review stated that there was little evidence for social stories, sensory-integrative interventions, or standalone exposure as being effective in targeting anxiety in this population (Slaughter et al., 2020).

## Discussion

On the basis of synthesized review findings, there are several available interventions, mostly, behavioral- and/or CBT-based interventions, which have an overwhelmingly substantial body of evidence in support of their efficacy in supporting social, emotional, and behavioral needs, and can thus be recommended for wide-spread implementation for children ages 4- to 9-years-old. Currently, there is less evidence to recommend wide-scale implementation of non-behavioral or non-cognitive-behavioral interventions for programs targeting children ages 4- to 9-years, though there are various other interventions that seem promising for specific mental health difficulties. The discussion below will include a synthesis of the evidence base that primarily focuses on papers of moderate to high quality.

When targeting mental health difficulties broadly in children, papers of moderate to high quality suggested that behavioral based parenting interventions had the strongest evidence and were efficacious in reducing externalizing symptoms and disruptive behaviors, as well as improving social skills. Across these reviews, smaller, yet significant effect sizes were also found for the improvement of internalizing symptoms for behavioral-based parenting interventions. Furthermore, there was promising evidence across another eight reviews for the efficacy of socio-emotional interventions, in particular, for interventions focusing on emotion understanding, emotion socialization, or attachment. Regarding other interventions, there were a few reviews of art therapy, and positive psychology interventions for managing general distress in young children. These interventions showed improvements in target outcomes, however, conclusions are limited by the small number of studies on these interventions. Overall, this suggests that CBT-based parenting interventions have the strongest evidence base for child mental health difficulties broadly.

Substantial evidence (44 reviews) emerged regarding specific interventions for externalizing symptoms in children. For such children, 24 moderate to high quality reviews concluded that individual and group behavioral and CBT parent-training programs, as well as mixed psychosocial interventions were shown to be efficacious with, on average, small to moderate effect-sizes found at post-intervention and follow-up. Evidence was also found for the efficacy of behavioral and cognitive-behavioral-based interventions overall in four moderate to high quality reviews, with behavioral interventions shown to be effective also when delivered with the child or in a school setting. Beyond this, individual reviews of moderate to high quality demonstrated preliminary evidence that CBT-based social skills training programs, and music interventions reduced externalizing problems.

Regarding internalizing difficulties, one high quality meta-analysis found that CBT-based programs were efficacious in reducing internalizing symptoms in children with on average moderate to large effect-sizes. Despite a small number of studies investigating interventions for internalizing symptoms, of the internalizing disorders, there was substantial evidence (18 reviews) evaluating the effect of CBT programs on anxiety disorders. Across six of seven meta-analyses of moderate to high quality, CBT was also shown to lead to moderate to large effect sizes. One meta-analysis indicated small effect sizes, with the smaller effect size potentially related to methodological differences, such as the inclusion of unpublished papers and this study including only prevention programs. Furthermore, one moderate quality meta-analysis similarly found a parent-based behavioral intervention (PCIT) was efficacious on internalizing symptoms with large effects. However, other interventions that were not behavioral or cognitive-behavioral showed small to minimal effects.

There were a small number of reviews that investigated interventions for children experiencing depressive symptoms (7 reviews) or those exposed to trauma (8 reviews). There is provisional evidence from moderate to high quality papers that psychosocial interventions, notably CBT programs, contribute to a reduction in depressive symptoms in children. A stronger evidence base is required to determine which specific components of CBT are effective and which specific formats and duration of treatments are most beneficial for the reduction of depressive symptoms in children. There are also two high-quality papers suggesting that psychosocial interventions overall are efficacious for trauma symptoms, though more studies are needed to understand which interventions are best. Individual moderate quality reviews also showed preliminary evidence for the efficacy of behavioral-based parenting interventions, trauma-focused CBT, and child-centered play therapy for trauma symptoms.

Beyond that, there was substantial evidence (35 reviews) for interventions supporting children with ADHD symptoms. Across 12 reviews, behavioral parent-training interventions improved ADHD symptoms and comorbid externalizing and internalizing symptoms, with small to large effect-sizes at post-intervention and follow-up. Notably, the quality of reviews for ADHD interventions varied from low to high, though the higher quality reviews (of moderate to high quality) reported moderate to large effect sizes of behavioral parent-training. In addition, a smaller number of moderate quality reviews reported that interventions based on behavioral therapy and CBT more broadly also appeared efficacious in improving ADHD symptoms and one high quality meta-analysis indicated that social skills interventions were promising. Some studies also suggested that combined medication and psychosocial treatments may be superior to either behavioral parent-training or medication alone.

The only two high quality studies in ASD demonstrated the efficacy of behavior-based parent-training and social skills interventions in reducing mental health difficulties in children with ASD. However, the current evidence base is limited as it primarily relies on single case-study designs.

We utilized an exploratory and narrative synthesis of evidence regarding moderators of efficacy. There was heterogeneity on types of moderators examined as well as insufficient power across many reviews to conduct quantitative moderator analyses. However, multiple reviews suggested that children with greater baseline symptom severity tended to benefit more so from interventions, including externalizing (Baumel et al., 2016; de Graaf et al., 2008; Leijten et al., 2020; Riise et al., 2021) and anxiety symptoms (Grist et al., 2019; Howes Vallis et al., 2020). There was also consistent evidence that treatment and selective or indicated prevention interventions yield greater efficacy, compared to universal interventions (2019b; Gardner et al., 2019a; Lösel & Beelmann, 2003; Sanchez et al., 2018; Yap et al., 2016). However, there was mixed evidence for other moderators. For example, there was some evidence to suggest that including both child and parental sessions may be more beneficial in managing externalizing symptoms than parents only interventions (Battagliese et al., 2015) and also more beneficial for anxiety in children than child only interventions (Comer et al., 2019; Grist et al., 2019). Conversely, no clear evidence emerged that including children in interventions for ADHD symptoms increased intervention efficacy over including parents alone (Lee et al., 2012; Mulqueen et al., 2015).

There was also inconsistency in the findings from reviews regarding the impact of treatment length on intervention efficacy. For broad mental health interventions, one review found longer treatments more effective (Carr et al., 2017), whereas another found that number of session hours did not moderate outcome (Buchanan-Pascall et al., 2018). Similarly, for interventions for externalizing symptoms, one review found that treatment length did not moderate response (Comer et al., 2013), another found that brief parenting interventions were effective in reducing child externalizing behaviors (Tully & Hunt, 2016), while others still found that number or intensity of intervention sessions positively predicted intervention effects (Carr et al., 2017; Dretzke et al., 2005; Florean et al., 2020; Menting et al., 2013). Findings for anxiety interventions were similarly mixed, with some papers showing that greater treatment length predicted stronger effects (McGuire et al., 2015; Reynolds et al., 2012), while others showed no effect of treatment length (Ale et al., 2015; Fisak et al., 2011; Krebs et al., 2018). Conversely, for interventions for ADHD symptoms, duration of intervention was not found to influence efficacy (Arnold et al., 2015; Hodgson et al., 2014; Mulqueen et al., 2015; Van der Oord et al., 2008). Thus, the current research base does not at present provide a ‘gold standard’ for treatment length in terms of managing childhood emotional, behavioral, and social problems.

Mixed findings also emerged regarding intervention format. For example, interventions for externalizing symptoms appeared to be efficacious regardless of format of therapy (Comer et al., 2013; de Graaf et al., 2008; Riise et al., 2021); however, one meta-analysis favored individual formats (Fossum et al., 2016). Similarly, for anxiety interventions, some reviews showed no difference between individual and group delivery (Ale et al., 2015; Howes Vallis et al., 2020), while another showed that individual interventions delivered stronger effects on child-reported symptoms (Reynolds et al., 2012). For ADHD interventions, two reviews showed no difference between individual and group delivery of behavioral parent-training interventions (Lee et al., 2012; Rimestad et al., 2019), whereas one recent high quality study found individual behavioral interventions led to larger effects than group delivery (Hornstra, 2023). Collectively, these findings indicate that individual and group-based programs may both have benefits for reducing emotional, behavioral, and social problems in children.

The limitations of the current review must be acknowledged. We limited our search to published meta-analyses and systematic reviews. The emerging literature regarding new approaches would not have been detected by our review if the new approach had not yet accumulated sufficient original research papers to warrant a review paper. We also did not include grey literature in our review. We did not distinguish between symptom reporter in our summary of findings, meaning that we cannot say with confidence whether the current results will hold across child, parent, or observer/clinician reports. Furthermore, we did not consider cost-effectiveness within this review, however would encourage future reviews to do so, given its importance for implementation and policy makers. Lastly, given the heterogeneity of interventions and outcomes within early childhood interventions, we could not conduct any quantitative syntheses of results across studies. Meta-analytic methods are required to make firmer conclusions about the efficacy of various interventions.

## Conclusion

Mental disorders are prevalent in children, cause significant distress and lead to significant lifetime burden. Children who experience clinically significant mental health problems do not receive adequate treatment compared to older individuals. An overwhelmingly substantial body of quality evidence was collected as part of this review showing convincingly that we can alter this trend immediately through widespread implementation of targeted intervention programs in the early schooling years. The data showed that targeted interventions lead to better outcomes than universal intervention. Thus, targeted intervention programs should be made available to young children and their families. Parent-based behavioral and cognitive-behavioral interventions had the strongest evidence base, with many moderate to high quality papers supporting its efficacy, for broad mental health difficulties, externalizing issues, and ADHD. There was also substantial support for CBT-based programs for internalizing difficulties, especially in anxiety disorders.

The bulk of the evidence so far has not led to the identification of robust moderators that would allow us to conclude that interventions should definitely be modified for different children. The evidence suggests that children with greater symptom severity benefit more than children with less severity. Behavioral and cognitive behavioral interventions can be delivered in either in group or individual format, with the exception of ADHD, where individual treatment may lead to stronger outcomes. This does not suggest that group treatment is not effective for ADHD, just that individual treatment leads to stronger effects. If resources are not limited in a particular setting offering ADHD treatment, then individual treatment is recommended but if resources are limited, group treatments should still be offered. There is no conclusive evidence regarding whether these treatments should be delivered to the child, parent, or to both the parent and child; with the exception of programs specifically targeting externalizing symptoms or specifically targeting anxiety symptoms. For these problem types, there is evidence that including both parents and children delivers better outcomes than parent or child alone. Taken together, when resources are not limited, parents and children should be included when targeting externalizing and anxiety symptoms. Finally, at present, there is no indication to consistently determine an ideal treatment length, with brief treatments and longer treatments producing similar effects.

We identified a number of gaps for future research. There is less literature on internalizing disorders compared to externalizing disorders in children, and interventions that focus on broad mental health concerns led to smaller effects specifically for internalizing symptoms. This is due to the greater historical focus on externalizing disorders, potentially representing scope for further research to improve efficacy in interventions that target broad mental health symptoms. Of note, reviewing both broad and disorder-specific interventions allowed for a better understanding of how broader interventions can be improved to target specific subgroups. For example, one core component of anxiety interventions is exposure, yet this tends not to be included in broad-based mental health interventions. This may partially contribute to the smaller effect sizes of broad mental health interventions in internalizing difficulties. Lastly, although some interventions seemed promising, there is still an insufficient number of high-quality studies to make strong conclusions regarding recommended interventions for depression, trauma, and ASD in children from 4 to 9 years old.

The current review provides a valuable contribution to the mental health intervention field, by reviewing interventions for not just one, but a constellation of mental health problems in children. We anticipate this review will be useful for those delivering interventions for children (and parents/carers) struggling with their mental health in the initial years of primary school. For governments, schools and practitioners, there is a substantial body of evidence supporting the efficacy of behavioral- and/or CBT-based interventions, for childhood emotional, behavioral, and social problems, which can and should be, as a matter of urgency, implemented with 4–9-year-old children. When selecting broad-based interventions that target mental health, that is, interventions designed to reduce both internalizing and externalizing symptoms, we propose that these interventions should also include specific strategies that target internalizing symptoms such as anxiety. Future research endeavors should focus on increasing implementation and access for young children struggling with their mental health as well as building the evidence base for depression, trauma, and ASD.

## Supplementary Information

Below is the link to the electronic supplementary material.

Supplementary file1 (DOCX 50 KB)

## Footnotes

1 Although this range appears contrary to the defined developmental focus of this paper, studies outside the early schooling years were only included if mean age of the study was within the 4–9 age range and/or specific synthesis occurred for this target age group.
