# Supplementary material for: Interventions for Young Children’s Mental Health: A Review of Reviews
Source: Clin Child Fam Psychol Rev. 2023 Jul 24;26(3):593–641. doi: 10.1007/s10567-023-00443-6 (PMC10465658; doi:10.1007/s10567-023-00443-6)
Supplement: Supplementary file 1 — Supplementary file1 (DOCX 50 KB) [file 10567_2023_443_MOESM1_ESM.docx]

Supplementary Material 1: Search Strategy

Search Terms

*Broad Mental Health Review Search Terms*

1. “family-focused intervent*” OR “early intervent*” OR prevent* OR transdiagnostic OR “targeted group” OR “group therap*” OR “peer group” OR “peer support” OR “targeted therap*” OR “targeted intervent*” OR “indicated intervent*” OR intervent* OR treat* OR “child intervent*” OR “school-based intervent*” OR “parent-child intervent*” OR “teacher-led program*” OR “teacher-led intervent*” OR “parent-led program*” OR “parent-led intervent*” OR “clinician-led program*” OR “clinician-led intervent*”
2. “mental health” OR “behavioural problem*” OR “behavioral problem*” OR “emotional problem*” OR “social problem*”
3. #1 AND #2

*Anxiety Review Search Terms:*

1. “family-focused intervent*” OR “early intervent*” OR prevent* OR transdiagnostic OR “targeted group” OR “group therap*” OR “peer group” OR “peer support” OR “targeted therap*” OR “targeted intervent*” OR “indicated intervent*” OR intervent* OR treat* OR “child intervent*” OR “school-based intervent*” OR “parent-child intervent*” OR “teacher-led program*” OR “teacher-led intervent*” OR “parent-led program*” OR “parent-led intervent*” OR “clinician-led program*” OR “clinician-led intervent*”
2. Anxi* OR internalizing OR internalizing OR worr*
3. #1 AND #2

*Depression Review Search Terms:*

1. “family-focused intervent*” OR “early intervent*” OR prevent* OR transdiagnostic OR “targeted group” OR “group therap*” OR “peer group” OR “peer support” OR “targeted therap*” OR “targeted intervent*” OR “indicated intervent*” OR intervent* OR treat* OR “child intervent*” OR “school-based intervent*” OR “parent-child intervent*” OR “teacher-led program*” OR “teacher-led intervent*” OR “parent-led program*” OR “parent-led intervent*” OR “clinician-led program*” OR “clinician-led intervent*”
2. Depress* OR mood OR internalizing OR internalizing OR sad* OR low OR unhapp*
3. #1 AND #2

*Conduct Problems Review Search Terms:*

1. “family-focused intervent*” OR “early intervent*” OR prevent* OR transdiagnostic OR “targeted group” OR “group therap*” OR “peer group” OR “peer support” OR “targeted therap*” OR “targeted intervent*” OR “indicated intervent*” OR intervent* OR treat* OR “child intervent*” OR “school-based intervent*” OR “parent-child intervent*” OR “teacher-led program*” OR “teacher-led intervent*” OR “parent-led program*” OR “parent-led intervent*” OR “clinician-led program*” OR “clinician-led intervent*”
2. Conduct OR CD OR aggress* OR “oppositional defiant disorder*” OR “oppositional defiant” OR oppositional OR ODD OR externalizing OR externalizing OR misbehaviour OR misbehavior OR fight* OR cheating OR violen* OR defian* OR ang* OR argu*
3. #1 AND #2

*ADHD Review Search Terms:*

1. “family-focused intervent*” OR “early intervent*” OR prevent* OR transdiagnostic OR “targeted group” OR “group therap*” OR “peer group” OR “peer support” OR “targeted therap*” OR “targeted intervent*” OR “indicated intervent*” OR intervent* OR treat* OR “child intervent*” OR “school-based intervent*” OR “parent-child intervent*” OR “teacher-led program*” OR “teacher-led intervent*” OR “parent-led program*” OR “parent-led intervent*” OR “clinician-led program*” OR “clinician-led intervent*”
2. ADHD OR attention OR “attention deficit” OR hyper* OR concentrat*
3. #1 AND #2

*Emotion Dysregulation Review Search Terms:*

1. “family-focused intervent*” OR “early intervent*” OR prevent* OR transdiagnostic OR “targeted group” OR “group therap*” OR “peer group” OR “peer support” OR “targeted therap*” OR “targeted intervent*” OR “indicated intervent*” OR intervent* OR treat* OR “child intervent*” OR “school-based intervent*” OR “parent-child intervent*” OR “teacher-led program*” OR “teacher-led intervent*” OR “parent-led program*” OR “parent-led intervent*” OR “clinician-led program*” OR “clinician-led intervent*”
2. “emotion dysregulation” OR “emotion regulation” OR “emotional regulation” OR “affect regulation” OR “self regulation” OR tantrum* OR outburst* OR meltdown OR “emotion coaching”
3. #1 AND #2

*Social Skills Review Search Terms:*

1. “family-focused intervent*” OR “early intervent*” OR prevent* OR transdiagnostic OR “targeted group” OR “group therap*” OR “peer group” OR “peer support” OR “targeted therap*” OR “targeted intervent*” OR “indicated intervent*” OR intervent* OR treat* OR “child intervent*” OR “school-based intervent*” OR “parent-child intervent*” OR “teacher-led program*” OR “teacher-led intervent*” OR “parent-led program*” OR “parent-led intervent*” OR “clinician-led program*” OR “clinician-led intervent*”
2. “social skill*” OR “friendship problem*” OR bully* OR bulli* OR “interpersonal problem*” OR “social problem*” OR “peer problem*” OR “peer relation*” OR “social relation*” OR connection OR “peer skills” OR “prosocial”
3. #1 AND #2

*Attachment Review Search Terms:*

1. “family-focused intervent*” OR “early intervent*” OR prevent* OR transdiagnostic OR “targeted group” OR “group therap*” OR “peer group” OR “peer support” OR “targeted therap*” OR “targeted intervent*” OR “indicated intervent*” OR intervent* OR treat* OR “child intervent*” OR “school-based intervent*” OR “parent-child intervent*” OR “teacher-led program*” OR “teacher-led intervent*” OR “parent-led program*” OR “parent-led intervent*” OR “clinician-led program*” OR “clinician-led intervent*”
2. Attachment OR “parent-child relationship*” OR “parent-child connection*” OR “attachment style*” OR “reactive attachment” OR “secure attachment” OR “insecure attachment” OR “avoidant attachment” OR “attachment rupture” OR “dismissive attachment” OR “disorganised attachment” OR “disorganized attachment”
3. #1 AND #2

*Sensory Regulation Search Terms:*

1. “family-focused intervent*” OR “early intervent*” OR prevent* OR transdiagnostic OR “targeted group” OR “group therap*” OR “peer group” OR “peer support” OR “targeted therap*” OR “targeted intervent*” OR “indicated intervent*” OR intervent* OR treat* OR “child intervent*” OR “school-based intervent*” OR “parent-child intervent*” OR “teacher-led program*” OR “teacher-led intervent*” OR “parent-led program*” OR “parent-led intervent*” OR “clinician-led program*” OR “clinician-led intervent*”
2. “sensory-regulation” OR “sensory processing difficult*” OR “sensory processing disorder*”
3. #1 AND #2

*Physical Wellbeing Search Terms:*

1. “family-focused intervent*” OR “early intervent*” OR prevent* OR transdiagnostic OR “targeted group” OR “group therap*” OR “peer group” OR “peer support” OR “targeted therap*” OR “targeted intervent*” OR “indicated intervent*” OR intervent* OR treat* OR “child intervent*” OR “school-based intervent*” OR “parent-child intervent*” OR “teacher-led program*” OR “teacher-led intervent*” OR “parent-led program*” OR “parent-led intervent*” OR “clinician-led program*” OR “clinician-led intervent*”
2. “physical wellbeing” OR “physical well being”
3. #1 AND #2

*Trauma (at-risk children) Population Search Terms:*

1. “family-focused intervent*” OR “early intervent*” OR prevent* OR transdiagnostic OR “targeted group” OR “group therap*” OR “peer group” OR “peer support” OR “targeted therap*” OR “targeted intervent*” OR “indicated intervent*” OR intervent* OR treat* OR “child intervent*” OR “school-based intervent*” OR “parent-child intervent*” OR “teacher-led program*” OR “teacher-led intervent*” OR “parent-led program*” OR “parent-led intervent*” OR “clinician-led program*” OR “clinician-led intervent*”
2. Trauma OR PTSD OR “post-traumatic stress disorder” OR “posttraumatic stress disorder” OR “trauma-informed” OR trauma OR stress OR “adverse child experience*” OR divorce OR war OR assault* OR abuse* OR neglect OR “household dysfunction” OR “physical abuse” OR “emotional abuse” OR “sexual abuse” OR “physical neglect” OR “emotional neglect” OR “mental illness” OR “incarcerated relative” OR “mother treated violently” OR “domestic violence” OR “substance abuse”
3. #1 AND #2

*Indigenous Australian Population Search Terms:*

1. “family-focused intervent*” OR “early intervent*” OR prevent* OR transdiagnostic OR “targeted group” OR “group therap*” OR “peer group” OR “peer support” OR “targeted therap*” OR “targeted intervent*” OR “indicated intervent*” OR intervent* OR treat* OR “child intervent*” OR “school-based intervent*” OR “parent-child intervent*” OR “teacher-led program*” OR “teacher-led intervent*” OR “parent-led program*” OR “parent-led intervent*” OR “clinician-led program*” OR “clinician-led intervent*”
2. Indigenous OR aboriginal OR “Torres Strait Islander”
3. #1 AND #2

Search History

| **PSYCINFO** | | | | |
| --- | --- | --- | --- | --- |
| **Search #** | **Key words** | **Restrictions** | **Search Field** | **# of Results** |
| **1** | “family-focused intervent*” OR “early intervent*” OR prevent* OR transdiagnostic OR “targeted group” OR “group therap*” OR “peer group” OR “peer support” OR “targeted therap*” OR “targeted intervent*” OR “indicated intervent*” OR intervent* OR treat* OR “child intervent*” OR “school-based intervent*” OR “parent-child intervent*” OR “teacher-led program*” OR “teacher-led intervent*” OR “parent-led program*” OR “parent-led intervent*” OR “clinician-led program*” OR “clinician-led intervent*” | Methodology: Meta-analysis, systematic review;  Language: English;  Date: Since 2000;  Age group: Preschool Age (2-5 Yrs), School Age (6-12 Yrs) | Full text |  |
| **2** | ti,ab(“family-focused intervent*” OR “early intervent*” OR prevent* OR transdiagnostic OR “targeted group” OR “group therap*” OR “peer group” OR “peer support” OR “targeted therap*” OR “targeted intervent*” OR “indicated intervent*” OR intervent* OR treat* OR “child intervent*” OR “school-based intervent*” OR “parent-child intervent*” OR “teacher-led program*” OR “teacher-led intervent*” OR “parent-led program*” OR “parent-led intervent*” OR “clinician-led program*” OR “clinician-led intervent*”) | Methodology: Meta-analysis, systematic review;  Language: English;  Date: Since 2000;  Age group: Preschool Age (2-5 Yrs), School Age (6-12 Yrs) | Title, Abstract | 785 |
| **3** | “mental health” OR “behavioural problem*” OR “behavioral problem*” OR “emotional problem*” OR “social problem*” | Methodology: Meta-analysis, systematic review;  Language: English;  Date: Since 2000;  Age group: Preschool Age (2-5 Yrs), School Age (6-12 Yrs) | Full text | 265 |
| **4** | ti,ab(“mental health” OR “behavioural problem*” OR “behavioral problem*” OR “emotional problem*” OR “social problem*”) | Methodology: Meta-analysis, systematic review;  Language: English;  Date: Since 2000;  Age group: Preschool Age (2-5 Yrs), School Age (6-12 Yrs) | Title, Abstract | **131** |
| **5** | depress* OR mood OR internalizing OR internalizing OR sad* OR low OR unhapp* | Methodology: Meta-analysis, systematic review;  Language: English;  Date: Since 2000;  Age group: Preschool Age (2-5 Yrs), School Age (6-12 Yrs) | Full text | **380** |
| **6** | ti,ab(depress* OR mood OR internalizing OR internalizing OR sad* OR low OR unhapp*) | Methodology: Meta-analysis, systematic review;  Language: English;  Date: Since 2000;  Age group: Preschool Age (2-5 Yrs), School Age (6-12 Yrs) | Title, Abstract | **353** |
| **7** | Anxi* OR internalizing OR internalizing OR worr* | Methodology: Meta-analysis, systematic review;  Language: English;  Date: Since 2000;  Age group: Preschool Age (2-5 Yrs), School Age (6-12 Yrs) | Full text | **164** |
| **8** | ti,ab(Anxi* OR internalizing OR internalizing OR worr*) | Methodology: Meta-analysis, systematic review;  Language: English;  Date: Since 2000;  Age group: Preschool Age (2-5 Yrs), School Age (6-12 Yrs) | Title, Abstract | **141** |
| **9** | Conduct OR CD OR aggress* OR “oppositional defiant disorder*” OR “oppositional defiant” OR oppositional OR ODD OR externalizing OR externalizing OR misbehaviour OR misbehavior OR fight* OR cheating OR violen* OR defian* OR ang* OR argu* | Methodology: Meta-analysis, systematic review;  Language: English;  Date: Since 2000;  Age group: Preschool Age (2-5 Yrs), School Age (6-12 Yrs) | Full text | **244** |
| **10** | ti,ab(Conduct OR CD OR aggress* OR “oppositional defiant disorder*” OR “oppositional defiant” OR oppositional OR ODD OR externalizing OR externalizing OR misbehaviour OR misbehavior OR fight* OR cheating OR violen* OR defian* OR ang* OR argu*) | Methodology: Meta-analysis, systematic review;  Language: English;  Date: Since 2000;  Age group: Preschool Age (2-5 Yrs), School Age (6-12 Yrs) | Title, Abstract | **203** |
| **11** | ADHD OR attention OR “attention deficit” OR hyper* OR concentrat* | Methodology: Meta-analysis, systematic review;  Language: English;  Date: Since 2000;  Age group: Preschool Age (2-5 Yrs), School Age (6-12 Yrs) | Full text | **162** |
| **12** | ti,ab(ADHD OR attention OR “attention deficit” OR hyper* OR concentrat*) | Methodology: Meta-analysis, systematic review;  Language: English;  Date: Since 2000;  Age group: Preschool Age (2-5 Yrs), School Age (6-12 Yrs) | Title, Abstract | **149** |
| **13** | “emotion dysregulation” OR “emotion regulation” OR “emotional regulation” OR “affect regulation” OR “self regulation” OR tantrum* OR outburst* OR meltdown OR “emotion coaching” | Methodology: Meta-analysis, systematic review;  Language: English;  Date: Since 2000;  Age group: Preschool Age (2-5 Yrs), School Age (6-12 Yrs) | Full text | **28** |
| **14** | ti,ab(“emotion dysregulation” OR “emotion regulation” OR “emotional regulation” OR “affect regulation” OR “self regulation” OR tantrum* OR outburst* OR meltdown OR “emotion coaching”) | Methodology: Meta-analysis, systematic review;  Language: English;  Date: Since 2000;  Age group: Preschool Age (2-5 Yrs), School Age (6-12 Yrs) | Title, Abstract | **25** |
| **15** | “social skill*” OR “friendship problem*” OR bully* OR bulli* OR “interpersonal problem*” OR “social problem*” OR “peer problem*” OR “peer relation*” OR “social relation*” OR connection OR “peer skills” OR “prosocial” | Methodology: Meta-analysis, systematic review;  Language: English;  Date: Since 2000;  Age group: Preschool Age (2-5 Yrs), School Age (6-12 Yrs) | Full text | **110** |
| **16** | ti,ab(“social skill*” OR “friendship problem*” OR bully* OR bulli* OR “interpersonal problem*” OR “social problem*” OR “peer problem*” OR “peer relation*” OR “social relation*” OR connection OR “peer skills” OR “prosocial”) | Methodology: Meta-analysis, systematic review;  Language: English;  Date: Since 2000;  Age group: Preschool Age (2-5 Yrs), School Age (6-12 Yrs) | Title, Abstract | **82** |
| **17** | Attachment OR “parent-child relationship*” OR “parent-child connection*” OR “attachment style*” OR “reactive attachment” OR “secure attachment” OR “insecure attachment” OR “avoidant attachment” OR “attachment rupture” OR “dismissive attachment” OR “disorganised attachment” OR “disorganized attachment” | Methodology: Meta-analysis, systematic review;  Language: English;  Date: Since 2000;  Age group: Preschool Age (2-5 Yrs), School Age (6-12 Yrs) | Full text | **34** |
| **18** | ti,ab(Attachment OR “parent-child relationship*” OR “parent-child connection*” OR “attachment style*” OR “reactive attachment” OR “secure attachment” OR “insecure attachment” OR “avoidant attachment” OR “attachment rupture” OR “dismissive attachment” OR “disorganised attachment” OR “disorganized attachment”) | Methodology: Meta-analysis, systematic review;  Language: English;  Date: Since 2000;  Age group: Preschool Age (2-5 Yrs), School Age (6-12 Yrs) | Title, Abstract | **31** |
| **19** | Trauma OR PTSD OR “post-traumatic stress disorder” OR “posttraumatic stress disorder” OR “trauma-informed” OR trauma OR stress OR “adverse child experience*” OR divorce OR war OR assault* OR abuse* OR neglect OR “household dysfunction” OR “physical abuse” OR “emotional abuse” OR “sexual abuse” OR “physical neglect” OR “emotional neglect” OR “mental illness” OR “incarcerated relative” OR “mother treated violently” OR “domestic violence” OR “substance abuse” | Methodology: Meta-analysis, systematic review;  Language: English;  Date: Since 2000;  Age group: Preschool Age (2-5 Yrs), School Age (6-12 Yrs) | Full text | **154** |
| **20** | ti,ab(Trauma OR PTSD OR “post-traumatic stress disorder” OR “posttraumatic stress disorder” OR “trauma-informed” OR trauma OR stress OR “adverse child experience*” OR divorce OR war OR assault* OR abuse* OR neglect OR “household dysfunction” OR “physical abuse” OR “emotional abuse” OR “sexual abuse” OR “physical neglect” OR “emotional neglect” OR “mental illness” OR “incarcerated relative” OR “mother treated violently” OR “domestic violence” OR “substance abuse”) | Methodology: Meta-analysis, systematic review;  Language: English;  Date: Since 2000;  Age group: Preschool Age (2-5 Yrs), School Age (6-12 Yrs) | Title, Abstract | **95** |
| **21** | “sensory-regulation” OR “sensory processing difficult*” OR “sensory processing disorder*” | Methodology: Meta-analysis, systematic review;  Language: English;  Date: Since 2000;  Age group: Preschool Age (2-5 Yrs), School Age (6-12 Yrs) | Full text | **0** |
| **22** | ti,ab(“sensory-regulation” OR “sensory processing difficult*” OR “sensory processing disorder*”) | Methodology: Meta-analysis, systematic review;  Language: English;  Date: Since 2000;  Age group: Preschool Age (2-5 Yrs), School Age (6-12 Yrs) | Title, Abstract | **0** |
| **23** | “physical wellbeing” OR “physical well being” | Methodology: Meta-analysis, systematic review;  Language: English;  Date: Since 2000;  Age group: Preschool Age (2-5 Yrs), School Age (6-12 Yrs) | Full text | **1** |
| **24** | ti,ab(“physical wellbeing” OR “physical well being”) | Methodology: Meta-analysis, systematic review;  Language: English;  Date: Since 2000;  Age group: Preschool Age (2-5 Yrs), School Age (6-12 Yrs) | Title, Abstract | **1** |
| **25** | Indigenous OR aboriginal OR “Torres Strait Islander” | Methodology: Meta-analysis, systematic review;  Language: English;  Date: Since 2000;  Age group: Preschool Age (2-5 Yrs), School Age (6-12 Yrs) | Full text | **5** |
| **26** | ti,ab(Indigenous OR aboriginal OR “Torres Strait Islander”) | Methodology: Meta-analysis, systematic review;  Language: English;  Date: Since 2000;  Age group: Preschool Age (2-5 Yrs), School Age (6-12 Yrs) | Title, Abstract | **5** |
| **27** | 2 AND 4 |  |  | **81** |
| **28** | 2 AND 6 |  |  | **218** |
| **29** | 2 AND 8 |  |  | **93** |
| **30** | 2 AND 10 |  |  | **116** |
| **31** | 2 AND 12 |  |  | **84** |
| **32** | 2 AND 14 |  |  | **17** |
| **33** | 2 AND 16 |  |  | **58** |
| **34** | 2 AND 18 |  |  | **14** |
| **35** | 2 AND 20 |  |  | **55** |
| **36** | 2 AND 22 |  |  | **0** |
| **37** | 2 AND 24 |  |  | **1** |
| **38** | 2 AND 26 |  |  | **2** |
| **39** | 27 OR 28 OR 29 OR 30 OR 31 OE 32 OR 33 OR 34 OR 35 OR 36 OR 37 OR 38 |  |  | **426** |
| **PUBMED** | | | | |
| **Search #** | **Key words** | **Restrictions** | **Search Field** | **# of Results** |
| **1** | “family-focused intervent*” OR “early intervent*” OR prevent* OR transdiagnostic OR “targeted group” OR “group therap*” OR “peer group” OR “peer support” OR “targeted therap*” OR “targeted intervent*” OR “indicated intervent*” OR intervent* OR treat* OR “child intervent*” OR “school-based intervent*” OR “parent-child intervent*” OR “teacher-led program*” OR “teacher-led intervent*” OR “parent-led program*” OR “parent-led intervent*” OR “clinician-led program*” OR “clinician-led intervent*” | Methodology: Meta-analysis, systematic review;  Language: English;  Date: Since 2000;  Age group: Preschool Child: 2-5 years, Child: 6-12 years | Full text | **11,740** |
| **2** | “family-focused intervent*” OR “early intervent*” OR prevent* OR transdiagnostic OR “targeted group” OR “group therap*” OR “peer group” OR “peer support” OR “targeted therap*” OR “targeted intervent*” OR “indicated intervent*” OR intervent* OR treat* OR “child intervent*” OR “school-based intervent*” OR “parent-child intervent*” OR “teacher-led program*” OR “teacher-led intervent*” OR “parent-led program*” OR “parent-led intervent*” OR “clinician-led program*” OR “clinician-led intervent*” | Methodology: Meta-analysis, systematic review;  Language: English;  Date: Since 2000;  Age group: Preschool Child: 2-5 years, Child: 6-12 years | Title, Abstract | **10,426** |
| **3** | “mental health” OR “behavioural problem*” OR “behavioral problem*” OR “emotional problem*” OR “social problem*” | Methodology: Meta-analysis, systematic review;  Language: English;  Date: Since 2000;  Age group: Preschool Child: 2-5 years, Child: 6-12 years | Full text | **1177** |
| **4** | “mental health” OR “behavioural problem*” OR “behavioral problem*” OR “emotional problem*” OR “social problem*” | Methodology: Meta-analysis, systematic review;  Language: English;  Date: Since 2000;  Age group: Preschool Child: 2-5 years, Child: 6-12 years | Title, Abstract | **745** |
| **5** | depress* OR mood OR internalizing OR internalizing OR sad* OR low OR unhapp* | Methodology: Meta-analysis, systematic review;  Language: English;  Date: Since 2000;  Age group: Preschool Child: 2-5 years, Child: 6-12 years | Full text | **4982** |
| **6** | depress* OR mood OR internalizing OR internalizing OR sad* OR low OR unhapp* | Methodology: Meta-analysis, systematic review;  Language: English;  Date: Since 2000;  Age group: Preschool Child: 2-5 years, Child: 6-12 years | Title, Abstract | **4396** |
| **7** | Anxi* OR internalizing OR internalizing OR worr* | Methodology: Meta-analysis, systematic review;  Language: English;  Date: Since 2000;  Age group: Preschool Child: 2-5 years, Child: 6-12 years | Full text | **896** |
| **8** | Anxi* OR internalizing OR internalizing OR worr* | Methodology: Meta-analysis, systematic review;  Language: English;  Date: Since 2000;  Age group: Preschool Child: 2-5 years, Child: 6-12 years | Title, Abstract | **834** |
| **9** | Conduct OR CD OR aggress* OR “oppositional defiant disorder*” OR “oppositional defiant” OR oppositional OR ODD OR externalizing OR externalizing OR misbehaviour OR misbehavior OR fight* OR cheating OR violen* OR defian* OR ang* OR argu* | Methodology: Meta-analysis, systematic review;  Language: English;  Date: Since 2000;  Age group: Preschool Child: 2-5 years, Child: 6-12 years | Full text | **8569** |
| **10** | Conduct OR CD OR aggress* OR “oppositional defiant disorder*” OR “oppositional defiant” OR oppositional OR ODD OR externalizing OR externalizing OR misbehaviour OR misbehavior OR fight* OR cheating OR violen* OR defian* OR ang* OR argu* | Methodology: Meta-analysis, systematic review;  Language: English;  Date: Since 2000;  Age group: Preschool Child: 2-5 years, Child: 6-12 years | Title, Abstract | **1489** |
| **11** | ADHD OR attention OR “attention deficit” OR hyper* OR concentrat* | Methodology: Meta-analysis, systematic review;  Language: English;  Date: Since 2000;  Age group: Preschool Child: 2-5 years, Child: 6-12 years | Full text | **2451** |
| **12** | ADHD OR attention OR “attention deficit” OR hyper* OR concentrat* | Methodology: Meta-analysis, systematic review;  Language: English;  Date: Since 2000;  Age group: Preschool Child: 2-5 years, Child: 6-12 years | Title, Abstract | **2180** |
| **13** | “emotion dysregulation” OR “emotion regulation” OR “emotional regulation” OR “affect regulation” OR “self regulation” OR tantrum* OR outburst* OR meltdown OR “emotion coaching” | Methodology: Meta-analysis, systematic review;  Language: English;  Date: Since 2000;  Age group: Preschool Child: 2-5 years, Child: 6-12 years | Full text | **85** |
| **14** | “emotion dysregulation” OR “emotion regulation” OR “emotional regulation” OR “affect regulation” OR “self regulation” OR tantrum* OR outburst* OR meltdown OR “emotion coaching” | Methodology: Meta-analysis, systematic review;  Language: English;  Date: Since 2000;  Age group: Preschool Child: 2-5 years, Child: 6-12 years | Title, Abstract | **79** |
| **15** | “social skill*” OR “friendship problem*” OR bully* OR bulli* OR “interpersonal problem*” OR “social problem*” OR “peer problem*” OR “peer relation*” OR “social relation*” OR connection OR “peer skills” OR “prosocial” | Methodology: Meta-analysis, systematic review;  Language: English;  Date: Since 2000;  Age group: Preschool Child: 2-5 years, Child: 6-12 years | Full text | **422** |
| **16** | “social skill*” OR “friendship problem*” OR bully* OR bulli* OR “interpersonal problem*” OR “social problem*” OR “peer problem*” OR “peer relation*” OR “social relation*” OR connection OR “peer skills” OR “prosocial” | Methodology: Meta-analysis, systematic review;  Language: English;  Date: Since 2000;  Age group: Preschool Child: 2-5 years, Child: 6-12 years | Title, Abstract | **278** |
| **17** | Attachment OR “parent-child relationship*” OR “parent-child connection*” OR “attachment style*” OR “reactive attachment” OR “secure attachment” OR “insecure attachment” OR “avoidant attachment” OR “attachment rupture” OR “dismissive attachment” OR “disorganised attachment” OR “disorganized attachment” | Methodology: Meta-analysis, systematic review;  Language: English;  Date: Since 2000;  Age group: Preschool Child: 2-5 years, Child: 6-12 years | Full text | **136** |
| **18** | Attachment OR “parent-child relationship*” OR “parent-child connection*” OR “attachment style*” OR “reactive attachment” OR “secure attachment” OR “insecure attachment” OR “avoidant attachment” OR “attachment rupture” OR “dismissive attachment” OR “disorganised attachment” OR “disorganized attachment” | Methodology: Meta-analysis, systematic review;  Language: English;  Date: Since 2000;  Age group: Preschool Child: 2-5 years, Child: 6-12 years | Title, Abstract | **108** |
| **19** | Trauma OR PTSD OR “post-traumatic stress disorder” OR “posttraumatic stress disorder” OR “trauma-informed” OR trauma OR stress OR “adverse child experience*” OR divorce OR war OR assault* OR abuse* OR neglect OR “household dysfunction” OR “physical abuse” OR “emotional abuse” OR “sexual abuse” OR “physical neglect” OR “emotional neglect” OR “mental illness” OR “incarcerated relative” OR “mother treated violently” OR “domestic violence” OR “substance abuse” | Methodology: Meta-analysis, systematic review;  Language: English;  Date: Since 2000;  Age group: Preschool Child: 2-5 years, Child: 6-12 years | Full text | **2220** |
| **20** | Trauma OR PTSD OR “post-traumatic stress disorder” OR “posttraumatic stress disorder” OR “trauma-informed” OR trauma OR stress OR “adverse child experience*” OR divorce OR war OR assault* OR abuse* OR neglect OR “household dysfunction” OR “physical abuse” OR “emotional abuse” OR “sexual abuse” OR “physical neglect” OR “emotional neglect” OR “mental illness” OR “incarcerated relative” OR “mother treated violently” OR “domestic violence” OR “substance abuse” | Methodology: Meta-analysis, systematic review;  Language: English;  Date: Since 2000;  Age group: Preschool Child: 2-5 years, Child: 6-12 years | Title, Abstract | **1140** |
| **21** | “sensory-regulation” OR “sensory processing difficult*” OR “sensory processing disorder*” | Methodology: Meta-analysis, systematic review;  Language: English;  Date: Since 2000;  Age group: Preschool Child: 2-5 years, Child: 6-12 years | Full text | **3** |
| **22** | “sensory-regulation” OR “sensory processing difficult*” OR “sensory processing disorder*” | Methodology: Meta-analysis, systematic review;  Language: English;  Date: Since 2000;  Age group: Preschool Child: 2-5 years, Child: 6-12 years | Title, Abstract | **3** |
| **23** | “physical wellbeing” OR “physical well being” | Methodology: Meta-analysis, systematic review;  Language: English;  Date: Since 2000;  Age group: Preschool Child: 2-5 years, Child: 6-12 years | Full text | **11** |
| **24** | “physical wellbeing” OR “physical well-being” | Methodology: Meta-analysis, systematic review;  Language: English;  Date: Since 2000;  Age group: Preschool Child: 2-5 years, Child: 6-12 years | Title, Abstract | **11** |
| **25** | Indigenous OR aboriginal OR “Torres Strait Islander” | Methodology: Meta-analysis, systematic review;  Language: English;  Date: Since 2000;  Age group: Preschool Child: 2-5 years, Child: 6-12 years | Full text | **67** |
| **26** | Indigenous OR aboriginal OR “Torres Strait Islander” | Methodology: Meta-analysis, systematic review;  Language: English;  Date: Since 2000;  Age group: Preschool Child: 2-5 years, Child: 6-12 years | Title, Abstract | **56** |
| **27** | 2 AND 4 |  |  | **469** |
| **28** | 2 AND 6 |  |  | **2967** |
| **29** | 2 AND 8 |  |  | **556** |
| **30** | 2 AND 10 |  |  | **865** |
| **31** | 2 AND 12 |  |  | **1274** |
| **32** | 2 AND 14 |  |  | **48** |
| **33** | 2 AND 16 |  |  | **169** |
| **34** | 2 AND 18 |  |  | **45** |
| **35** | 2 AND 20 |  |  | **648** |
| **36** | 2 AND 22 |  |  | **3** |
| **37** | 2 AND 24 |  |  | **9** |
| **38** | 2 AND 26 |  |  | **36** |
| **39** | 27 OR 28 OR 29 OR 30 OR 31 OE 32 OR 33 OR 34 OR 35 OR 36 OR 37 OR 38 |  |  | **4929** |
| **COCHRANE** | | | | |
| **Search #** | **Key words** | **Restrictions** | **Search Field** | **# of Results** |
| **1** | “family focused intervent*” OR “early intervent*” OR prevent* OR transdiagnostic OR “targeted group” OR “group therap*” OR “peer group” OR “peer support” OR “targeted therap*” OR “targeted intervent*” OR “indicated intervent*” OR intervent* OR treat* OR “child intervent*” OR “school based intervent*” OR “parent child intervent*” OR “teacher led program*” OR “teacher led intervent*” OR “parent led program*” OR “parent led intervent*” OR “clinician led program*” OR “clinician led intervent*” | Content type: Cochrane Reviews;  Date: Since 2000 | Full text | **8450** |
| **2** | “family focused intervent*” OR “early intervent*” OR prevent* OR transdiagnostic OR “targeted group” OR “group therap*” OR “peer group” OR “peer support” OR “targeted therap*” OR “targeted intervent*” OR “indicated intervent*” OR intervent* OR treat* OR “child intervent*” OR “school based intervent*” OR “parent child intervent*” OR “teacher led program*” OR “teacher led intervent*” OR “parent led program*” OR “parent led intervent*” OR “clinician led program*” OR “clinician led intervent*” | Content type: Cochrane Reviews;  Date: Since 2000 | Title, Abstract, Keyword | **8017** |
| **3** | “mental health” OR “behavioural problem*” OR “behavioral problem*” OR “emotional problem*” OR “social problem*” | Content type: Cochrane Reviews;  Date: Since 2000 | Full text | **1454** |
| **4** | “mental health” OR “behavioural problem*” OR “behavioral problem*” OR “emotional problem*” OR “social problem*” | Content type: Cochrane Reviews;  Date: Since 2000 | Title, Abstract, Keyword | **353** |
| **5** | depress* OR mood OR internalizing OR internalizing OR sad* OR low OR unhapp* | Content type: Cochrane Reviews;  Date: Since 2000 | Full text | **8353** |
| **6** | depress* OR mood OR internalizing OR internalizing OR sad* OR low OR unhapp* | Content type: Cochrane Reviews;  Date: Since 2000 | Title, Abstract, Keyword | **5863** |
| **7** | Anxi* OR internalizing OR internalizing OR worr* | Content type: Cochrane Reviews;  Date: Since 2000 | Full text | **8163** |
| **8** | Anxi* OR internalizing OR internalizing OR worr* | Content type: Cochrane Reviews;  Date: Since 2000 | Title, Abstract, Keyword | **2282** |
| **9** | Conduct OR CD OR aggress* OR “oppositional defiant disorder*” OR “oppositional defiant” OR oppositional OR ODD OR externalizing OR externalizing OR misbehaviour OR misbehavior OR fight* OR cheating OR violen* OR defian* OR ang* OR argu* | Content type: Cochrane Reviews;  Date: Since 2000 | Full text | **8367** |
| **10** | Conduct OR CD OR aggress* OR “oppositional defiant disorder*” OR “oppositional defiant” OR oppositional OR ODD OR externalizing OR externalizing OR misbehaviour OR misbehavior OR fight* OR cheating OR violen* OR defian* OR ang* OR argu* | Content type: Cochrane Reviews;  Date: Since 2000 | Title, Abstract, Keyword | **5055** |
| **11** | ADHD OR attention OR “attention deficit” OR hyper* OR concentrat* | Content type: Cochrane Reviews;  Date: Since 2000 | Full text | **5926** |
| **12** | ADHD OR attention OR “attention deficit” OR hyper* OR concentrat* | Content type: Cochrane Reviews;  Date: Since 2000 | Title, Abstract, Keyword | **1588** |
| **13** | “emotion dysregulation” OR “emotion regulation” OR “emotional regulation” OR “affect regulation” OR “self regulation” OR tantrum* OR outburst* OR meltdown OR “emotion coaching” | Content type: Cochrane Reviews;  Date: Since 2000 | Full text | **180** |
| **14** | “emotion dysregulation” OR “emotion regulation” OR “emotional regulation” OR “affect regulation” OR “self regulation” OR tantrum* OR outburst* OR meltdown OR “emotion coaching” | Content type: Cochrane Reviews;  Date: Since 2000 | Title, Abstract, Keyword | **14** |
| **15** | “social skill*” OR “friendship problem*” OR bully* OR bulli* OR “interpersonal problem*” OR “social problem*” OR “peer problem*” OR “peer relation*” OR “social relation*” OR connection OR “peer skills” OR “prosocial” | Content type: Cochrane Reviews;  Date: Since 2000 | Full text | **1374** |
| **16** | “social skill*” OR “friendship problem*” OR bully* OR bulli* OR “interpersonal problem*” OR “social problem*” OR “peer problem*” OR “peer relation*” OR “social relation*” OR connection OR “peer skills” OR “prosocial” | Content type: Cochrane Reviews;  Date: Since 2000 | Title, Abstract, Keyword | **231** |
| **17** | Attachment OR “parent child relationship*” OR “parent child connection*” OR “attachment style*” OR “reactive attachment” OR “secure attachment” OR “insecure attachment” OR “avoidant attachment” OR “attachment rupture” OR “dismissive attachment” OR “disorganised attachment” OR “disorganized attachment” | Content type: Cochrane Reviews;  Date: Since 2000 | Full text | **6735** |
| **18** | Attachment OR “parent child relationship*” OR “parent child connection*” OR “attachment style*” OR “reactive attachment” OR “secure attachment” OR “insecure attachment” OR “avoidant attachment” OR “attachment rupture” OR “dismissive attachment” OR “disorganised attachment” OR “disorganized attachment” | Content type: Cochrane Reviews;  Date: Since 2000 | Title, Abstract, Keyword | **149** |
| **19** | Trauma OR PTSD OR “post traumatic stress disorder” OR “posttraumatic stress disorder” OR “trauma informed” OR trauma OR stress OR “adverse child experience*” OR divorce OR war OR assault* OR abuse* OR neglect OR “household dysfunction” OR “physical abuse” OR “emotional abuse” OR “sexual abuse” OR “physical neglect” OR “emotional neglect” OR “mental illness” OR “incarcerated relative” OR “mother treated violently” OR “domestic violence” OR “substance abuse” | Content type: Cochrane Reviews;  Date: Since 2000 | Full text | **3532** |
| **20** | Trauma OR PTSD OR “post traumatic stress disorder” OR “posttraumatic stress disorder” OR “trauma informed” OR trauma OR stress OR “adverse child experience*” OR divorce OR war OR assault* OR abuse* OR neglect OR “household dysfunction” OR “physical abuse” OR “emotional abuse” OR “sexual abuse” OR “physical neglect” OR “emotional neglect” OR “mental illness” OR “incarcerated relative” OR “mother treated violently” OR “domestic violence” OR “substance abuse” | Content type: Cochrane Reviews;  Date: Since 2000 | Title, Abstract, Keyword | **1029** |
| **21** | “sensory regulation” OR “sensory processing difficult*” OR “sensory processing disorder*” | Content type: Cochrane Reviews;  Date: Since 2000 | Full text | **1** |
| **22** | “sensory regulation” OR “sensory processing difficult*” OR “sensory processing disorder*” | Content type: Cochrane Reviews;  Date: Since 2000 | Title, Abstract, Keyword | **0** |
| **23** | “physical wellbeing” OR “physical well being” | Content type: Cochrane Reviews;  Date: Since 2000 | Full text | **84** |
| **24** | “physical wellbeing” OR “physical well being” | Content type: Cochrane Reviews;  Date: Since 2000 | Title, Abstract, Keyword | **8** |
| **25** | Indigenous OR aboriginal OR “Torres Strait Islander” | Content type: Cochrane Reviews;  Date: Since 2000 | Full text | **158** |
| **26** | Indigenous OR aboriginal OR “Torres Strait Islander” | Content type: Cochrane Reviews;  Date: Since 2000 | Title, Abstract, Keyword | **14** |
| **27** | pediatric* OR paediatric* OR child* OR kindergarten* OR "elementary school*" OR "nursery school*" OR schoolchild* OR youth* | Content type: Cochrane Reviews;  Date: Since 2000 | Full text | **4948** |
| **28** | pediatric* OR paediatric* OR child* OR kindergarten* OR "elementary school*" OR "nursery school*" OR schoolchild* OR youth* | Content type: Cochrane Reviews;  Date: Since 2000 | Title, Abstract, Keyword | **2557** |
| **29** | 2 AND 4 AND 28 |  |  | **109** |
| **30** | 2 AND 6 AND 28 |  |  | **1812** |
| **31** | 2 AND 8 AND 28 |  |  | **768** |
| **32** | 2 AND 10 AND 28 |  |  | **1476** |
| **33** | 2 AND 12 AND 28 |  |  | **560** |
| **34** | 2 AND 14 AND 28 |  |  | **4** |
| **35** | 2 AND 16 AND 28 |  |  | **71** |
| **36** | 2 AND 18 AND 28 |  |  | **51** |
| **37** | 2 AND 20 AND 28 |  |  | **297** |
| **38** | 2 AND 22 AND 28 |  |  | **0** |
| **39** | 2 AND 24 AND 28 |  |  | **0** |
| **40** | 2 AND 26 AND 28 |  |  | **9** |
| **41** | 27 OR 28 OR 29 OR 30 OR 31 OE 32 OR 33 OR 34 OR 35 OR 36 OR 37 OR 38 |  |  | **Couldn’t combine searches in COCHRANE, so S29-S40 downloaded separately** |
| **CINAHL** | | | | |
| **Search #** | **Key words** | **Restrictions** | **Search Field** | **# of Results** |
| **1** | “family-focused intervent*” OR “early intervent*” OR prevent* OR transdiagnostic OR “targeted group” OR “group therap*” OR “peer group” OR “peer support” OR “targeted therap*” OR “targeted intervent*” OR “indicated intervent*” OR intervent* OR treat* OR “child intervent*” OR “school-based intervent*” OR “parent-child intervent*” OR “teacher-led program*” OR “teacher-led intervent*” OR “parent-led program*” OR “parent-led intervent*” OR “clinician-led program*” OR “clinician-led intervent*” | Language: English;  Date: Since 2000;  Age group: Child, Preschool: 2-5 years, Child: 6-12 years;  Publication Type: Meta-Analysis, Systematic Review | Full text | **6504** |
| **2** | “family-focused intervent*” OR “early intervent*” OR prevent* OR transdiagnostic OR “targeted group” OR “group therap*” OR “peer group” OR “peer support” OR “targeted therap*” OR “targeted intervent*” OR “indicated intervent*” OR intervent* OR treat* OR “child intervent*” OR “school-based intervent*” OR “parent-child intervent*” OR “teacher-led program*” OR “teacher-led intervent*” OR “parent-led program*” OR “parent-led intervent*” OR “clinician-led program*” OR “clinician-led intervent*” | Language: English;  Date: Since 2000;  Age group: Child, Preschool: 2-5 years, Child: 6-12 years;  Publication Type: Meta-Analysis, Systematic Review | Title, Abstract | **5496** |
| **3** | “mental health” OR “behavioural problem*” OR “behavioral problem*” OR “emotional problem*” OR “social problem*” | Language: English;  Date: Since 2000;  Age group: Child, Preschool: 2-5 years, Child: 6-12 years;  Publication Type: Meta-Analysis, Systematic Review | Full text | **918** |
| **4** | “mental health” OR “behavioural problem*” OR “behavioral problem*” OR “emotional problem*” OR “social problem*” | Language: English;  Date: Since 2000;  Age group: Child, Preschool: 2-5 years, Child: 6-12 years;  Publication Type: Meta-Analysis, Systematic Review | Title, Abstract | **556** |
| **5** | depress* OR mood OR internalizing OR internalizing OR sad* OR low OR unhapp* | Language: English;  Date: Since 2000;  Age group: Child, Preschool: 2-5 years, Child: 6-12 years;  Publication Type: Meta-Analysis, Systematic Review | Full text | **2009** |
| **6** | depress* OR mood OR internalizing OR internalizing OR sad* OR low OR unhapp* | Language: English;  Date: Since 2000;  Age group: Child, Preschool: 2-5 years, Child: 6-12 years;  Publication Type: Meta-Analysis, Systematic Review | Title, Abstract | **1874** |
| **7** | Anxi* OR internalizing OR internalizing OR worr* | Language: English;  Date: Since 2000;  Age group: Child, Preschool: 2-5 years, Child: 6-12 years;  Publication Type: Meta-Analysis, Systematic Review | Full text | **522** |
| **8** | Anxi* OR internalizing OR internalizing OR worr* | Language: English;  Date: Since 2000;  Age group: Child, Preschool: 2-5 years, Child: 6-12 years;  Publication Type: Meta-Analysis, Systematic Review | Title, Abstract | **464** |
| **9** | Conduct OR CD OR aggress* OR “oppositional defiant disorder*” OR “oppositional defiant” OR oppositional OR ODD OR externalizing OR externalizing OR misbehaviour OR misbehavior OR fight* OR cheating OR violen* OR defian* OR ang* OR argu* | Language: English;  Date: Since 2000;  Age group: Child, Preschool: 2-5 years, Child: 6-12 years;  Publication Type: Meta-Analysis, Systematic Review | Full text | **1984** |
| **10** | Conduct OR CD OR aggress* OR “oppositional defiant disorder*” OR “oppositional defiant” OR oppositional OR ODD OR externalizing OR externalizing OR misbehaviour OR misbehavior OR fight* OR cheating OR violen* OR defian* OR ang* OR argu* | Language: English;  Date: Since 2000;  Age group: Child, Preschool: 2-5 years, Child: 6-12 years;  Publication Type: Meta-Analysis, Systematic Review | Title, Abstract | **1272** |
| **11** | ADHD OR attention OR “attention deficit” OR hyper* OR concentrat* | Language: English;  Date: Since 2000;  Age group: Child, Preschool: 2-5 years, Child: 6-12 years;  Publication Type: Meta-Analysis, Systematic Review | Full text | **1207** |
| **12** | ADHD OR attention OR “attention deficit” OR hyper* OR concentrat* | Language: English;  Date: Since 2000;  Age group: Child, Preschool: 2-5 years, Child: 6-12 years;  Publication Type: Meta-Analysis, Systematic Review | Title, Abstract | **1006** |
| **13** | “emotion dysregulation” OR “emotion regulation” OR “emotional regulation” OR “affect regulation” OR “self regulation” OR tantrum* OR outburst* OR meltdown OR “emotion coaching” | Language: English;  Date: Since 2000;  Age group: Child, Preschool: 2-5 years, Child: 6-12 years;  Publication Type: Meta-Analysis, Systematic Review | Full text | **61** |
| **14** | “emotion dysregulation” OR “emotion regulation” OR “emotional regulation” OR “affect regulation” OR “self regulation” OR tantrum* OR outburst* OR meltdown OR “emotion coaching” | Language: English;  Date: Since 2000;  Age group: Child, Preschool: 2-5 years, Child: 6-12 years;  Publication Type: Meta-Analysis, Systematic Review | Title, Abstract | **48** |
| **15** | “social skill*” OR “friendship problem*” OR bully* OR bulli* OR “interpersonal problem*” OR “social problem*” OR “peer problem*” OR “peer relation*” OR “social relation*” OR connection OR “peer skills” OR “prosocial” | Language: English;  Date: Since 2000;  Age group: Child, Preschool: 2-5 years, Child: 6-12 years;  Publication Type: Meta-Analysis, Systematic Review | Full text | **311** |
| **16** | “social skill*” OR “friendship problem*” OR bully* OR bulli* OR “interpersonal problem*” OR “social problem*” OR “peer problem*” OR “peer relation*” OR “social relation*” OR connection OR “peer skills” OR “prosocial” | Language: English;  Date: Since 2000;  Age group: Child, Preschool: 2-5 years, Child: 6-12 years;  Publication Type: Meta-Analysis, Systematic Review | Title, Abstract | **231** |
| **17** | Attachment OR “parent-child relationship*” OR “parent-child connection*” OR “attachment style*” OR “reactive attachment” OR “secure attachment” OR “insecure attachment” OR “avoidant attachment” OR “attachment rupture” OR “dismissive attachment” OR “disorganised attachment” OR “disorganized attachment” | Language: English;  Date: Since 2000;  Age group: Child, Preschool: 2-5 years, Child: 6-12 years;  Publication Type: Meta-Analysis, Systematic Review | Full text | **96** |
| **18** | Attachment OR “parent-child relationship*” OR “parent-child connection*” OR “attachment style*” OR “reactive attachment” OR “secure attachment” OR “insecure attachment” OR “avoidant attachment” OR “attachment rupture” OR “dismissive attachment” OR “disorganised attachment” OR “disorganized attachment” | Language: English;  Date: Since 2000;  Age group: Child, Preschool: 2-5 years, Child: 6-12 years;  Publication Type: Meta-Analysis, Systematic Review | Title, Abstract | **84** |
| **19** | Trauma OR PTSD OR “post-traumatic stress disorder” OR “posttraumatic stress disorder” OR “trauma-informed” OR trauma OR stress OR “adverse child experience*” OR divorce OR war OR assault* OR abuse* OR neglect OR “household dysfunction” OR “physical abuse” OR “emotional abuse” OR “sexual abuse” OR “physical neglect” OR “emotional neglect” OR “mental illness” OR “incarcerated relative” OR “mother treated violently” OR “domestic violence” OR “substance abuse” | Language: English;  Date: Since 2000;  Age group: Child, Preschool: 2-5 years, Child: 6-12 years;  Publication Type: Meta-Analysis, Systematic Review | Full text | **1199** |
| **20** | Trauma OR PTSD OR “post-traumatic stress disorder” OR “posttraumatic stress disorder” OR “trauma-informed” OR trauma OR stress OR “adverse child experience*” OR divorce OR war OR assault* OR abuse* OR neglect OR “household dysfunction” OR “physical abuse” OR “emotional abuse” OR “sexual abuse” OR “physical neglect” OR “emotional neglect” OR “mental illness” OR “incarcerated relative” OR “mother treated violently” OR “domestic violence” OR “substance abuse” | Language: English;  Date: Since 2000;  Age group: Child, Preschool: 2-5 years, Child: 6-12 years;  Publication Type: Meta-Analysis, Systematic Review | Title, Abstract | **780** |
| **21** | “sensory-regulation” OR “sensory processing difficult*” OR “sensory processing disorder*” | Language: English;  Date: Since 2000;  Age group: Child, Preschool: 2-5 years, Child: 6-12 years;  Publication Type: Meta-Analysis, Systematic Review | Full text | **1** |
| **22** | “sensory-regulation” OR “sensory processing difficult*” OR “sensory processing disorder*” | Language: English;  Date: Since 2000;  Age group: Child, Preschool: 2-5 years, Child: 6-12 years;  Publication Type: Meta-Analysis, Systematic Review | Title, Abstract | **1** |
| **23** | “physical wellbeing” OR “physical well being” | Language: English;  Date: Since 2000;  Age group: Child, Preschool: 2-5 years, Child: 6-12 years;  Publication Type: Meta-Analysis, Systematic Review | Full text | **7** |
| **24** | “physical wellbeing” OR “physical well being” | Language: English;  Date: Since 2000;  Age group: Child, Preschool: 2-5 years, Child: 6-12 years;  Publication Type: Meta-Analysis, Systematic Review | Title, Abstract | **7** |
| **25** | Indigenous OR aboriginal OR “Torres Strait Islander” | Language: English;  Date: Since 2000;  Age group: Child, Preschool: 2-5 years, Child: 6-12 years;  Publication Type: Meta-Analysis, Systematic Review | Full text | **65** |
| **26** | Indigenous OR aboriginal OR “Torres Strait Islander” | Language: English;  Date: Since 2000;  Age group: Child, Preschool: 2-5 years, Child: 6-12 years;  Publication Type: Meta-Analysis, Systematic Review | Title, Abstract | **49** |
| **27** | 2 AND 4 |  |  | **333** |
| **28** | 2 AND 6 |  |  | **1156** |
| **29** | 2 AND 8 |  |  | **300** |
| **30** | 2 AND 10 |  |  | **719** |
| **31** | 2 AND 12 |  |  | **606** |
| **32** | 2 AND 14 |  |  | **32** |
| **33** | 2 AND 16 |  |  | **131** |
| **34** | 2 AND 18 |  |  | **43** |
| **35** | 2 AND 20 |  |  | **430** |
| **36** | 2 AND 22 |  |  | **0** |
| **37** | 2 AND 24 |  |  | **7** |
| **38** | 2 AND 26 |  |  | **29** |
| **39** | 27 OR 28 OR 29 OR 30 OR 31 OE 32 OR 33 OR 34 OR 35 OR 36 OR 37 OR 38 |  |  | **2561** |
| **ERIC (ProQuest Education Databases)** | | | | |
| **Search #** | **Key words** | **Restrictions** | **Search Field** | **# of Results** |
| **1** | (“family-focused intervent*” OR “early intervent*” OR prevent* OR transdiagnostic OR “targeted group” OR “group therap*” OR “peer group” OR “peer support” OR “targeted therap*” OR “targeted intervent*” OR “indicated intervent*” OR intervent* OR treat* OR “child intervent*” OR “school-based intervent*” OR “parent-child intervent*” OR “teacher-led program*” OR “teacher-led intervent*” OR “parent-led program*” OR “parent-led intervent*” OR “clinician-led program*” OR “clinician-led intervent*”) AND (pediatric* OR paediatric* OR child* OR kindergarten* OR “elementary school*” OR “nursery school*” OR schoolchild* OR youth*) AND (“meta-analys*” OR “systematic review*”) | Language: English;  Date: Since 2000; | Full text | **1147** |
| **2** | ti,ab(“family-focused intervent*” OR “early intervent*” OR prevent* OR transdiagnostic OR “targeted group” OR “group therap*” OR “peer group” OR “peer support” OR “targeted therap*” OR “targeted intervent*” OR “indicated intervent*” OR intervent* OR treat* OR “child intervent*” OR “school-based intervent*” OR “parent-child intervent*” OR “teacher-led program*” OR “teacher-led intervent*” OR “parent-led program*” OR “parent-led intervent*” OR “clinician-led program*” OR “clinician-led intervent*”) AND ti,ab(pediatric* OR paediatric* OR child* OR kindergarten* OR “elementary school*” OR “nursery school*” OR schoolchild* OR youth*) AND ti,ab(“meta-analys*” OR “systematic review*”) | Language: English;  Date: Since 2000; | Title, Abstract | **774** |
| **3** | ("mental health" OR "behavioural problem*" OR "behavioral problem*" OR "emotional problem*" OR "social problem*") AND (pediatric* OR paediatric* OR child* OR kindergarten* OR "elementary school*" OR "nursery school*" OR schoolchild* OR youth*) AND ("meta-analys*" OR "systematic review*") | Language: English;  Date: Since 2000; | Full text | **151** |
| **4** | ti,ab("mental health" OR "behavioural problem*" OR "behavioral problem*" OR "emotional problem*" OR "social problem*") AND ti,ab(pediatric* OR paediatric* OR child* OR kindergarten* OR "elementary school*" OR "nursery school*" OR schoolchild* OR youth*) AND ti,ab("meta-analys*" OR "systematic review*") | Language: English;  Date: Since 2000 | Title, Abstract | **91** |
| **5** | (depress* OR mood OR internalizing OR internalizing OR sad* OR low OR unhapp*) AND (pediatric* OR paediatric* OR child* OR kindergarten* OR "elementary school*" OR "nursery school*" OR schoolchild* OR youth*) AND ("meta-analys*" OR "systematic review*") | Language: English;  Date: Since 2000 | Full text | **426** |
| **6** | ti,ab(depress* OR mood OR internalizing OR internalizing OR sad* OR low OR unhapp*) AND ti,ab(pediatric* OR paediatric* OR child* OR kindergarten* OR "elementary school*" OR "nursery school*" OR schoolchild* OR youth*) AND ti,ab("meta-analys*" OR "systematic review*") | Language: English;  Date: Since 2000 | Title, Abstract | **297** |
| **7** | (Anxi* OR internalizing OR internalizing OR worr*) AND (pediatric* OR paediatric* OR child* OR kindergarten* OR "elementary school*" OR "nursery school*" OR schoolchild* OR youth*) AND ("meta-analys*" OR "systematic review*") | Language: English;  Date: Since 2000 | Full text | **129** |
| **8** | ti,ab(Anxi* OR internalizing OR internalizing OR worr*) AND ti,ab(pediatric* OR paediatric* OR child* OR kindergarten* OR "elementary school*" OR "nursery school*" OR schoolchild* OR youth*) AND ti,ab("meta-analys*" OR "systematic review*") | Language: English;  Date: Since 2000 | Title, Abstract | **99** |
| **9** | Conduct OR CD OR aggress* OR “oppositional defiant disorder*” OR “oppositional defiant” OR oppositional OR ODD OR externalizing OR externalizing OR misbehaviour OR misbehavior OR fight* OR cheating OR violen* OR defian* OR ang* OR argu* | Language: English;  Date: Since 2000 | Full text | **311** |
| **10** | ti,ab(Conduct OR CD OR aggress* OR "oppositional defiant disorder*" OR "oppositional defiant" OR oppositional OR ODD OR externalizing OR externalizing OR misbehaviour OR misbehavior OR fight* OR cheating OR violen* OR defian* OR ang* OR argu*) AND ti,ab(pediatric* OR paediatric* OR child* OR kindergarten* OR "elementary school*" OR "nursery school*" OR schoolchild* OR youth*) AND ti,ab("meta-analys*" OR "systematic review*") | Language: English;  Date: Since 2000 | Title, Abstract | **197** |
| **11** | (ADHD OR attention OR "attention deficit" OR hyper* OR concentrat*) AND (pediatric* OR paediatric* OR child* OR kindergarten* OR "elementary school*" OR "nursery school*" OR schoolchild* OR youth*) AND ("meta-analys*" OR "systematic review*") | Language: English;  Date: Since 2000 | Full text | **266** |
| **12** | ti,ab(ADHD OR attention OR "attention deficit" OR hyper* OR concentrat*) AND ti,ab(pediatric* OR paediatric* OR child* OR kindergarten* OR "elementary school*" OR "nursery school*" OR schoolchild* OR youth*) AND ti,ab("meta-analys*" OR "systematic review*") | Language: English;  Date: Since 2000 | Title, Abstract | **151** |
| **13** | ("emotion dysregulation" OR "emotion regulation" OR "emotional regulation" OR "affect regulation" OR "self regulation" OR tantrum* OR outburst* OR meltdown OR "emotion coaching") AND (pediatric* OR paediatric* OR child* OR kindergarten* OR "elementary school*" OR "nursery school*" OR schoolchild* OR youth*) AND ("meta-analys*" OR "systematic review*") | Language: English;  Date: Since 2000 | Full text | **29** |
| **14** | ti,ab("emotion dysregulation" OR "emotion regulation" OR "emotional regulation" OR "affect regulation" OR "self regulation" OR tantrum* OR outburst* OR meltdown OR "emotion coaching") AND ti,ab(pediatric* OR paediatric* OR child* OR kindergarten* OR "elementary school*" OR "nursery school*" OR schoolchild* OR youth*) AND ti,ab("meta-analys*" OR "systematic review*") | Language: English;  Date: Since 2000 | Title, Abstract | **23** |
| **15** | ("social skill*" OR "friendship problem*" OR bully* OR bulli* OR "interpersonal problem*" OR "social problem*" OR "peer problem*" OR "peer relation*" OR "social relation*" OR connection OR "peer skills" OR "prosocial") AND (pediatric* OR paediatric* OR child* OR kindergarten* OR "elementary school*" OR "nursery school*" OR schoolchild* OR youth*) AND ("meta-analys*" OR "systematic review*") | Language: English;  Date: Since 2000 | Full text | **179** |
| **16** | ti,ab("social skill*" OR "friendship problem*" OR bully* OR bulli* OR "interpersonal problem*" OR "social problem*" OR "peer problem*" OR "peer relation*" OR "social relation*" OR connection OR "peer skills" OR "prosocial") AND ti,ab(pediatric* OR paediatric* OR child* OR kindergarten* OR "elementary school*" OR "nursery school*" OR schoolchild* OR youth*) AND ti,ab("meta-analys*" OR "systematic review*") | Language: English;  Date: Since 2000 | Title, Abstract | **105** |
| **17** | (Attachment OR "parent-child relationship*" OR "parent-child connection*" OR "attachment style*" OR "reactive attachment" OR "secure attachment" OR "insecure attachment" OR "avoidant attachment" OR "attachment rupture" OR "dismissive attachment" OR "disorganised attachment" OR "disorganized attachment") AND (pediatric* OR paediatric* OR child* OR kindergarten* OR "elementary school*" OR "nursery school*" OR schoolchild* OR youth*) AND ("meta-analys*" OR "systematic review*") | Language: English;  Date: Since 2000 | Full text | **144** |
| **18** | ti,ab(Attachment OR "parent-child relationship*" OR "parent-child connection*" OR "attachment style*" OR "reactive attachment" OR "secure attachment" OR "insecure attachment" OR "avoidant attachment" OR "attachment rupture" OR "dismissive attachment" OR "disorganised attachment" OR "disorganized attachment") AND ti,ab(pediatric* OR paediatric* OR child* OR kindergarten* OR "elementary school*" OR "nursery school*" OR schoolchild* OR youth*) AND ti,ab("meta-analys*" OR "systematic review*") | Language: English;  Date: Since 2000 | Title, Abstract | **25** |
| **19** | (Trauma OR PTSD OR "post-traumatic stress disorder" OR "posttraumatic stress disorder" OR "trauma-informed" OR trauma OR stress OR "adverse child experience*" OR divorce OR war OR assault* OR abuse* OR neglect OR "household dysfunction" OR "physical abuse" OR "emotional abuse" OR "sexual abuse" OR "physical neglect" OR "emotional neglect" OR "mental illness" OR "incarcerated relative" OR "mother treated violently" OR "domestic violence" OR "substance abuse") AND (pediatric* OR paediatric* OR child* OR kindergarten* OR "elementary school*" OR "nursery school*" OR schoolchild* OR youth*) AND ("meta-analys*" OR "systematic review*") | Language: English;  Date: Since 2000 | Full text | **188** |
| **20** | ti,ab(Trauma OR PTSD OR "post-traumatic stress disorder" OR "posttraumatic stress disorder" OR "trauma-informed" OR trauma OR stress OR "adverse child experience*" OR divorce OR war OR assault* OR abuse* OR neglect OR "household dysfunction" OR "physical abuse" OR "emotional abuse" OR "sexual abuse" OR "physical neglect" OR "emotional neglect" OR "mental illness" OR "incarcerated relative" OR "mother treated violently" OR "domestic violence" OR "substance abuse") AND ti,ab(pediatric* OR paediatric* OR child* OR kindergarten* OR "elementary school*" OR "nursery school*" OR schoolchild* OR youth*) AND ti,ab("meta-analys*" OR "systematic review*") | Language: English;  Date: Since 2000 | Title, Abstract | **103** |
| **21** | ("sensory-regulation" OR "sensory processing difficult*" OR "sensory processing disorder*") AND (pediatric* OR paediatric* OR child* OR kindergarten* OR "elementary school*" OR "nursery school*" OR schoolchild* OR youth*) AND ("meta-analys*" OR "systematic review*") | Language: English;  Date: Since 2000 | Full text | **0** |
| **22** | ti,ab("sensory-regulation" OR "sensory processing difficult*" OR "sensory processing disorder*") AND ti,ab(pediatric* OR paediatric* OR child* OR kindergarten* OR "elementary school*" OR "nursery school*" OR schoolchild* OR youth*) AND ti,ab("meta-analys*" OR "systematic review*") | Language: English;  Date: Since 2000 | Title, Abstract | **0** |
| **23** | ("physical wellbeing" OR "physical well being") AND (pediatric* OR paediatric* OR child* OR kindergarten* OR "elementary school*" OR "nursery school*" OR schoolchild* OR youth*) AND ("meta-analys*" OR "systematic review*") | Language: English;  Date: Since 2000 | Full text | **1** |
| **24** | ti,ab("physical wellbeing" OR "physical well being") AND ti,ab(pediatric* OR paediatric* OR child* OR kindergarten* OR "elementary school*" OR "nursery school*" OR schoolchild* OR youth*) AND ti,ab("meta-analys*" OR "systematic review*") | Language: English;  Date: Since 2000 | Title, Abstract | **0** |
| **25** | (Indigenous OR aboriginal OR "Torres Strait Islander") AND (pediatric* OR paediatric* OR child* OR kindergarten* OR "elementary school*" OR "nursery school*" OR schoolchild* OR youth*) AND ("meta-analys*" OR "systematic review*") | Language: English;  Date: Since 2000 | Full text | **13** |
| **26** | ti,ab(Indigenous OR aboriginal OR "Torres Strait Islander") AND ti,ab(pediatric* OR paediatric* OR child* OR kindergarten* OR "elementary school*" OR "nursery school*" OR schoolchild* OR youth*) AND ti,ab("meta-analys*" OR "systematic review*") | Language: English;  Date: Since 2000 | Title, Abstract | **10** |
| **27** | 2 AND 4 |  |  | **57** |
| **28** | 2 AND 6 |  |  | **170** |
| **29** | 2 AND 8 |  |  | **56** |
| **30** | 2 AND 10 |  |  | **109** |
| **31** | 2 AND 12 |  |  | **78** |
| **32** | 2 AND 14 |  |  | **16** |
| **33** | 2 AND 16 |  |  | **74** |
| **34** | 2 AND 18 |  |  | **10** |
| **35** | 2 AND 20 |  |  | **66** |
| **36** | 2 AND 22 |  |  | **0** |
| **37** | 2 AND 24 |  |  | **0** |
| **38** | 2 AND 26 |  |  | **4** |
| **39** | 27 OR 28 OR 29 OR 30 OR 31 OR 32 OR 33 OR 34 OR 35 OR 36 OR 37 OR 38 |  |  | **411** |
| **FAMILY AND SOCIETY STUDIES WORLDWIDE** | | | | |
| **Search #** | **Key words** | **Restrictions** | **Search Field** | **# of Results** |
| **1** | “family-focused intervent*” OR “early intervent*” OR prevent* OR transdiagnostic OR “targeted group” OR “group therap*” OR “peer group” OR “peer support” OR “targeted therap*” OR “targeted intervent*” OR “indicated intervent*” OR intervent* OR treat* OR “child intervent*” OR “school-based intervent*” OR “parent-child intervent*” OR “teacher-led program*” OR “teacher-led intervent*” OR “parent-led program*” OR “parent-led intervent*” OR “clinician-led program*” OR “clinician-led intervent*” | Date: Since 2000;  Language: English | Full text | **326,013** |
| **2** | “family-focused intervent*” OR “early intervent*” OR prevent* OR transdiagnostic OR “targeted group” OR “group therap*” OR “peer group” OR “peer support” OR “targeted therap*” OR “targeted intervent*” OR “indicated intervent*” OR intervent* OR treat* OR “child intervent*” OR “school-based intervent*” OR “parent-child intervent*” OR “teacher-led program*” OR “teacher-led intervent*” OR “parent-led program*” OR “parent-led intervent*” OR “clinician-led program*” OR “clinician-led intervent*” | Date: Since 2000;  Language: English | Title, Abstract | **290,011** |
| **3** | “mental health” OR “behavioural problem*” OR “behavioral problem*” OR “emotional problem*” OR “social problem*” | Date: Since 2000;  Language: English | Full text | **57,813** |
| **4** | “mental health” OR “behavioural problem*” OR “behavioral problem*” OR “emotional problem*” OR “social problem*” | Date: Since 2000;  Language: English | Title, Abstract | **45,754** |
| **5** | depress* OR mood OR internalizing OR internalizing OR sad* OR low OR unhapp* | Date: Since 2000;  Language: English | Full text | **158,426** |
| **6** | depress* OR mood OR internalizing OR internalizing OR sad* OR low OR unhapp* | Date: Since 2000;  Language: English | Title, Abstract | **151,048** |
| **7** | Anxi* OR internalizing OR internalizing OR worr* | Date: Since 2000;  Language: English | Full text | **45,854** |
| **8** | Anxi* OR internalizing OR internalizing OR worr* | Date: Since 2000;  Language: English | Title, Abstract | **43,380** |
| **9** | Conduct OR CD OR aggress* OR “oppositional defiant disorder*” OR “oppositional defiant” OR oppositional OR ODD OR externalizing OR externalizing OR misbehaviour OR misbehavior OR fight* OR cheating OR violen* OR defian* OR ang* OR argu* | Date: Since 2000;  Language: English | Full text | **129,290** |
| **10** | Conduct OR CD OR aggress* OR “oppositional defiant disorder*” OR “oppositional defiant” OR oppositional OR ODD OR externalizing OR externalizing OR misbehaviour OR misbehavior OR fight* OR cheating OR violen* OR defian* OR ang* OR argu* | Date: Since 2000;  Language: English | Title, Abstract | **108,758** |
| **11** | ADHD OR attention OR “attention deficit” OR hyper* OR concentrat* | Date: Since 2000;  Language: English | Full text | **88,102** |
| **12** | ADHD OR attention OR “attention deficit” OR hyper* OR concentrat* | Date: Since 2000;  Language: English | Title, Abstract | **84,155** |
| **13** | “emotion dysregulation” OR “emotion regulation” OR “emotional regulation” OR “affect regulation” OR “self regulation” OR tantrum* OR outburst* OR meltdown OR “emotion coaching” | Date: Since 2000;  Language: English | Full text | **5,942** |
| **14** | “emotion dysregulation” OR “emotion regulation” OR “emotional regulation” OR “affect regulation” OR “self regulation” OR tantrum* OR outburst* OR meltdown OR “emotion coaching” | Date: Since 2000;  Language: English | Title, Abstract | **5,322** |
| **15** | “social skill*” OR “friendship problem*” OR bully* OR bulli* OR “interpersonal problem*” OR “social problem*” OR “peer problem*” OR “peer relation*” OR “social relation*” OR connection OR “peer skills” OR “prosocial” | Date: Since 2000;  Language: English | Full text | **24,837** |
| **16** | “social skill*” OR “friendship problem*” OR bully* OR bulli* OR “interpersonal problem*” OR “social problem*” OR “peer problem*” OR “peer relation*” OR “social relation*” OR connection OR “peer skills” OR “prosocial” | Date: Since 2000;  Language: English | Title, Abstract | **19,607** |
| **17** | Attachment OR “parent-child relationship*” OR “parent-child connection*” OR “attachment style*” OR “reactive attachment” OR “secure attachment” OR “insecure attachment” OR “avoidant attachment” OR “attachment rupture” OR “dismissive attachment” OR “disorganised attachment” OR “disorganized attachment” | Date: Since 2000;  Language: English | Full text | **20,504** |
| **18** | Attachment OR “parent-child relationship*” OR “parent-child connection*” OR “attachment style*” OR “reactive attachment” OR “secure attachment” OR “insecure attachment” OR “avoidant attachment” OR “attachment rupture” OR “dismissive attachment” OR “disorganised attachment” OR “disorganized attachment” | Date: Since 2000;  Language: English | Title, Abstract | **11,088** |
| **19** | Trauma OR PTSD OR “post-traumatic stress disorder” OR “posttraumatic stress disorder” OR “trauma-informed” OR trauma OR stress OR “adverse child experience*” OR divorce OR war OR assault* OR abuse* OR neglect OR “household dysfunction” OR “physical abuse” OR “emotional abuse” OR “sexual abuse” OR “physical neglect” OR “emotional neglect” OR “mental illness” OR “incarcerated relative” OR “mother treated violently” OR “domestic violence” OR “substance abuse” | Date: Since 2000;  Language: English | Full text | **130,579** |
| **20** | Trauma OR PTSD OR “post-traumatic stress disorder” OR “posttraumatic stress disorder” OR “trauma-informed” OR trauma OR stress OR “adverse child experience*” OR divorce OR war OR assault* OR abuse* OR neglect OR “household dysfunction” OR “physical abuse” OR “emotional abuse” OR “sexual abuse” OR “physical neglect” OR “emotional neglect” OR “mental illness” OR “incarcerated relative” OR “mother treated violently” OR “domestic violence” OR “substance abuse” | Date: Since 2000;  Language: English | Title, Abstract | **102,171** |
| **21** | “sensory-regulation” OR “sensory processing difficult*” OR “sensory processing disorder*” | Date: Since 2000;  Language: English | Full text | **95** |
| **22** | “sensory-regulation” OR “sensory processing difficult*” OR “sensory processing disorder*” | Date: Since 2000;  Language: English | Title, Abstract | **74** |
| **23** | “physical wellbeing” OR “physical well being” | Date: Since 2000;  Language: English | Full text | **604** |
| **24** | “physical wellbeing” OR “physical well being” | Date: Since 2000;  Language: English | Title, Abstract | **599** |
| **25** | Indigenous OR aboriginal OR “Torres Strait Islander” | Date: Since 2000;  Language: English | Full text | **4,857** |
| **26** | Indigenous OR aboriginal OR “Torres Strait Islander” | Date: Since 2000;  Language: English | Title, Abstract | **4,257** |
| **27** | pediatric* OR paediatric* OR child* OR kindergarten* OR "elementary school*" OR "nursery school*" OR schoolchild* OR youth* | Date: Since 2000;  Language: English | Full text | **303,622** |
| **28** | pediatric* OR paediatric* OR child* OR kindergarten* OR "elementary school*" OR "nursery school*" OR schoolchild* OR youth* | Date: Since 2000;  Language: English | Title, Abstract | **280,596** |
| **29** | "meta-analys*" OR "systematic review*" | Date: Since 2000;  Language: English | Full text | **25,998** |
| **30** | "meta-analys*" OR "systematic review*" | Date: Since 2000;  Language: English | Title, Abstract | **21,208** |
| **31** | 2 AND 4 AND 28 AND 30 |  |  | **284** |
| **32** | 2 AND 6 AND 28 AND 30 |  |  | **786** |
| **33** | 2 AND 8 AND 28 AND 30 |  |  | **276** |
| **34** | 2 AND 10 AND 28 AND 30 |  |  | **521** |
| **35** | 2 AND 12 AND 28 AND 30 |  |  | **364** |
| **36** | 2 AND 14 AND 28 AND 30 |  |  | **31** |
| **37** | 2 AND 16 AND 28 AND 30 |  |  | **96** |
| **38** | 2 AND 18 AND 28 AND 30 |  |  | **50** |
| **29** | 2 AND 20 AND 28 AND 30 |  |  | **406** |
| **36** | 2 AND 22 AND 28 AND 30 |  |  | **2** |
| **37** | 2 AND 24 AND 28 AND 30 |  |  | **2** |
| **38** | 2 AND 26 AND 28 AND 30 |  |  | **11** |
| **39** | 27 OR 28 OR 29 OR 30 OR 31 OE 32 OR 33 OR 34 OR 35 OR 36 OR 37 OR 38 |  |  | **1,713** |
| **VIOLENCE AND ABUSE ABSTRACTS** | | | | |
| **Search #** | **Key words** | **Restrictions** | **Search Field** | **# of Results** |
| **1** | “family-focused intervent*” OR “early intervent*” OR prevent* OR transdiagnostic OR “targeted group” OR “group therap*” OR “peer group” OR “peer support” OR “targeted therap*” OR “targeted intervent*” OR “indicated intervent*” OR intervent* OR treat* OR “child intervent*” OR “school-based intervent*” OR “parent-child intervent*” OR “teacher-led program*” OR “teacher-led intervent*” OR “parent-led program*” OR “parent-led intervent*” OR “clinician-led program*” OR “clinician-led intervent*” | Date: Since 2000;  language: English | Full text | **13,499** |
| **2** | “family-focused intervent*” OR “early intervent*” OR prevent* OR transdiagnostic OR “targeted group” OR “group therap*” OR “peer group” OR “peer support” OR “targeted therap*” OR “targeted intervent*” OR “indicated intervent*” OR intervent* OR treat* OR “child intervent*” OR “school-based intervent*” OR “parent-child intervent*” OR “teacher-led program*” OR “teacher-led intervent*” OR “parent-led program*” OR “parent-led intervent*” OR “clinician-led program*” OR “clinician-led intervent*” | Date: Since 2000;  language: English | Title, Abstract | **10,629** |
| **3** | “mental health” OR “behavioural problem*” OR “behavioral problem*” OR “emotional problem*” OR “social problem*” | Date: Since 2000;  language: English | Full text | **5,104** |
| **4** | “mental health” OR “behavioural problem*” OR “behavioral problem*” OR “emotional problem*” OR “social problem*” | Date: Since 2000;  language: English | Title, Abstract | **2,575** |
| **5** | depress* OR mood OR internalizing OR internalizing OR sad* OR low OR unhapp* | Date: Since 2000;  language: English | Full text | **4,781** |
| **6** | depress* OR mood OR internalizing OR internalizing OR sad* OR low OR unhapp* | Date: Since 2000;  language: English | Title, Abstract | **4,455** |
| **7** | Anxi* OR internalizing OR internalizing OR worr* | Date: Since 2000;  language: English | Full text | **1,489** |
| **8** | Anxi* OR internalizing OR internalizing OR worr* | Date: Since 2000;  language: English | Title, Abstract | **1,353** |
| **9** | Conduct OR CD OR aggress* OR “oppositional defiant disorder*” OR “oppositional defiant” OR oppositional OR ODD OR externalizing OR externalizing OR misbehaviour OR misbehavior OR fight* OR cheating OR violen* OR defian* OR ang* OR argu* | Date: Since 2000;  language: English | Full text | **23,540** |
| **10** | Conduct OR CD OR aggress* OR “oppositional defiant disorder*” OR “oppositional defiant” OR oppositional OR ODD OR externalizing OR externalizing OR misbehaviour OR misbehavior OR fight* OR cheating OR violen* OR defian* OR ang* OR argu* | Date: Since 2000;  language: English | Title, Abstract | **17,436** |
| **11** | ADHD OR attention OR “attention deficit” OR hyper* OR concentrat* | Date: Since 2000;  language: English | Full text | **2,471** |
| **12** | ADHD OR attention OR “attention deficit” OR hyper* OR concentrat* | Date: Since 2000;  language: English | Title, Abstract | **2,393** |
| **13** | “emotion dysregulation” OR “emotion regulation” OR “emotional regulation” OR “affect regulation” OR “self regulation” OR tantrum* OR outburst* OR meltdown OR “emotion coaching” | Date: Since 2000;  language: English | Full text | **407** |
| **14** | “emotion dysregulation” OR “emotion regulation” OR “emotional regulation” OR “affect regulation” OR “self regulation” OR tantrum* OR outburst* OR meltdown OR “emotion coaching” | Date: Since 2000;  language: English | Title, Abstract | **310** |
| **15** | “social skill*” OR “friendship problem*” OR bully* OR bulli* OR “interpersonal problem*” OR “social problem*” OR “peer problem*” OR “peer relation*” OR “social relation*” OR connection OR “peer skills” OR “prosocial” | Date: Since 2000;  language: English | Full text | **2,678** |
| **16** | “social skill*” OR “friendship problem*” OR bully* OR bulli* OR “interpersonal problem*” OR “social problem*” OR “peer problem*” OR “peer relation*” OR “social relation*” OR connection OR “peer skills” OR “prosocial” | Date: Since 2000;  language: English | Title, Abstract | **1,825** |
| **17** | Attachment OR “parent-child relationship*” OR “parent-child connection*” OR “attachment style*” OR “reactive attachment” OR “secure attachment” OR “insecure attachment” OR “avoidant attachment” OR “attachment rupture” OR “dismissive attachment” OR “disorganised attachment” OR “disorganized attachment” | Date: Since 2000;  language: English | Full text | **1,060** |
| **18** | Attachment OR “parent-child relationship*” OR “parent-child connection*” OR “attachment style*” OR “reactive attachment” OR “secure attachment” OR “insecure attachment” OR “avoidant attachment” OR “attachment rupture” OR “dismissive attachment” OR “disorganised attachment” OR “disorganized attachment” | Date: Since 2000;  language: English | Title, Abstract | **530** |
| **19** | Trauma OR PTSD OR “post-traumatic stress disorder” OR “posttraumatic stress disorder” OR “trauma-informed” OR trauma OR stress OR “adverse child experience*” OR divorce OR war OR assault* OR abuse* OR neglect OR “household dysfunction” OR “physical abuse” OR “emotional abuse” OR “sexual abuse” OR “physical neglect” OR “emotional neglect” OR “mental illness” OR “incarcerated relative” OR “mother treated violently” OR “domestic violence” OR “substance abuse” | Date: Since 2000;  language: English | Full text | **18,861** |
| **20** | Trauma OR PTSD OR “post-traumatic stress disorder” OR “posttraumatic stress disorder” OR “trauma-informed” OR trauma OR stress OR “adverse child experience*” OR divorce OR war OR assault* OR abuse* OR neglect OR “household dysfunction” OR “physical abuse” OR “emotional abuse” OR “sexual abuse” OR “physical neglect” OR “emotional neglect” OR “mental illness” OR “incarcerated relative” OR “mother treated violently” OR “domestic violence” OR “substance abuse” | Date: Since 2000;  language: English | Title, Abstract | **12,840** |
| **21** | “sensory-regulation” OR “sensory processing difficult*” OR “sensory processing disorder*” | Date: Since 2000;  language: English | Full text | **0** |
| **22** | “sensory-regulation” OR “sensory processing difficult*” OR “sensory processing disorder*” | Date: Since 2000;  language: English | Title, Abstract | **0** |
| **23** | “physical wellbeing” OR “physical well being” | Date: Since 2000;  language: English | Full text | **12** |
| **24** | “physical wellbeing” OR “physical well being” | Date: Since 2000;  language: English | Title, Abstract | **12** |
| **25** | Indigenous OR aboriginal OR “Torres Strait Islander” | Date: Since 2000;  language: English | Full text | **343** |
| **26** | Indigenous OR aboriginal OR “Torres Strait Islander” | Date: Since 2000;  language: English | Title, Abstract | **244** |
| **27** | pediatric* OR paediatric* OR child* OR kindergarten* OR "elementary school*" OR "nursery school*" OR schoolchild* OR youth* | Date: Since 2000;  language: English | Full text | **12,006** |
| **28** | pediatric* OR paediatric* OR child* OR kindergarten* OR "elementary school*" OR "nursery school*" OR schoolchild* OR youth* | Date: Since 2000;  language: English | Title, Abstract | **9,517** |
| **29** | "meta-analys*" OR "systematic review*" | Date: Since 2000;  language: English | Full text | **1,180** |
| **30** | "meta-analys*" OR "systematic review*" | Date: Since 2000;  language: English | Title, Abstract | **880** |
| **31** | 2 AND 4 AND 28 AND 30 |  |  | **29** |
| **32** | 2 AND 6 AND 28 AND 30 |  |  | **38** |
| **33** | 2 AND 8 AND 28 AND 30 |  |  | **12** |
| **34** | 2 AND 10 AND 28 AND 30 |  |  | **90** |
| **35** | 2 AND 12 AND 28 AND 30 |  |  | **14** |
| **36** | 2 AND 14 AND 28 AND 30 |  |  | **2** |
| **37** | 2 AND 16 AND 28 AND 30 |  |  | **25** |
| **38** | 2 AND 18 AND 28 AND 30 |  |  | **6** |
| **29** | 2 AND 20 AND 28 AND 30 |  |  | **65** |
| **36** | 2 AND 22 AND 28 AND 30 |  |  | **0** |
| **37** | 2 AND 24 AND 28 AND 30 |  |  | **0** |
| **38** | 2 AND 26 AND 28 AND 30 |  |  | **0** |
| **39** | 27 OR 28 OR 29 OR 30 OR 31 OE 32 OR 33 OR 34 OR 35 OR 36 OR 37 OR 38 |  |  | **148** |

**Supplementary Material 2**

**Inclusion and Exclusion Criteria**

Inclusion of articles in the review was subject to the following criteria:

a. The mean age of participants was between 4- to 9-year-old. If the authors did not report on mean age of participants, although did refer to 'childhood' or children up to 12-years-old in discussing the results, these articles were included. In reviews that only included child grade (and not age), the study was only considered if no other exclusion criteria were met, and we could be sure that the average grade would align with the average age

b. The review adopted a systematic review and/or meta-analysis methodology and was published in the English language in a peer-reviewed journal.

c. The review discussed intervention efficacy in terms of the extent to which immediate or longer-term outcomes were achieved.

d. Mental health problems were defined broadly to include: anxiety, obsessive compulsive disorder (OCD), depression, suicide/self-harm, conduct problems, sleep problems, emotion dysregulation, ADHD, social skills problems, attachment problems, childhood trauma, sensory regulation, or social-emotional wellbeing. We did not include interventions that were specifically designed to target Autism Spectrum Disorder (ASD) unless they targeted any of the above mental health problems specifically in children with ASD.

e. The review included interventions in the following settings: school, community, clinic, foster-care or disaster settings; or interventions targeting Indigenous populations in Australia or trauma interventions relevant to the Australian context (e.g., refugees, floods, natural disasters, homelessness, sexual abuse, bereavement).

f. The review included indicated or selective interventions and treatments (i.e., interventions for children at risk for, showing signs of, or diagnosed with a mental health condition).

g. The review included interventions where the primary aim was to improve child mental health outcomes broadly (i.e., social, emotional and behavioral problems) or one or more of the outcomes highlighted in point (d) above.

Reviews were excluded if they met any of the following criteria:

a. Full text Criteria not met:

- Reviews were excluded where the full text in English was unable to be sourced.

b. Age criteria not met:

- Reviews were excluded where the mean overall age of participants was < 4 years or > 9 years.
- Where the mean overall age of participants was not reported, reviews were excluded if the mean ages or ranges reported for the individual studies were primarily < 4 years or > 9 years in majority of the studies AND where age was not examined as a moderator or in subgroup analyses.
- Reviews were excluded where the mean ages were not reported for individual studies or if the mean ages reported for the individual studies were primarily < 4 years or > 9 years AND where the authors referred to the participants as 'youth' or 'children and adolescents'.

c. Study criteria not met:

- Reviews that did not use a systematic review and/or meta-analysis methodology were excluded.
- Reviews that did not discuss intervention efficacy in terms of pre-post improvements in children's mental health outcomes were excluded.
- Economic evaluations of interventions were excluded.

d. Problem criteria not met:

- Reviews that included interventions for bipolar disorder, psychosis, and eating disorders were excluded.
- Reviews that included interventions for tic disorders (e.g., trichotillomania) or broad interventions for ASD were excluded.

e. Setting criteria not met:

- Reviews that included interventions conducted in hospital settings, prison settings or juvenile justice settings were excluded.

f. Intervention type criteria not met:

- Reviews that included interventions targeting mental health problems in children with comorbid physical health problems or intellectual disabilities were excluded.
- Reviews that included interventions targeting child maltreatment or family violence were excluded.
- Reviews that included interventions that only covered medication, diet, and/or physical activity or exercise were excluded.
- Reviews that included universal/school-wide/classroom-wide interventions and did not include subgroup analyses for indicated/targeted interventions were excluded.
- Reviews that included bullying interventions not focused specifically on child mental health outcomes were excluded.

g. Grey literature
